# Systemic Disease-Induced Salivary Biomarker Profiles in Mouse Models of Melanoma and Non-Small Cell Lung Cancer

**DOI:** 10.1371/journal.pone.0005875

**Published:** 2009-06-11

**Authors:** Kai Gao, Hui Zhou, Lei Zhang, Jin Wook Lee, Qing Zhou, Shen Hu, Lawrence E. Wolinsky, James Farrell, Guido Eibl, David T. Wong

**Affiliations:** 1 School of Dentistry & Dental Research Institute, University of California Los Angeles, Los Angeles, California, United States of America; 2 Department of Statistics, University of California Los Angeles, Los Angeles, California, United States of America; 3 Jonsson Comprehensive Cancer Center, University of California Los Angeles, Los Angeles, California, United States of America; 4 Molecular Biology Institute, University of California Los Angeles, Los Angeles, California, United States of America; 5 Division of Oral Biology, University of California Los Angeles, Los Angeles, California, United States of America; 6 Department of Digestive Diseases, University of California Los Angeles, Los Angeles, California, United States of America; 7 Department of General Surgery, David Geffen School of Medicine, University of California Los Angeles, Los Angeles, California, United States of America; 8 Henry Samueli School of Engineering and Applied Science, University of California Los Angeles, Los Angeles, California, United States of America; Harvard Institute of Medicine, United States of America

## Abstract

**Background:**

Saliva (oral fluids) is an emerging biofluid poised for detection of clinical diseases. Although the rationale for oral diseases applications (e.g. oral cancer) is intuitive, the rationale and relationship between systemic diseases and saliva biomarkers are unclear.

**Methodology/Principal Findings:**

In this study, we used mouse models of melanoma and non-small cell lung cancer and compared the transcriptome biomarker profiles of tumor-bearing mice to those of control mice. Microarray analysis showed that salivary transcriptomes were significantly altered in tumor-bearing mice vs. controls. Significant overlapping among transcriptomes of mouse tumors, serum, salivary glands and saliva suggests that salivary biomarkers have multiple origins. Furthermore, we identified that the expression of two groups of significantly altered transcription factors (TFs) Runx1, Mlxipl, Trim30 and Egr1, Tbx1, Nr1d1 in salivary gland tissue of melanoma-bearing mice can potentially be responsible for 82.6% of the up-regulated gene expression and 62.5% of the down-regulated gene expression, respectively, in the saliva of melanoma-bearing mice. We also showed that the ectopic production of nerve growth factor (NGF) in the melanoma tumor tissue as a tumor-released mediator can induce expression of the TF Egr-1 in the salivary gland.

**Conclusions:**

Taken together, our data support the conclusion that upon systemic disease development, significant changes can occur in the salivary biomarker profile. Although the origins of the disease-induced salivary biomarkers may be both systemic and local, stimulation of salivary gland by mediators released from remote tumors plays an important role in regulating the salivary surrogate biomarker profiles.

## Introduction

Saliva harbors a wide spectrum of proteins/peptides, nucleic acids, electrolytes, and hormones that originate in multiple local and systemic sources. The biochemical and physicochemical properties of saliva support its important functions in oral health such as food digestion, antibacterial activity, and maintenance of the integrity of the teeth [1], [2]. For example, xerostomia is an oral disease caused by a dysfunction of salivary glands, which is accompanied by reduced or absent secretion of saliva and is the cause of rampant caries and mucositis.

Diagnostically, a number of findings in the past decade have prompted interest in the use of saliva as a source of biomarkers. The soluble fragment of c-erbB-2 was detectable in the saliva of breast cancer patients but not in healthy controls or patients bearing benign tumors [3]. Levels of hormones (e.g. cortisol, oxytocin) and drugs (e.g. cisplatin, nicotine, methadone) in saliva reflect their concentration in serum [4], [5], [6]. In 2004 saliva-based HIV detection was approved by the US Food and Drug Administration (FDA).

A significant boost to the scientific foundation and infrastructure of salivary diagnostics research came six years ago when the National Institute of Dental & Craniofacial Research (NIDCR) made a significant investment toward developing the use of saliva as a diagnostic tool. Saliva has since become a biofluid that is poised for translational and clinical applications. Of note is the maturation of the salivary proteome, the first implement in the diagnostic toolbox for saliva-based diagnostics. We now know there are 1166 proteins in human saliva, the functions of which range from structural binding to participation in diverse biological processes [7]. A second diagnostic resource in saliva has since emerged, the salivary transcriptome. Using the salivary transcriptome as a diagnostic tool, a set of 185 mRNAs was identified as “normal salivary core transcripts” (NSCT) [8]. Moreover, the salivary transcriptome has been demonstrated to be clinically discriminatory for detecting oral cancer and Sjögren's syndrome (SS). The combination of seven salivary transcripts biomarkers (*IL8, IL1B, DUSP1, HA3, OAZ1, S100P, and SAT*) can be used to distinguish between the saliva of patients with oral squamous cell carcinoma (OSCC) and that of controls with 91% sensitivity and specificity [9]; whereas five salivary proteomic markers (M2BP, CD59, Catalase, MRP-14 and Profilin) collectively show a 93% sensitivity and specificity respectively to detect oral cancer using saliva [10]. Another study showed that 27 mRNAs and 16 peptides in saliva samples of SS patients were significantly up- or down-regulated [11]. The technology of salivary transcriptome has recently been advanced to the exon level with the capacity to comprehensively profile the salivary transcriptome using an exon-based technology [12].

There are many advantages to use saliva as a clinical diagnostic biofluid. Sample collection is simple, non-invasive, and causes little anxiety on the part of patients. The use of saliva also offers a cost-effective approach for large-scale screens [6].

**Figure 1:**
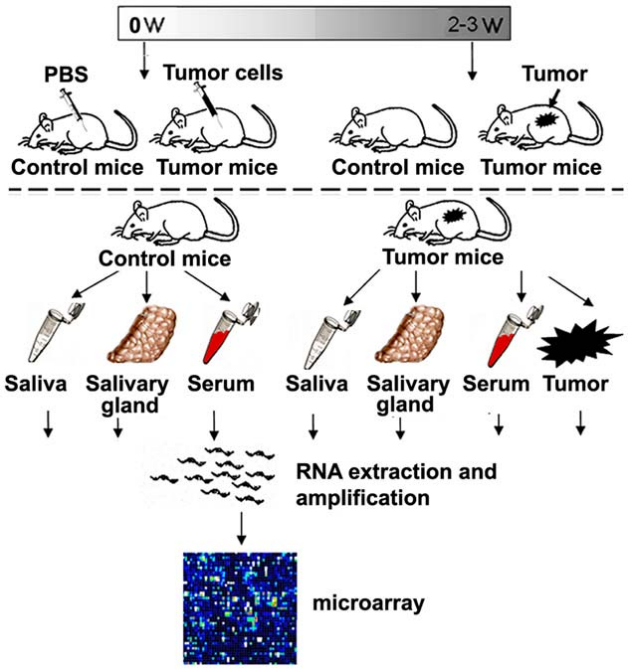
The Flowchart of animal experiments. Mice (either C57BL/6 mice or DBA/2 mice) were randomly divided into two groups as follows: control group (control mice) and tumor group (tumor mice) (15 animals per group). PBS was injected into control mice while mice in tumor group were injected with tumor cells. Tumors establishment took ∼3 weeks. Saliva, salivary gland, serum and tumor tissues were collected from each mouse. Five mice each were pooled into one group and processed to profile their transcriptome by the expression microarrays.

The use of saliva for detection of oral diseases has been confirmed, but its use for systemic disease is largely unclear. While reports have described the detection of biomarkers of systemic cancer in saliva (e.g. c-erb2 in breast cancer patients), the mechanisms underlying this phenomenon remain unsubstantiated. The goal of this study was to explore the scientific evidence and provide a rationale for the use of saliva for systemic disease detection. We used syngeneic mouse tumor models to develop tumors remote from the oral cavity and used the salivary transcriptome as the biomarker profile readout (Fig. 1). The salivary transcriptome has been validated as a scientifically credible and substantiated biomarker source in saliva [8], [9], [11], [12], [13], [14], [15], [16], [17], which permits the high throughput analyses and read-out necessary for the studies.

## Results

### Significant disease-induced differences between salivary transcriptome biomarker profiles in tumor-bearing and control mice

**Figure 2:**
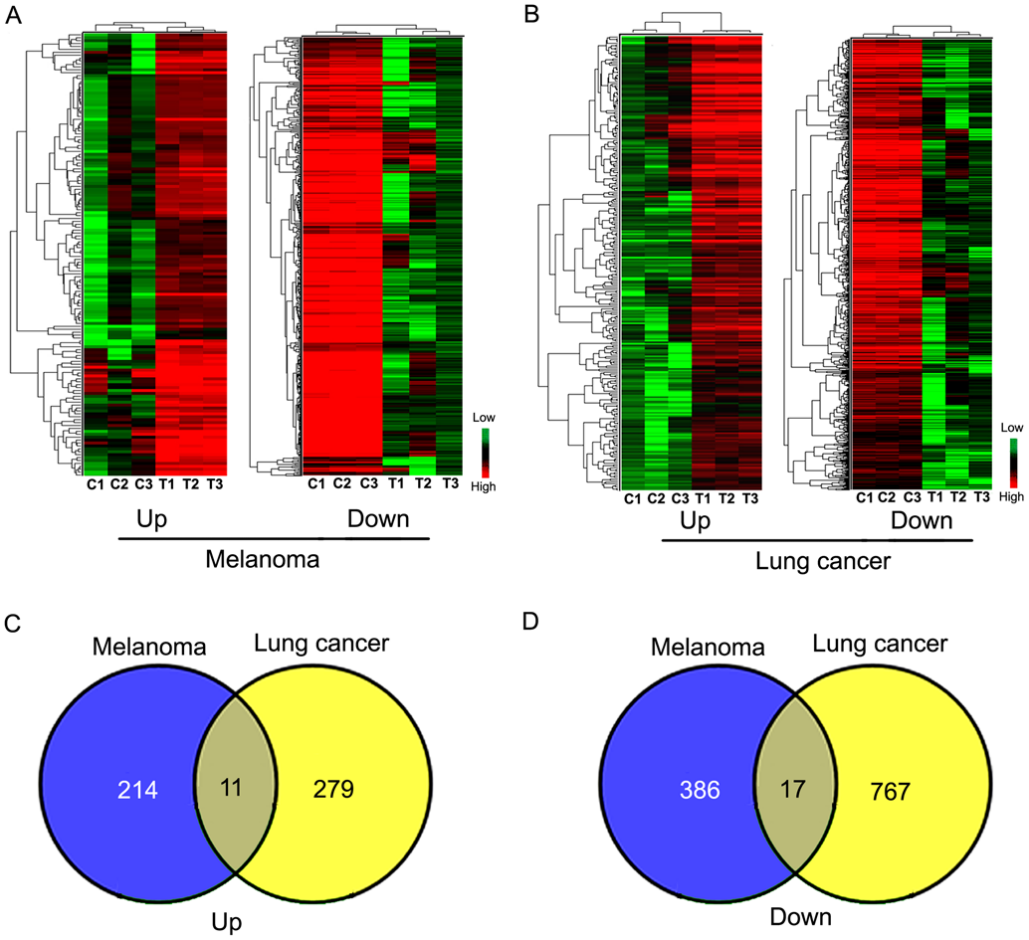
Expression profilings of saliva in the melanoma and lung cancer mouse models. *A*, Cluster analysis of 152 up-regulated known genes (representing 225 probsets, the left panel) and 359 down-regulated known genes (representing 403 probsets, the right panel) differentially expressed in saliva of melanoma mice vs. control mice (P-value<0.05; fold change ≥2). The expression profiles were standardized to have zero mean and unit standard deviation. Red and green represent high and low expression levels after standardization, respectively. *B*, Cluster analysis of 290 up-regulated and 784 down-regulated probesets differentially expressed in saliva of lung carcinoma mice vs. control mice (P-value<0.05; fold change ≥2). *C* and *D*, Overlapping of differentiated gene expression between the melanoma model and lung cancer model. *C*, overlapping of 225 up-regulated genes in the melanoma model and 290 up-regulated genes in the lung cancer model. *D*, overlapping of 403 down-regulated genes in the melanoma model and 784 down-regulated genes in the lung cancer model.

To assess whether the salivary transcriptome biomarker profile changes upon development of a remote tumor, we performed microarray analysis to compare the transcriptome biomarker profiles in saliva of control mice (three groups, 5 mice in each group) with tumor-bearing (melanoma or lung) mice (three groups, 5 mice in each group) (Fig. 1). It is necessary to have five mice in each tumor or control group in order to pool sufficient saliva for RNA isolation. Biomarker selection criteria were set as fold change>2 and *P*<0.05. We identified 152 significantly up-regulated known genes and 359 significantly down-regulated known genes (Fig. 2A, Table S3 and S4) in the saliva of melanoma-bearing mice compared to control mice. Similarly, we found 290 significantly up-regulated transcripts and 784 significantly down-regulated transcripts (Fig. 2B,.Table S5 and S6) in the saliva of lung cancer-bearing mice compared to control mice.

We also overlapped the differentiated gene expression in the saliva of melanoma mice with that of lung cancer mice. Comparing up-regulated or down-regulated salivary genes in two models, respectively, we found 11 up-regulated (Fig. 2C) and 17 down-regulated (Fig. 2D) transcripts exist in both models. However, considering the different genetic background and cancer cell lines in the two mouse modes, it is not surprised that only a fraction of total altered genes was overlapped (4.8% (11/225) or 3.8% (11/290) up-regulated genes in melanoma model or lung cancer model, respectively; 4.2% (17/403) or 2.1% (17/784) down-regulated transcripts in the two models, respectively).

### Multiple sources contribute to the tumor-induced salivary mRNA profile alteration

**Figure 3:**
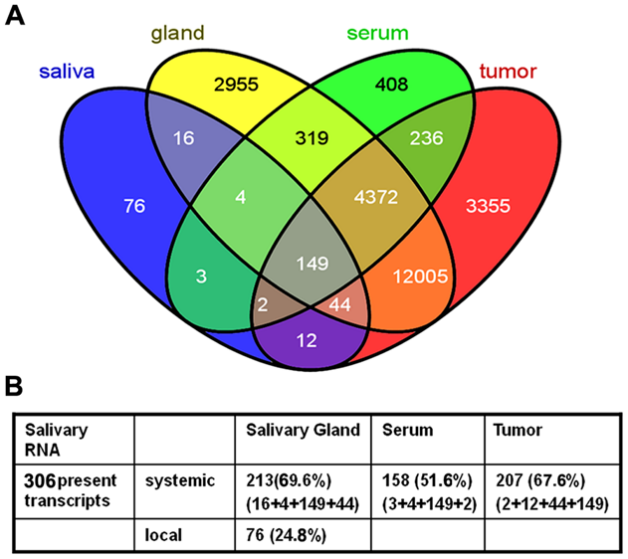
Overlapped gene expression profilings among saliva, salivary gland, serum and tumor in the melanoma mouse model. *A*, Overlapping transcripts present in saliva, salivary gland, serum and tumor in the melanoma-bearing mice. *B*, Of the 306 salivary transcripts, 69.6% were present in salivary gland, 51.6% present in serum, 67.6% present in tumor (melanoma), and 24.8% may originate from oral cavity (local).

To examine the possible sources contributing to the salivary transcriptome alterations in mice in response to systemic disease, we filtered the expression profiling data of the melanoma-bearing mice (tumor, serum, salivary gland and saliva) to select “present” mRNA with a *P* value<0.001 and an intensity value>200. In the melanoma model, 20175, 5493, 19904, and 306 transcripts were identified in the tumor, serum, salivary gland and saliva, respectively (Fig. 3A). After overlapping all the present genes from tumor, serum, salivary gland and saliva, Fig. 3B showed that of the 306 transcripts present in saliva, 67.6% are also present in melanoma-tumor tissue, 51.6% are also present in serum and 69.6% are also present in salivary gland. These data indicate that the origins of the present transcriptome in saliva may be associated with various compartments in the whole body constituting totally ∼75.2% of the 306 salivary transcripts. In addition, 24.8% of the 306 transcripts did not overlap with genes in tumor, salivary gland and serum, suggesting that they may originate from the oral cavity.

### Altered expression of transcription factors (TFs) in salivary glands of melanoma-bearing mice correlates with altered transcription factor-mediated gene expression changes in mouse saliva

Since the salivary transcriptome was clearly altered in tumor-bearing vs. control mice, we hypothesized that the tumors behave like endocrine organs in that they secrete mediators (hormones, lymphokines, cytokines) which can affect the activity of TF in salivary glands and thereby induce up or down-regulation of transcripts levels in saliva.

Although the two mouse cancer models in this study are well-established [34], [35], the melanoma mouse model simulates human melanoma better than lung cancer model theoretically and pathologically because both human melanoma and this mouse melanoma occur subcutaneously. Therefore, we used the melanoma-bearing C57BL/6 mice as a working model to test our hypothesis.

**Figure 4:**
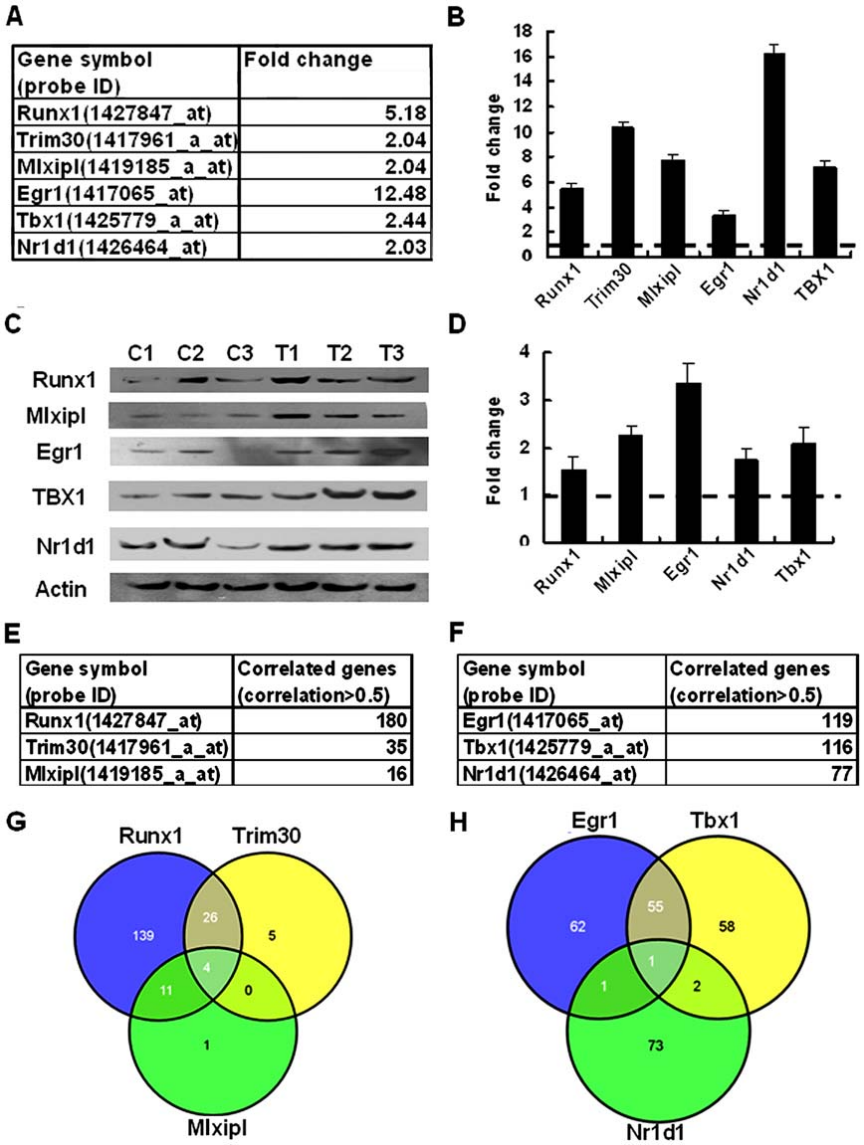
Transcription factors (TFs) in the salivary gland were up-regulated and correlated with the expression of a number of genes in saliva of the melanoma mouse model. *A*, The 6 TFs (*Runx1, Trim30, Mlxipl, Egr1, Nr1d1, TBX1*) are significantly expressed higher in the salivary gland of melanoma-bearing mice vs. control mice (P<0.05, Table S1). *B*, mRNA expression levels of these 6 TFs (*Runx1, Trim30, Mlxipl, Egr1, Nr1d1, TBX1*) in the salivary gland of melanoma mice vs. that of normal mice validated by qRCR. The horizontal dashed line indicates the levels of gene expression in control mice, which is arbitrarily set to 1. The columns represent gene expression levels in the salivary gland of melanoma mice relative to control mice. Experiments were done in triplicates; bars, SD. *C*, Expression levels of five TFs (Runx1, Mlxipl, Egr1, Nr1d1, and TBX1) in the salivary gland tissues of control mice and melanoma mice were measured by immunoblotting. (C1, C2, C3 and T1, T2, T3 are the same batch of tissues used in the microarray assay). Note that commercial antibody was not available for murine Trim30. *D*, Relative protein expression levels of the above five TFs in melanoma mice vs. control mice. Signal intensity of the blot in Figure 4*C* was quantified by Image J software (NIH). The horizontal dashed line indicates the expression levels of these 5 TFs in control mice, which is arbitrarily set to 1. Columns show that the relative protein levels of the 5 TFs in tumor-bearing mice comparing to control mice; bars, SD. *E* and *F*, The expression of 6 TFs (*Runx1, Trim30, Mlxipl, Egr1, Nr1d1, TBX1*) in the salivary gland of melanoma mice were correlated with differentiated gene expression in the mouse saliva. Three of them (Runx1, Trim30 and *Mlxipl*) can be potentially responsible for 180, 35 and 16 of the up-regulated salivary transcripts in melanoma mice (*P*<0.05), respectively, while the other 3 TFs (*Egr1, Tbx1 and Nr1d1*) are potentially correlated with 119, 116 and 77 down-regulated salivary transcripts (*P*<0.05). *G*, The expression of the 3 TFs (Runx1, Trim30 and Mlxipl) totally correlated with 82.6% ((180+5+1)/225 = 82.6%) up-regulated gene expression in melanoma mouse saliva by overlapping all salivary genes which are correlated with these 3 TFs from Fig. 4*E*. *H*, The expression of the other 3 TFs (Egr1, TBX1 and Nr1d1) totally correlated with 62.5% ((119+58+2+73)/403 = 62.5%) down-regulated gene expression in the saliva of melanoma mice by overlapping all salivary genes which are correlated with these 3 TFs from Fig. 4*F*.

We first compared the gene expression profiles of salivary gland tissues in melanoma-bearing mice with control mice and identified a list of 46 significantly up-regulated TFs (fold change>2 and *P*<0.05) (Table S1). We then calculated the correlation coefficients between the expression profiles of these significantly altered TFs and the differentially expressed genes (both up- and down-regulated) in the saliva of the melanoma-bearing mice. The TFs were then ranked by the number of highly co-expressed genes whose correlation with the TF expression is >0.5. The 6 up-regulated TFs with highest ranking were RunX1 (runt related transcription factor 1), MLXIPL (musculus MLX interacting protein-like) and TRIM30 (tripartite motif protein 30) for upregulated salivary genes and Egr1 (Early growth factor-1), Tbx1 (T-box 1) and Nr1d1 (musculus nuclear receptor subfamily 1, group D, member 1) for down regulated salivary genes (Fig. 4A, E, F).

Next, the altered expression patterns of these salivary gland TFs were validated at both the transcription and protein levels by qPCR and immunoblotting. Figure 4*B* shows that the mRNA levels of the six TFs are increased from 3- to 16- fold in the salivary glands of melanoma-bearing mice compared to control mice. Immunoblotting detection using 5 commercially available murine TFs antibodies revealed 1.5 to 3.5-fold higher levels of these TFs expression in the salivary glands of melanoma-bearing mice than in control mice (Figure 4*C and 4D*). Then Figure 4*E and F* show that the above 6 TFs were associated with a number of differentiated gene expressions in mouse saliva. After overlapping the associated genes of each TF, it can be seen that the altered activities of the three TFs (RunX1, MLXIPL and TRIM30) can be potentially responsible for 83% of the up-regulated mRNAs in saliva (Fig. 4*G*) whereas the collective altered activities of Egr1, Tbx1 and Nr1d1 can potentially account for 63% of the down-regulated expression of salivary mRNAs (Fig. 4*H*).

### The Egr-1 signal pathway and detection of nerve growth factor (NGF) in melanoma tumor tissue and serum

**Figure 5:**
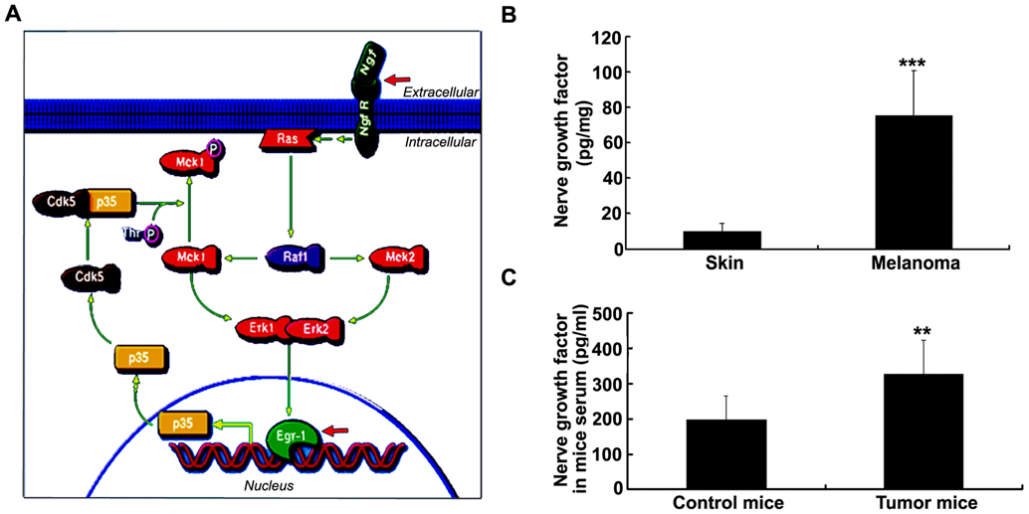
NGF was involved in the activation of TF Egr-1 and increased in the mouse serum. *A*, A pathway involving transcription factor Egr1. Nerve growth factor (NGF) can be an upstream factor of Egr1. *B*, Expression of NGF in mice skin and melanoma tissues measured by ELISA. Columns are absolute mean values of NGF concentration in tissues from five melanoma mice. ***, *P*<0.001. *C*, Expression of NGF in serum of control mice and tumor mice. Columns are absolute mean values of NGF concentration in serum from five control mice and five melanoma mice. Bar, SD. **, *P*<0.05

To investigate whether the developed tumors can mediate the altered expression of TFs in the salivary glands of tumor-bearing mice, we examined the NGF/Egr1 signal pathway because Egr1 was identified as an up-regulated TF in the salivary gland of melanoma-tumor mice. It is well-known that Egr-1 is a TF in the NGF signaling pathway [18], [19] (Fig. 5A). We therefore hypothesize that NGF is secreted into the circulation by the melanoma, circulates to the salivary gland where it activates the receptor-mediated signaling cascade leading to Egr-1 upregulation and induction of specific gene transcription and protein translation. Figure 5B shows that NGF is produced in melanoma tissue at a significantly higher levels than in normal skin (*P*<0.001). Figure 5C shows that NGF is also significantly higher in the serum of melanoma-bearing mice compared to control mice (*P*<0.05). Collectively these data suggest that melanoma can produce NGF and is secreted it into the bloodstream. Upon reaching the salivary glands, the increased NGF levels in the blood could then stimulate the increased expression of TFs such as Egr-1 leading to altered gene expression and protein profiles in the saliva of melanoma-bearing mice.

## Discussion

Studies have demonstrated the potential for use of saliva as a diagnostic biofluid in translational and clinical applications. As a biological sample, saliva is inexpensive and easily accessible by non-invasive means. The comprehensive knowledge base of the saliva's diagnostic composition offers a valuable and informative resource for biomarker discovery (www.skb.ucla.edu). Highly discriminatory salivary biomarkers for two oral diseases: oral cancer and Sjögren's syndrome have been identified and validated [9], [11]. However, the link between systemic diseases and saliva biomarkers is still unclear. In this study, we used mouse models of cancer to determine whether salivary biomarker profiles are affected by distal disease development. Our data demonstrated that salivary transcriptome profiles are significantly altered in mice bearing either of two tumors: melanoma and lung carcinoma (Fig. 2). Each tumor-type was associated with a different salivary transcriptome profile. In addition, our analysis of NGF production and the TF Egr1 suggest that the production of growth factors in the tumor tissue represents one mechanism whereby a distant tumor can alter the transcriptome of the salivary gland and hence the saliva. These findings also show that the salivary glands can play a key role in mediating tumor-induced alterations in saliva transcriptome biomarker profile. In addition to showing that disease-induced circulating biomarkers can find their way into the saliva, these findings suggest that disease-induced salivary gland surrogate biomarkers can have diagnostic value for the detection or monitoring of systemic diseases.

Since our first report on the salivary transcriptome [8], [9], we have been examining the origins of salivary mRNA, which appears to be different from the mRNAs found in other bodily fluids. The nucleic acids in serum of cancer patients are thought to be shed directly from the cancer cells or to be released as the result of cell lysis in damaged organs while the nucleic acids in urine may come from blood mRNA or DNA [20], [21]. In a study of human saliva, transcripts in the salivary transcriptome can be detected in all sources of saliva including the parotid gland, submandibular and sublingual glands, gingival crevicular fluids and oral epithelial cells [16]. It has recently been demonstrated that the majority of mRNAs in the salivary transcriptome has an AU-rich element (ARE) in their 3′UTR which confers stability by complexing with ARE-binding proteins [17]. In the present study, we compared the transcriptomes of the tumor, serum, salivary glands and saliva and found the salivary transcriptome in tumor-bearing mice highly overlapped to a great extent with the transcriptomes of salivary gland, serum and tumor. These analyses suggest that there may be multiple origins of salivary mRNA and/or that a complex systemic relationship may exist between the oral cavity and systemic health.

We investigated potential mechanisms by which the distal tumors mediate changes in salivary biomarker profiles in tumor-bearing mice. Previous studies have shown that systemic diseases or treatments can affect the function of the salivary gland resulting in changes in the composition of the saliva [22], [23], [24]. Salivary sodium and protein levels were elevated after interleukin-2 (IL-2) treatment in patients. One study using a mouse model also showed that levels of inflammatory factors such as IL-1beta increased in saliva after remote inflammation in the body [24]. As the salivary gland is the major source of saliva, we hypothesized that the salivary glands can be responsible for saliva specific biomarker alterations related to distal tumors. Since our experiment was carried out in triplicate, the Bayesian method may be applicable. However, this method requires specific model assumptions for the data. Finally the Expression console (Affymetrix, Inc.) and Dchip (http://biosun1.harvard.edu/complab/dchip/) software were applied in this study. For the melanoma syngeneic model, we identified 46 TFs are significantly up-regulated in the salivary glands of the melanoma-bearing mice which may lead to direct induction or suppression of gene expression. The relative fold changes of TF expression such as Egr1 and Nr1d1 are different between mRNA levels and protein levels, which may reflect translational modification or proteasomal degradation [25]. We then found that these altered expression of TFs is associated with the melanoma-induced transcriptome in saliva. We found that approximately 83% of the significantly up-regulated transcripts in the saliva can be accounted for by three TFs (Runx1, Trim30 and Mlxipl) (Fig. 4G) and 63% of the significantly down-regulated salivary transcripts can be accounted for by another three TFs (Egr1, TBX1 and Nr1d1) (Fig. 4H). Collectively these findings allow us to conclude that the salivary glands serve a previously unappreciated role as an organ monitoring systemic disease by inducing disease-specific TFs and altering expression of specific genes and translation of the corresponding proteins. These altered salivary mRNAs and proteins are disease-associated surrogate biomarkers that are secreted into glandular fluids and enter the oral cavity as whole saliva.

**Figure 6:**
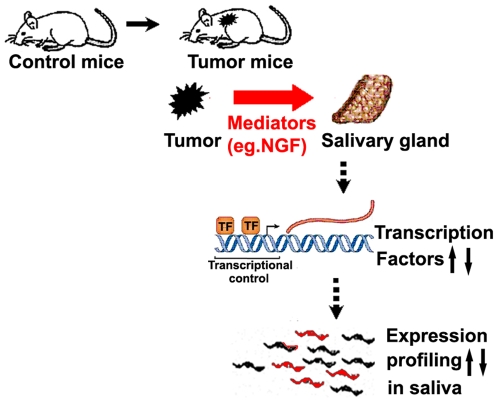
The working model: The relationship between the salivary transcriptome and the remote tumor. Mediators such as NGF secreted by remote tumors are transferred to salivary gland through blood to stimulate TFs expression and alter salivary mRNA profile.

Since the induction of TFs expression in the salivary glands occurred in mice with a distal tumor, we further hypothesized there are tumor-specific mediators that can effect the altered TG expression in the salivary glands. It is well-known that tumors ectopically express mediators that have systemic effects on distal organs and facilitate metastasis of cancer cells [26], [27]. Melanomas are known to ectopically express TGF-beta [28] while lung tumors ectopically express gonadotropins and other hormones [29], [30]. We investigated if one such signaling pathway can related to one of the identified TFs that can be responsible for induction or suppression of the salivary transcriptome in the mouse melanoma model. Indeed, NGF is known to stimulate expression of the TF Egr-1 through a well-studied signaling pathway (Fig. 5A). Using an NGF ELISA assay, we observed that the concentration of NGF in melanoma tissue and serum is significantly higher than in counterpart control tissues (Fig. 5B, C). These data suggest a biological scenario and rationale in which the developing mouse tumor secretes NGF into the circulation, where it circulates in the blood to the salivary glands and binds to NGF receptors expressed by salivary acinar cells, activating a signaling pathway that leads to the upregulation of Egr-1 mRNA and protein levels. It should be noted that melanoma cells are derived from melanocytes, which migrate from the neural crest during embryonic development. NGF can stimulate the proliferation and metastasis of melanoma cells [31]. On the other hand, NGF and its receptor (TrkA IR and TrkC IR) have been found in the salivary gland [32], [33]. Therefore, it is reasonable to propose that NGF secreted by melanoma tumor was transferred through blood and bound to its cognate receptors in the salivary gland, ultimately resulting in the stimulation of multiple TFs expression including Egr-1 (Fig. 6).

We also measured NGF levels in the tumor lysate of the mouse lung cancer model. While NGF was detectable, it was at a significantly lower level than the melanoma tumor lysate (20.9±4.3 pg/mg in lung tumor vs. 75.73±24 pg/mg in melanoma tissue, P<0.05, data not shown). In addition, only 11 up-regulated and 17 down-regulated transcripts were overlapped when we compared the salivary mRNA profile of the melanoma model to the lung cancer model (225 up-regulated or 403 down-regulated genes in the melanoma model vs. 290 up-regulated or 784 down-regulated transcripts in the lung cancer model, respectively, Fig. 2C and D). And 2 of those 17 overlapped down-regulated genes were correlated with Egr1 expression (data not shown). Collectively these data allow us to conclude that melanoma-derived NGF is a potentially important mediator in the downregulation of specific salivary transcriptome only in the melanoma-bearing mice.

While our report does not comprehensively demonstrate the mechanistic connection between systemic disease development and salivary biomarker alterations, it does begin to paint the picture for the concept that systemic networks exist in our body, which allows communication between distal diseases and the salivary glands. Signals transmitted through such networks can induce related signaling pathways that result in altered gene expression and protein translation and thereby produce disease-induced salivary biomarker profiles. We hypothesize that such disease-induced salivary gland-mediated transcriptomes and translational products can serve as valuable indicators of disease onset and/or progression. Therefore, the salivary gland can be considered as a reactive organ monitoring systemic diseases and saliva can be investigated as a biomarker-enriched disease-reflective biofluid. The local production and secretion of saliva from a single anatomical source (the salivary glands), and the fact that it can be harnessed simply and non-invasively as well as with relatively little discomfort for patients, provide a strong incentive for the continued investigation of salvia as a potential diagnostic indicator of systemic diseases.

## Materials and Methods

### Animals

Six- to eight-week-old DBA/2 mice and C57BL/6 mice were purchased from Jackson laboratory (Bar Harbor, ME) and housed in the Division of Laboratory Animal Medicine (DLAM) at the University of California at Los Angeles. The experimental protocols were approved by the Chancellor's Animal Research Committee (ARC) at the University of California at Los Angeles (UCLA).

### Cell lines

Murine cell lines KLN-205 and B16-F1 were obtained from the American Type Culture Collection (ATCC). KLN-205 is a non-small cell lung cancer (NSCLC) cell line originally established in a DBA/2 mice. Cells were cultured in MEM (GIBCO). And B16-F1, a C57BL/6–derived melanoma cell line, was maintained in DMEM (GIBCO) [34]. All cells were maintained in an atmosphere of 5% CO2 at 37°C.

### *In vivo* tumor models

Melanoma mouse model was induced by subcutaneous (s.c.) injection of 1×10^5^ B16-F1 cells in 0.1 ml PBS into the lower-right flank of C57BL/6 mice. The lung cancer model was established by s.c. injection of 2×10^5^ KLN-205 cells in DBA/2 mice [34], [35]. Control animals were injected with PBS alone. Established tumors were observed after 2–3 weeks (Fig. 1).

### Collection of mouse saliva, blood and tumor tissue

When tumors reached 15 mm in diameter saliva was collected and the mice were sacrificed. Mild anesthesia was induced by intramuscular (IM) injection of 1 ul/kg body weight of a solution containing 60 mg/ml ketamine (Phoenix Scientific, St. Joseph, MO) and 8 mg/ml xylazine (Phoenix Scientific). Mice were subcutaneously injected with pilocarpine (0.05 mg pilocarpine/100 g body weight) between ears to stimulated saliva secretion. Saliva was obtained from the oral cavity by micropipette and immediately placed in pre-chilled 1.5-ml microcentrifuge tubes. Collection was completed in 20 minutes [23] and samples were stored at −80°C until analyzed.

Blood was collected in BD Vacutainer tubes containing clot activator (BD Biosciences) and centrifuged at 1000× g for 10 min after mice were sacrificed[36]. Salivary gland and tumor tissue were removed from mice, snap-frozen in liquid nitrogen and stored at −80°C.

The experiment was carried out in triplicate. Mice (either 30 C57BL/6 mice or 30 DBA/2 mice) were randomly assigned to a control group and a tumor group equally (15 animals per group). Therefore, in the melanoma mouse model, the control group and treated group consist of 15 C57BL/6 mice, respectively (totally 30 mice). Saliva sample collected from each of 5 control mice was designated as C1, 2, 3. Saliva sample pooled from each of 5 treated mice was designated as T1, 2, 3. The tissue of salivary gland, serum and tumor was pooled together in the same order. Finally triplicate samples of each saliva, salivary gland, serum and tumor tissue in either control group or treated group were ready for the following RNA extraction and microarray process. We made the same design in the lung cancer model using 30 DBA/2 mice.

### RNA extraction and high-density oligonucleotide microarray analysis

Saliva, salivary gland, serum and tumor RNA were isolated using the RNeasy Mini Kit (Qiagen) as described previously [12]. There are 15 mice in the control group or tumor group (totally 30 C57BL/6 mice for melanoma mouse model, another 30 DBA/2 mice for lung cancer mouse model). Samples derived from 5 mice in each group were pooled and RNA extracted. The pooling is necessary because it ensures that sufficient salivary mRNA can be obtained for microarray analyses. Isolated total RNA was treated with recombinant DNase (Ambion, Austin, TX). For microarray analysis, mRNA from mouse saliva, gland, serum or tumor was linearly amplified using the RiboAmp RNA Amplification kit (Molecular Devices, Sunnyvale, CA). After purification, cDNA were in vitro transcribed and biotinylated using GeneChip Expression 3′-Amplification Reagents for in vitro transcription labeling (Affymetrix, Santa Clara, CA). The labeled RNAs (15 ug each) was subsequently fragmented and sent to UCLA microarray core facility for array hybridization and scanning. The GeneChip Mouse Genome 430 2.0 Array, which represents >39,000 transcripts and variants, was used for profiling analysis. All the raw data were imported into DNA-chip Analyzer software (http://www.biostat.harvard.edu/complab/dchip) for normalization and comparison. Microarray data has been uploaded to the GEO database (http://www.ncbi.nlm.nih.gov/geo/index.cgi). The access number is GSE13443

### Microarray data analysis

All microarray data were processed and normalized using the software dChip [37] to compute gene expression indexes. We conducted two-sample comparisons between gene expression in the tumor-treated and control groups for salivary gland and saliva, respectively. A gene was identified as up- (or down-) regulated if 1) the *P*-value of the two-sample t-test was <0.05 and 2) the ratio of its average expression level in tumor-bearing mice to that in control mice was >2 (or <0.5 for down-regulated genes). The heatmap was created by Cluster3.0 and Java gene Treeview software [38], [39].

Correlation analysis was carried out using expression profiling in the salivary gland of the melanoma mouse model. Among the up-regulated genes in salivary gland, 46 of them encode TFs (Table S1). We calculated the correlation coefficients between the expression profiles of these TFs and the differentially expressed (up- and down-regulated) genes in saliva in all samples. We ranked the TFs according to the number of highly coexpressed genes whose correlation with the TF expression is >0.5.

We also defined a probeset as present when it had a *P* value<0.001 and an intensity value>200 [12]. Pathway analysis was done using the Biocarta database (http://www.biocarta.com) and permission was obtained for use in the paper.

### Quantitative real-time PCR

PCR primers for six genes (Runx1, Trim30 Mlxipl, Egr1, Nr1d1, and TBX1) (Table S2) were designed using Primer Express 3.0 software (Applied Biosystems, Foster City, CA), with a melting temperature at 58–60°C. PCR was carried out in triplicate in reaction volumes of 10 µl using SYBR- Green Master Mix (Applied Biosystems) for 15 min at 95°C for initial denaturing, followed by 40 cycles of 95°C for 30 sec and 60°C for 30 sec in the ABI 7500HT Fast Real Time PCR system [40].

The threshold cycle (Ct) was obtained using the 7500 fast system software (Applied Biosystems) and was averaged. Relative expression levels (fold changes) were calculated according to the formula 2^−[(Te-Tn)-(Ce-Cn)]^. Te is the Ct cycle number of an interested gene such as Runx1 observed in a sample of salivary gland from the treated group, Tn is the Ct cycle number of the housekeeping genes GAPDH observed in the same sample while Ce is the average Ct cycle number of the same target gene such as Runx1 observed in a sample in the control group, Cn is the average Ct cycle number of housekeeping gene GAPDH in the same sample. The results obtained from relative expression levels were used for statistical analysis. Representative results are shown in Fig. 5C.

### Western blot and ELISA

Tissues were washed in PBS three times and lysed in triple detergent buffer (50 mM Tris-Cl [pH 8.0], 150 mM NaCl, 0.02% sodium azide, 0.1% SDS, 100 µg/ml phenylmethylsulphonyl fluoride, 1 µg/ml aprotinin, 1% Nonidet P-40, and 0.5% sodium deoxycholate) for 20 min on ice followed by sonication [41]. The lysate was centrifuged at 12,000× g for 10 min and the supernatant was collected. Protein concentration was determined using the DC Protein Assay (Bio-Rad, Hercules, CA). Proteins were separated on 10% polyacrylamide/SDS gels (Invitrogen, CA) and electroblotted onto polyvinylidene difluoride films. Films were blocked in 5% skimmed milk for 1 h and incubated with the indicated antibodies (Santa Cruz Biotechnology). (Fig. 5D). The signal intensity of the bands was measured using Image J software (NIH, Bethesda, MD). The intensity of a band representing the interested gene such as Runx1 was divided by the intensity of its corresponding ACTIN expression on the same blot. Then we compared the fold changes of each TF expression between the treated group and the control group (Fig. 5E).

The concentration of NGF in melanoma tissue and serum was measured by the NGF ELISA kit (Chemicon, Temecula, CA) and performed according to the manufacturer's instructions.

## Supporting Information

Table S1 (0.08 MB DOC)

Table S2 (0.04 MB DOC)

Table S3 (0.30 MB DOC)

Table S4 (0.51 MB DOC)

Table S5 (0.38 MB DOC)

Table S6 (0.98 MB DOC)
