# Supplementary material for: Systemic Disease-Induced Salivary Biomarker Profiles in Mouse Models of Melanoma and Non-Small Cell Lung Cancer
Source: PLoS One. 2009 Jun 11;4(6):e5875. doi: 10.1371/journal.pone.0005875 (PMC2691577; doi:10.1371/journal.pone.0005875)
Supplement: Table S1 — (0.08 MB DOC) [file pone.0005875.s001.doc]

**Supplementary Table 1.** Up-regulated expression of transcription factors in salivary gland of melanoma-bearing tumor mice vs. control mice

| Gene names | Accession | fold change | P value |
| --- | --- | --- | --- |
| Jun-B oncogene | NM_008416 | 4.13 | 0.014764 |
| SRY-box containing gene 2 | U31967 | 2.79 | 0.001058 |
| early growth response 1 | NM_007913 | 12.48 | 0.005338 |
| Jun oncogene | NM_010591 | 3.09 | 0.003963 |
| nuclear factor of kappa light polypeptide gene enhancer in B-cells inhibitor | AB026551 | 2.6 | 0.001561 |
| avian reticuloendotheliosis viral (v-rel) oncogene related B | NM_009046 | 2.63 | 0.019909 |
| tripartite motif protein 30 | BM240719 | 2.04 | 0.020405 |
| forkhead box F2 | NM_010225 | 2.82 | 0.043528 |
| interferon regulatory factor 2 | NM_008391 | 2.26 | 0.008263 |
| Williams-Beuren syndrome chromosome region 14 homolog (human) | AF245479 | 2.04 | 0.035223 |
| AE binding protein 2 | NM_009637 | 2.91 | 0.020431 |
| aryl hydrocarbon receptor nuclear translocator-like | BC011080 | 2.16 | 0.028171 |
| T-box 1 | AF326960 | 2.44 | 0.021346 |
| nuclear receptor subfamily 1, group D, member 1 | W13191 | 2.03 | 0.020302 |
| runt related transcription factor 1 | AB046930 | 5.18 | 0.042176 |
| transcription factor 7, T-cell specific | AI323642 | 3.21 | 0.020592 |
| RNA-binding region (RNP1, RRM) containing 2 | BB203348 | 3.16 | 0.033034 |
| ankyrin repeat domain 10 | NM_133971 | 2.03 | 0.00562 |
| Kruppel-like factor 2 (lung) | NM_008452 | 2.78 | 0.01967 |
| activating transcription factor 3 | BC019946 | 8.57 | 0.011987 |
| purine rich element binding protein A | NM_008989 | 2.01 | 0.015542 |
| inhibitor of DNA binding 2 | AK013239 | 2 | 0.011878 |
| Spi-B transcription factor | BM244106 | 6.56 | 0.022689 |
| zinc finger protein, subfamily 1A, 1 (Ikaros) | AV317621 | 7.4 | 0.030986 |
| Single-stranded DNA binding protein 3 | BM119756 | 2.23 | 0.001099 |
| special AT-rich sequence binding protein 1 | BB724383 | 2.38 | 0.016416 |
| Sequestosome 1 | BM237736 | 2.1 | 0.001276 |
| myeloid ecotropic viral integration site-related gene 1 | BB212184 | 3.14 | 0.003349 |
| Pre B-cell leukemia transcription factor 1 | BM114723 | 2.97 | 0.019324 |
| Nuclear factor I/A | BB453909 | 2.69 | 0.005909 |
| Friend leukemia integration 1 | BB555654 | 2.13 | 0.008821 |
| Nuclear factor I/B | BG173293 | 2.25 | 0.001506 |
| estrogen related receptor, alpha | BB324744 | 2.65 | 0.008004 |
| Trichorhinophalangeal syndrome I (human) | BM240648 | 2.02 | 0.002568 |
| Ets variant gene 6 (TEL oncogene) | BB735884 | 4 | 0.007128 |
| Forkhead box P1 | BB472891 | 3.1 | 0.016448 |
| Myocyte enhancer factor 2C | BB558401 | 2.22 | 0.026037 |
| Pre B-cell leukemia transcription factor 1 | AU042527 | 3.02 | 0.000403 |
| Dachshund 1 (Drosophila) | BB374930 | 2.54 | 0.038898 |
| lymphoid enhancer binding factor 1 | AV156352 | 3 | 0.022913 |
| protein phosphatase 1, regulatory (inhibitor) subunit 12C | BE628628 | 2.02 | 0.028272 |
| B-cell CLL/lymphoma 11A (zinc finger protein) | BF731393 | 3.13 | 0.015489 |
| Hypoxia inducible factor 1, alpha subunit | BB409314 | 3.2 | 0.034074 |
| estrogen-related receptor gamma | BM120183 | 5.55 | 0.010962 |
| WW domain containing transcription regulator 1 | AU018221 | 2.12 | 0.045384 |
| Early B-cell factor 1 | BB454472 | 2.51 | 0.01315 |
