# Supplementary material for: Systemic Disease-Induced Salivary Biomarker Profiles in Mouse Models of Melanoma and Non-Small Cell Lung Cancer
Source: PLoS One. 2009 Jun 11;4(6):e5875. doi: 10.1371/journal.pone.0005875 (PMC2691577; doi:10.1371/journal.pone.0005875)
Supplement: Table S2 — (0.04 MB DOC) [file pone.0005875.s002.doc]

**Supplementary Table 2.** Primer pairs of six tranascription factors in salivary gland of melanoma mice model used in qRT-PCR.

| Target gene |  | Primer sequence |
| --- | --- | --- |
| Runx1 | Forward | TTGGTAGGTAACGCAGCAATGTT |
|  | Reverse | CCTTGGAGTGACCCTCAGTGTCT |
| Trim30 | Forward | ACTGATGTTCTATGTGGCACTGAG |
|  | Reverse | CAGCTTCCAGGATCTAATATCCAG |
| Mlxipl | Forward | GCATCCTCATCCGACCTTTATT |
|  | Reverse | GTGGAAGTGCTGAGTTGGCG |
| Egr1 | Forward | TGGATTTTGTTTTCCTTGGGG |
|  | Reverse | TTGGTTTGCTTGGCTTTGCTC |
| Tbx1F | Forward | GGCTCAGACAAAGACGCTGC |
|  | Reverse | GTCCGGAGTCACGGTCGAAC |
| Nr1d1 | Forward | CTGCCACCAACCTAACAGCAAT |
|  | Reverse | GTTGCCTTGCCGTAGACTGTT |
| GAPDH | Forward | ACCACTTTGTCAAGCTCATTTCCT |
|  | Reverse | CACCCTGTTGCTGTAGCCAAAT |
