# Supplementary material for: Systemic Disease-Induced Salivary Biomarker Profiles in Mouse Models of Melanoma and Non-Small Cell Lung Cancer
Source: PLoS One. 2009 Jun 11;4(6):e5875. doi: 10.1371/journal.pone.0005875 (PMC2691577; doi:10.1371/journal.pone.0005875)
Supplement: Table S3 — (0.30 MB DOC) [file pone.0005875.s003.doc]

**Supplementary Table 3**. The list of 225 up-regulated salivary transcripts in melanoma mouse model.

| Probe set | Gene | Fold change | P value |
| --- | --- | --- | --- |
| 1416370_at | zinc finger proliferation 1 | 2.51 | 0.031 |
| 1417341_a_at | protein phosphatase 1, regulatory (inhibitor) subunit 2 | 2.11 | 0.038 |
| 1417554_at | hydroxysteroid dehydrogenase-4, delta<5>-3-beta | 7.6 | 0.034 |
| 1417906_at | RIKEN cDNA 1700001F09 gene | 2.27 | 0.029 |
| 1417958_at | tetraspan 1 | 3.24 | 0.015 |
| 1418075_at | ST6 | 2.71 | 0.027 |
| 1418440_at | procollagen, type VIII, alpha 1 | 6.66 | 0.042 |
| 1418874_a_at | proteasome (prosome, macropain) 26S subunit, non-ATPase, 4 | 3.47 | 0.002 |
| 1419182_at | sushi, von Willebrand factor type A, EGF and pentraxin domain containing 1 | 2.83 | 0.023 |
| 1419227_at | chaperonin subunit 6b (zeta) | 4.09 | 0.009 |
| 1419963_at | DEP domain containing 6 | 2.54 | 0.05 |
| 1419992_x_at | Mm.219839 | 2.79 | 0.008 |
| 1420431_at | repetin | 19.59 | 0.022 |
| 1420536_at | crystallin, beta B2 | 5.07 | 0.008 |
| 1420676_at | small proline rich-like 3 | 18.42 | 0.043 |
| 1420835_at | solute carrier family 25, member 30 | 5.98 | 0.046 |
| 1421079_at | nuclear receptor subfamily 4, group A, member 3 | 2.79 | 0.033 |
| 1421122_at | Casitas B-lineage lymphoma-like 1 | 6.13 | 0.011 |
| 1421248_at | synapsin III | 3.34 | 0.011 |
| 1421381_a_at | procollagen, type IX, alpha 1 | 3.86 | 0.023 |
| 1421816_at | glutathione reductase 1 | 3.04 | 0.016 |
| 1421835_at | microtubule-associated protein 7 | 7.36 | 0.021 |
| 1421940_at | stromal antigen 1 | 3.27 | 0.045 |
| 1422108_at | protein phosphatase 1, regulatory subunit 3A | 3.46 | 0.048 |
| 1422600_at | RAS protein-specific guanine nucleotide-releasing factor 1 | 3.28 | 0.016 |
| 1422826_at | insulin-like growth factor binding protein, acid labile subunit | 2.52 | 0.042 |
| 1422925_s_at | peroxisomal acyl-CoA thioesterase 2A | 6.32 | 0.01 |
| 1422943_a_at | heat shock protein 1 | 2.79 | 0.007 |
| 1422968_at | inositol hexaphosphate kinase 1 | 3.16 | 0.043 |
| 1423049_a_at | tropomyosin 1, alpha | 6.82 | 0.04 |
| 1423406_at | synaptic vesicle glycoprotein 2 a | 2.47 | 0.03 |
| 1423553_at | DnaJ (Hsp40) homolog, subfamily B, member 3 | 5.92 | 0.008 |
| 1423886_at | laminin, gamma 1 | 17.53 | 0.027 |
| 1424212_at | RIKEN cDNA 9430023L20 gene | 3.49 | 0.01 |
| 1424262_at | RIKEN cDNA 2810003C17 gene | 2.5 | 0.018 |
| 1424848_at | potassium large conductance calcium-activated channel,M1 | 3.66 | 0.028 |
| 1424916_x_at | RIKEN cDNA 8030466O12 gene | 3.03 | 0.04 |
| 1425117_at | RIKEN cDNA 0610012D14 gene | 8.21 | 0.034 |
| 1425469_a_at | Mm.1232.1 | 2.79 | 0.019 |
| 1425604_at | v-crk sarcoma virus CT10 oncogene homolog (avian)-like | 3.57 | 0.014 |
| 1425815_a_at | hyaluronan mediated motility receptor (RHAMM) | 3.51 | 0.044 |
| 1425871_a_at | immunoglobulin kappa chain variable 28 (V28) | 5.53 | 0.017 |
| 1425973_at | lysosomal trafficking regulator | 9.58 | 0.008 |
| 1425999_at | complement factor H-related protein | 2.67 | 0.027 |
| 1426032_at | nuclear factor of activated T-cells,calcineurin-dependent 2 | 3.45 | 0.047 |
| 1426164_a_at | upstream transcription factor 1 | 6.71 | 0.044 |
| 1426211_at | cDNA sequence BC021367 | 3.96 | 0.041 |
| 1426975_at | RIKEN cDNA 4632413K17 gene | 22.49 | 0.009 |
| 1427030_at | DNA segment, Chr 16, ERATO Doi 480, expressed | 2.89 | 0.038 |
| 1427078_at | sorting nexin 19 | 14.65 | 0.013 |
| 1427268_at | similar to Acidic ribosomal phosphoprotein P0 | 9.85 | 0.003 |
| 1427551_at | ubiquitin specific protease 29 | 3.2 | 0.016 |
| 1427809_at | RIKEN cDNA A230055J12 gene | 3.18 | 0.006 |
| 1428301_at | similar to hypothetical protein LOC67055 | 3.95 | 0.007 |
| 1430164_a_at | growth factor receptor bound protein 10 | 7.19 | 0.01 |
| 1430708_a_at | ubiquitin specific protease 45 | 4.65 | 0.046 |
| 1433480_at | RIKEN cDNA 2900010J23 gene | 3.21 | 0.009 |
| 1434992_at | RIKEN cDNA 9130206N08 gene | 2.81 | 0.036 |
| 1435372_a_at | proliferation-associated 2G4 | 3.62 | 0.006 |
| 1435527_at | RIKEN cDNA 1500041O16 gene | 2.61 | 0.044 |
| 1435625_at | ectonucleoside triphosphate diphosphohydrolase 7 | 5.38 | 0.041 |
| 1435789_x_at | Regulating synaptic membrane exocytosis 2 | 2.6 | 0.04 |
| 1436665_a_at | latent transforming growth factor beta binding protein 4 | 3.21 | 0.028 |
| 1436784_x_at | splicing factor 3b, subunit 4 | 2.93 | 0.022 |
| 1437991_x_at | RUN and SH3 domain containing 1 | 2.73 | 0.029 |
| 1438095_x_at | Expressed sequence AI326906 | 3.88 | 0.037 |
| 1438723_a_at | ribosomal protein S10 | 3.26 | 0.007 |
| 1439245_at | trinucleotide repeat containing 6a | 3.9 | 0.035 |
| 1447653_x_at | ribosomal protein L24 | 2.83 | 0.044 |
| 1448046_at | Rab9 effector protein with kelch motifs | 3.19 | 0.022 |
| 1448329_at | a disintegrin and metalloprotease domain 3 (cyritestin) | 6.93 | 0.029 |
| 1448674_at | ring finger protein 25 | 4.51 | 0.012 |
| 1448745_s_at | loricrin | 2.51 | 0.023 |
| 1448821_at | tyrosinase | 3.02 | 0.048 |
| 1449250_at | papillary renal cell carcinoma (translocation-associated) | 3.54 | 0.024 |
| 1449276_at | RIKEN cDNA 1700029P11 gene | 3.42 | 0.042 |
| 1449333_at | splicing factor 3a, subunit 1 | 4.89 | 0.042 |
| 1449467_at | spermiogenesis specific transcript on the Y 1 | 8.14 | 0.008 |
| 1449560_at | RIKEN cDNA 2310043L02 gene | 2.79 | 0.048 |
| 1449646_s_at | tigger transposable element derived 5 | 3.75 | 0.023 |
| 1449986_at | RIKEN cDNA 2310034C09 gene | 7.43 | 0.028 |
| 1450041_a_at | tubby candidate gene | 9.61 | 0.019 |
| 1450331_s_at | vomeronasal 2, receptor, 4 | 3.61 | 0.004 |
| 1451355_at | RIKEN cDNA 2410116I05 gene | 3.15 | 0.005 |
| 1451510_s_at | thioesterase domain containing 1 | 5.45 | 0.029 |
| 1451721_a_at | histocompatibility 2, class II antigen A, beta 1 | 2.76 | 0.024 |
| 1451748_a_at | DNA segment, Chr 12, ERATO Doi 771, expressed | 7.52 | 0.045 |
| 1452324_at | plasmacytoma variant translocation 1 | 9.65 | 0.002 |
| 1452636_x_at | GTP binding protein 5 | 3.65 | 0.003 |
| 1452831_s_at | phosphoribosyl pyrophosphate amidotransferase | 2.81 | 0.049 |
| 1452961_at | RIKEN cDNA 1200009O22 gene | 6.56 | 0.019 |
| 1452981_at | contactin 1 | 2.98 | 0.013 |
| 1453571_at | DEP domain containing 6 | 2.73 | 0.018 |
| 1454953_at | ring finger protein 157 | 2.14 | 0.047 |
| 1455061_a_at | acetyl-Coenzyme A acyltransferase 2 | 7.74 | 0.002 |
| 1456584_x_at | similar to 3-phosphoglycerate dehyrogenase | 2.66 | 0.041 |
| 1460252_s_at | zinc finger protein 105 | 3.2 | 0.019 |
| 1460430_at | RAP2C, member of RAS oncogene family | 6.37 | 0.047 |
| 1460639_a_at | ATX1 (antioxidant protein 1) homolog 1 (yeast) | 2.25 | 0.039 |
| 1428174_x_at | KH-type splicing regulatory protein | 2.39 | 0.018 |
| 1429201_at | cylindromatosis (turban tumor syndrome) | 3.16 | 0.017 |
| 1429402_at | glycosyltransferase 8 domain containing 2 | 2.41 | 0.041 |
| 1429772_at | plexin A2 | 5.4 | 0.025 |
| 1429925_at | RIKEN cDNA 4933425B16 gene | 3.65 | 0.032 |
| 1430511_at | RIKEN cDNA 1500037O19 gene | 6.05 | 0.026 |
| 1430731_at | keratin associated protein 2-4 | 4.23 | 0.01 |
| 1431069_at | RIKEN cDNA 4930544G21 gene | 14.73 | 0.039 |
| 1431558_at | RIKEN cDNA 2310016D03 gene | 7.21 | 0.012 |
| 1431952_at | RIKEN cDNA 4930532I03 gene | 4.32 | 0.027 |
| 1432504_at | RIKEN cDNA 1700106N22 gene | 3.34 | 0.006 |
| 1432649_at | RIKEN cDNA 3300002A11 gene | 3.2 | 0.013 |
| 1432669_at | RIKEN cDNA 9030420N05 gene | 2.76 | 0.049 |
| 1432865_at | RIKEN cDNA D730050C22 gene | 3.48 | 0.011 |
| 1432968_at | RIKEN cDNA 4930456A14 gene | 4.5 | 0.019 |
| 1433420_at | RIKEN cDNA 4930527J03 gene | 3.04 | 0.02 |
| 1433583_at | zinc finger protein 365 | 4.16 | 0.004 |
| 1435384_at | ubiquitin-conjugating enzyme E2N | 3.46 | 0.045 |
| 1435851_at | leucine-rich repeat LGI family, member 1 | 17.52 | 0.038 |
| 1436173_at | Deleted in liver cancer 1 | 5.3 | 0.045 |
| 1436251_at | phosphodiesterase 1C | 3.77 | 0.033 |
| 1436264_at | cDNA sequence BC025920 | 5.91 | 0.037 |
| 1436578_at | RIKEN cDNA A330104H05 gene | 3.23 | 0.027 |
| 1436624_at | dynamin 3 | 2.98 | 0.02 |
| 1437068_at | SoxLZ/Sox6 leucine zipper binding protein in testis | 7.65 | 0.019 |
| 1437412_at | peroxiredoxin 6, related sequence 1 | 6.57 | 0.001 |
| 1437431_at | RIKEN cDNA 2600010L24 gene | 5.05 | 0.027 |
| 1437471_at | leucine rich repeat containing 45 | 3.03 | 0.045 |
| 1437792_at | RIKEN cDNA 1700013D24 gene | 8.38 | 0.043 |
| 1438128_at | Adenosine monophosphate deaminase 2 (isoform L) | 6.02 | 0.045 |
| 1438615_x_at | RIKEN cDNA 2010317E24 gene | 3.95 | 0.048 |
| 1439412_at | RIKEN cDNA 0710001E13 gene | 3.17 | 0.01 |
| 1439467_at | Microtubule-associated protein 4 | 3.41 | 0.028 |
| 1439588_at | solute carrier organic anion transporter family, member 5A1 | 5.97 | 0.045 |
| 1440228_at | RAN binding protein 6 | 5.39 | 0.04 |
| 1440508_at | Mm.187120 | 4.75 | 0.002 |
| 1440692_at | gene model 364, (NCBI) | 6.66 | 0.016 |
| 1440779_s_at | solute carrier family 5, member 9 | 2.85 | 0.019 |
| 1440791_x_at | transcription elongation factor A (SII), 2 | 3.68 | 0.043 |
| 1440845_at | RIKEN cDNA E030031F02 gene | 7.64 | 0.002 |
| 1441004_at | Exosome component 4 | 10.6 | 0.021 |
| 1441049_at | potassium voltage-gated channel | 6.16 | 0.009 |
| 1441255_at | Cadherin 3 | 5.76 | 0.041 |
| 1441737_s_at | Ras association (RalGDS/AF-6) domain family 1 | 2.85 | 0.024 |
| 1441873_at | prolactin-like protein E | 3.14 | 0.021 |
| 1441929_at | Mm.155698 | 4.72 | 0.029 |
| 1441947_x_at | cDNA sequence BC033915 | 3.58 | 0.008 |
| 1442082_at | complement component 3a receptor 1 | 2.87 | 0.016 |
| 1442101_at | RIKEN cDNA A930017N06 gene | 3.11 | 0.025 |
| 1442274_at | zinc finger, DHHC domain containing 15 | 3.29 | 0.034 |
| 1442859_at | Sprouty protein with EVH-1 domain 1, related sequence | 5.38 | 0.014 |
| 1442891_at | expressed sequence AU023386 | 12.42 | 0.022 |
| 1443142_at | Beta-transducin repeat containing protein | 3.25 | 0.007 |
| 1443257_at | RIKEN cDNA 9630050E16 gene | 10.29 | 0.031 |
| 1443724_at | junctophilin 3 | 2.64 | 0.039 |
| 1443798_at | Mm.131916 | 2.44 | 0.031 |
| 1443824_s_at | carbonic anhydrase 7 | 3.13 | 0.023 |
| 1443835_x_at | RIKEN cDNA 1110014K08 gene | 3.46 | 0.017 |
| 1443984_at | lin-9 homolog (C. elegans) | 2.79 | 0.029 |
| 1444025_at | Mm.209030 | 7.55 | 0.038 |
| 1444156_at | RIKEN cDNA 9230112E08 gene | 33.73 | 0.001 |
| 1444192_at | 12 days embryo spinal ganglion cDNA | 3.19 | 0.046 |
| 1444199_at | Mm.45087.1 | 3.26 | 0.02 |
| 1444222_x_at | Zinc finger protein 580 | 3.84 | 0.008 |
| 1444432_at | RIKEN cDNA D330040H18 gene | 5.2 | 0.045 |
| 1444519_at | leucine rich repeat containing G protein coupled receptor 5 | 4.23 | 0.014 |
| 1444724_at | Transcribed locus | 2.53 | 0.03 |
| 1444995_at | Mm.210114 | 8.23 | 0.014 |
| 1445230_at | RIKEN cDNA 5730405M06 gene | 4.51 | 0.029 |
| 1445284_at | Mm.26155 | 2.57 | 0.016 |
| 1445323_at | gene regulated by estrogen in breast cancer protein | 4.63 | 0.028 |
| 1445326_at | sidekick homolog 1 (chicken) | 20.12 | 0.037 |
| 1445502_at | 15 days embryo head cDNA, RIKEN full-length enriched library | 2.92 | 0.028 |
| 1445718_at | WD repeat and FYVE domain containing 3 | 2.57 | 0.021 |
| 1445835_at | RIKEN cDNA C530030K21 gene | 5.17 | 0.028 |
| 1445935_at | Mm.26241 | 2.83 | 0.042 |
| 1446003_at | Mm.173527 | 3.1 | 0.007 |
| 1446067_at | Piwi like homolog 2 (Drosophila) | 4.09 | 0.006 |
| 1446194_at | nuclear receptor subfamily 2, group C, member 1 | 3.2 | 0.025 |
| 1446328_at | Transcribed locus | 4.13 | 0.021 |
| 1446883_at | RIKEN cDNA 4933427D06 gene | 4.6 | 0.032 |
| 1447070_at | Williams-Beuren syndrome chromosome region 1 homolog (human) | 4.18 | 0.03 |
| 1447381_at | Mm.218119 | 3.52 | 0.008 |
| 1447518_at | TPX2, microtubule-associated protein homolog (Xenopus laevis) | 3.57 | 0.037 |
| 1447519_x_at | TPX2, microtubule-associated protein homolog (Xenopus laevis) | 3.12 | 0.034 |
| 1447621_s_at | RIKEN cDNA 2610307O08 gene | 6.06 | 0.03 |
| 1447625_at | E2F transcription factor 5 | 3.26 | 0.012 |
| 1447641_at | dystrophia myotonica-containing WD repeat motif | 4.35 | 0.026 |
| 1447642_x_at | dystrophia myotonica-containing WD repeat motif | 3.92 | 0.014 |
| 1447666_x_at | Mm.180236 | 4.09 | 0.016 |
| 1447672_x_at | protein serine kinase H1 | 2.61 | 0.023 |
| 1447717_x_at | gene model 123, (NCBI) | 2.94 | 0.026 |
| 1447742_at | lysosomal-associated protein transmembrane 5 | 3.78 | 0.005 |
| 1447778_x_at | c6.1a protein | 2.84 | 0.047 |
| 1447908_x_at | tetratricopeptide repeat domain 3 | 3.3 | 0.014 |
| 1447946_at | expressed sequence AW046396 | 3.68 | 0.022 |
| 1447980_s_at | RUN and TBC1 domain containing 2 | 3.88 | 0.042 |
| 1452690_at | KH-type splicing regulatory protein | 3.55 | 0.008 |
| 1453254_at | RIKEN cDNA 1700034O15 gene | 3.08 | 0.015 |
| 1453781_at | Mm.205165.1 | 3.14 | 0.018 |
| 1453883_at | RIKEN cDNA 4933425B07 gene | 3.38 | 0.026 |
| 1454099_at | RIKEN cDNA 4930593A02 gene | 3.55 | 0.009 |
| 1454444_at | RIKEN cDNA 5730406E14 gene | 3.53 | 0.005 |
| 1454555_at | RIKEN cDNA 4930472F24 gene | 3.25 | 0.023 |
| 1454829_at | RUN domain containing 1 | 2.46 | 0.035 |
| 1454844_at | Transcribed locus | 3.58 | 0.007 |
| 1455872_at | hypothetical protein A030013D21 | 5.41 | 0.043 |
| 1456166_at | Transcribed locus | 5.59 | 0.015 |
| 1456674_at | RIKEN cDNA E130016E03 gene | 3.53 | 0.032 |
| 1457009_at | Rho-related BTB domain containing 3 | 3.17 | 0.038 |
| 1457127_at | Adult male epididymis cDNA, RIKEN full-length enriched library | 3.17 | 0.031 |
| 1457284_at | CDNA clone IMAGE:5256843, partial cds | 4.29 | 0.039 |
| 1457288_at | ankyrin 3, epithelial | 5.31 | 0.019 |
| 1457648_x_at | cDNA sequence BC004044 | 3.28 | 0.009 |
| 1457960_at | Mitogen-activated protein kinase kinase kinase 9 | 4.75 | 0.041 |
| 1458274_at | zinc finger protein 69 | 5.33 | 0.025 |
| 1458739_at | Latent transforming growth factor beta binding protein 1 | 4.41 | 0.046 |
| 1458764_at | Mm.218055 | 2.7 | 0.021 |
| 1459062_x_at | Hypothetical protein A130033P14 | 3.9 | 0.011 |
| 1459142_at | RIKEN cDNA 4931427F14 gene | 8.24 | 0.018 |
| 1459299_at | RIKEN cDNA A430065P19 gene | 5.6 | 0.04 |
| 1459516_at | RIKEN cDNA A930035D04 gene | 3.42 | 0.035 |
| 1459642_at | Expressed sequence AW046396 | 4.61 | 0.048 |
| 1459877_x_at | Ring finger protein 185 | 3.96 | 0.031 |
| 1460119_at | Mm.133656 | 2.89 | 0.027 |
| 1460488_at | zinc finger and BTB domain containing 4 | 2.45 | 0.028 |
