# Supplementary material for: Systemic Disease-Induced Salivary Biomarker Profiles in Mouse Models of Melanoma and Non-Small Cell Lung Cancer
Source: PLoS One. 2009 Jun 11;4(6):e5875. doi: 10.1371/journal.pone.0005875 (PMC2691577; doi:10.1371/journal.pone.0005875)
Supplement: Table S4 — (0.51 MB DOC) [file pone.0005875.s004.doc]

**Supplementary Table 4**. The list of 403 down-regulated salivary transcripts in melanoma mouse model.

| probe set | gene | fold change | P value |
| --- | --- | --- | --- |
| 1415722_a_at | RIKEN cDNA 1110059P08 gene | -6.74 | 0.022 |
| 1415751_at | heterochromatin protein 1, binding protein 3 | -5.39 | 0.038 |
| 1415763_a_at | Mm.35837 | -36.02 | 0.032 |
| 1415781_a_at | SMT3 suppressor of mif two 3 homolog 2 (yeast) | -53.25 | 0.039 |
| 1415928_a_at | microtubule-associated protein 1 light chain 3 beta | -138.42 | 0.026 |
| 1415930_a_at | similar to microtubule-associated protein 1 light chain 3 beta | -14.47 | 0.037 |
| 1415966_a_at | NADH dehydrogenase (ubiquinone) flavoprotein 1 | -39.28 | 0.041 |
| 1416001_a_at | coactosin-like 1 (Dictyostelium) | -9.79 | 0.046 |
| 1416021_a_at | fatty acid binding protein 5, epidermal | -72.38 | 0.034 |
| 1416024_x_at | chaperonin subunit 3 (gamma) | -85.49 | 0.036 |
| 1416057_at | NADH dehydrogenase (ubiquinone) 1 beta subcomplex, 11 | -99.71 | 0.039 |
| 1416099_at | ribosomal protein L27 | -7.34 | 0.04 |
| 1416119_at | thioredoxin 1 | -8.58 | 0.011 |
| 1416143_at | ATP synthase, H+ transporting, mitochondrial F0 complex, subunit F | -197 | 0.038 |
| 1416233_at | eukaryotic translation initiation factor 3, subunit 2 (beta) | -99.17 | 0.035 |
| 1416248_at | NAD kinase | -6.77 | 0.031 |
| 1416303_at | LPS-induced TN factor | -48.58 | 0.027 |
| 1416381_a_at | peroxiredoxin 5 | -208.03 | 0.009 |
| 1416480_a_at | hypoxia induced gene 1 | -49.34 | 0.037 |
| 1416499_a_at | dynactin 6 | -68.91 | 0.038 |
| 1416533_at | EGL nine homolog 2 (C. elegans) | -3.86 | 0.042 |
| 1416582_a_at | Bcl-associated death promoter | -9.74 | 0.039 |
| 1416603_at | ribosomal protein L22 | -162.41 | 0.026 |
| 1416604_at | cytochrome c-1 | -25.23 | 0.025 |
| 1416642_a_at | Tpt1 pseudogene | -209.29 | 0.02 |
| 1416667_at | phenylalkylamine Ca2+ antagonist (emopamil) binding protein | -23.7 | 0.044 |
| 1416788_a_at | isocitrate dehydrogenase 3 (NAD+), gamma | -12.82 | 0.036 |
| 1416923_a_at | BCL2/adenovirus E1B 19kDa-interacting protein 3-like | -63.91 | 0.021 |
| 1416937_at | gamma-aminobutyric acid receptor associated protein | -13.44 | 0.036 |
| 1416948_at | mitochondrial ribosomal protein L23 | -27.77 | 0.021 |
| 1416970_a_at | cytochrome c oxidase, subunit VIIa 2 | -9.08 | 0.031 |
| 1416971_at | cytochrome c oxidase, subunit VIIa 2 | -20.53 | 0.029 |
| 1417060_at | protein phosphatase 1, regulatory (inhibitor) subunit 11 | -3.82 | 0.048 |
| 1417065_at | early growth response 1 | -15.54 | 0.018 |
| 1417081_a_at | synaptogyrin 2 | -4.52 | 0.048 |
| 1417102_a_at | NADH dehydrogenase (ubiquinone) 1 beta subcomplex, 5 | -22.88 | 0.041 |
| 1417204_at | KDEL (Lys-Asp-Glu-Leu) endoplasmic reticulum protein retention receptor 2 | -8.97 | 0.045 |
| 1417260_at | U2 small nuclear ribonucleoprotein auxiliary factor (U2AF) 2 | -7.84 | 0.041 |
| 1417274_at | small nuclear ribonucleoprotein polypeptide A | -16.75 | 0.035 |
| 1417285_a_at | NADH dehydrogenase (ubiquinone) 1 alpha subcomplex, 5 | -83.76 | 0.037 |
| 1417348_at | RIKEN cDNA 2310039H08 gene | -33.97 | 0.037 |
| 1417357_at | emerin | -164.53 | 0.001 |
| 1417364_at | eukaryotic translation elongation factor 1 gamma | -12.42 | 0.046 |
| 1417370_at | trefoil factor 3, intestinal | -2.81 | 0.015 |
| 1417463_a_at | RIKEN cDNA 2400001E08 gene | -23.27 | 0.025 |
| 1417560_at | sideroflexin 1 | -25.42 | 0.044 |
| 1417562_at | eukaryotic translation initiation factor 4E binding protein 1 | -10.09 | 0.047 |
| 1417615_a_at | ribosomal protein L11 | -110.69 | 0.032 |
| 1417964_at | adaptor-related protein complex 3, delta 1 subunit | -36.94 | 0.027 |
| 1418002_at | RIKEN cDNA 2010110M21 gene | -131.83 | 0.048 |
| 1418101_a_at | reticulon 3 | -22.36 | 0.028 |
| 1418234_s_at | breast carcinoma amplified sequence 2 | -17.03 | 0.045 |
| 1418436_at | syntaxin 7 | -4.51 | 0.037 |
| 1418574_a_at | split hand/foot deleted gene 1 | -28.92 | 0.049 |
| 1418814_s_at | NADH dehydrogenase (ubiquinone) 1 alpha subcomplex, 12 | -21.05 | 0.037 |
| 1418822_a_at | ADP-ribosylation factor 6 | -9.25 | 0.015 |
| 1418883_a_at | poly A binding protein, cytoplasmic 1 | -29.54 | 0.023 |
| 1418885_a_at | isocitrate dehydrogenase 3 (NAD+) beta | -6.44 | 0.023 |
| 1419027_s_at | glycolipid transfer protein | -5.34 | 0.02 |
| 1419177_at | DNA segment, Chr 8, ERATO Doi 531, expressed | -7.1 | 0.036 |
| 1419194_s_at | glia maturation factor, gamma | -31.42 | 0.016 |
| 1419350_at | hook homolog 2 (Drosophila) | -40.21 | 0.03 |
| 1419363_a_at | mitochondrial ribosomal protein L35 | -29.21 | 0.031 |
| 1419364_a_at | ribosomal protein S7 | -136.09 | 0.033 |
| 1419387_s_at | mucin 13, epithelial transmembrane | -17.14 | 0.029 |
| 1419509_a_at | N-acetylglucosamine kinase | -22.71 | 0.05 |
| 1419536_a_at | v-rel reticuloendotheliosis viral oncogene homolog A (avian) | -84.46 | 0.049 |
| 1419569_a_at | interferon-stimulated protein | -94.2 | 0.011 |
| 1419803_s_at | coiled-coil domain containing 12 | -144.14 | 0.027 |
| 1420088_at | Mm.203821 | -33.23 | 0.027 |
| 1420129_s_at | DNA segment, Chr 10, Wayne State University 52, expressed | -360.37 | 0.026 |
| 1420197_at | Growth arrest and DNA-damage-inducible 45 beta | -2.84 | 0.028 |
| 1420352_at | protease, serine, 22 | -7.26 | 0.029 |
| 1420394_s_at | glycoprotein 49 A | -43.82 | 0.024 |
| 1420567_at | protein kinase C, nu | -8.71 | 0.025 |
| 1420699_at | C-type lectin domain family 7, member a | -2.9 | 0.034 |
| 1420820_at | RIKEN cDNA 2900073G15 gene | -53.56 | 0.037 |
| 1420827_a_at | cyclin G1 | -8.35 | 0.011 |
| 1420952_at | Son cell proliferation protein | -39.42 | 0.034 |
| 1421053_at | kinesin family member 1A | -4.17 | 0.046 |
| 1421776_at | olfactory receptor 74 | -5.22 | 0.02 |
| 1421911_at | signal transducer and activator of transcription 2 | -7.73 | 0.037 |
| 1422032_a_at | zinc finger, A20 domain containing 3 | -6.65 | 0.038 |
| 1422241_a_at | NADH dehydrogenase (ubiquinone) 1 alpha subcomplex, 1 | -118.42 | 0.013 |
| 1422506_a_at | cystatin B | -40.96 | 0.046 |
| 1422848_a_at | poly(A) binding protein, nuclear 1 | -13.55 | 0.025 |
| 1422849_a_at | poly(A) binding protein, nuclear 1 | -7.57 | 0.027 |
| 1423079_a_at | translocase of outer mitochondrial membrane 20 homolog (yeast) | -17.32 | 0.027 |
| 1423090_x_at | SEC61, gamma subunit | -51.38 | 0.028 |
| 1423223_a_at | peroxiredoxin 6 | -4.2 | 0.044 |
| 1423254_x_at | ribosomal protein S27-like | -57.78 | 0.026 |
| 1423376_a_at | docking protein 4 | -6.45 | 0.041 |
| 1423455_at | prothymosin alpha | -12.46 | 0.042 |
| 1423686_a_at | RIKEN cDNA 1110020C13 gene | -32.64 | 0.037 |
| 1423687_a_at | mannosidase, alpha, class 2C, member 1 | -85.79 | 0.018 |
| 1423734_at | RAS-related C3 botulinum substrate 1 | -42.82 | 0.036 |
| 1423798_a_at | suppressor of initiator codon mutations, related sequence 1 | -18.85 | 0.049 |
| 1423807_a_at | calmodulin 2 | -40.29 | 0.04 |
| 1423839_a_at | basic transcription factor 3 | -100.67 | 0.01 |
| 1423985_at | guanine nucleotide binding protein (G protein), gamma 5 subunit | -11.53 | 0.019 |
| 1424000_a_at | ribosomal protein S11 | -94.87 | 0.05 |
| 1424036_at | RIKEN cDNA 2610031L17 gene | -45.43 | 0.047 |
| 1424254_at | interferon induced transmembrane protein 1 | -74.01 | 0.04 |
| 1424380_at | cDNA sequence BC026744 | -2.82 | 0.038 |
| 1424384_a_at | zinc and ring finger 1 | -23.98 | 0.03 |
| 1424489_a_at | tRNA isopentenyltransferase 1 | -15.32 | 0.048 |
| 1425113_x_at | Mus musculus LOC433960 (LOC433960), mRNA | -5.43 | 0.032 |
| 1425412_at | cold autoinflammatory syndrome 1 homolog (human) | -13.18 | 0.038 |
| 1425419_a_at | v-raf-1 leukemia viral oncogene 1 | -38.24 | 0.021 |
| 1425444_a_at | transforming growth factor, beta receptor II | -5.91 | 0.041 |
| 1425548_a_at | leukocyte specific transcript 1 | -18.5 | 0.027 |
| 1425567_a_at | annexin A5 | -61.84 | 0.022 |
| 1425685_at | RIKEN cDNA 2310005E10 gene | -5.36 | 0.045 |
| 1425742_a_at | transforming growth factor beta 1 induced transcript 4 | -30.74 | 0.037 |
| 1425884_at | brix domain containing 1 | -19.67 | 0.036 |
| 1425904_at | special AT-rich sequence binding protein 2 | -13.79 | 0.002 |
| 1426095_a_at | tumor necrosis factor receptor superfamily, member 22 | -5.44 | 0.048 |
| 1426236_a_at | glutamate-ammonia ligase (glutamine synthase) | -21.24 | 0.039 |
| 1426659_a_at | ribosomal protein L23a | -53.3 | 0.044 |
| 1427409_at | membrane-associated ring finger (C3HC4) 9 | -7.82 | 0.024 |
| 1427481_a_at | ATPase, Na+/K+ transporting, alpha 3 polypeptide | -12.29 | 0.036 |
| 1427683_at | early growth response 2 | -9.73 | 0.006 |
| 1427929_a_at | pyridoxal (pyridoxine, vitamin B6) kinase | -44.43 | 0.038 |
| 1427955_a_at | differentially expressed in B16F10 1 | -51.6 | 0.021 |
| 1428161_a_at | coiled-coil-helix-coiled-coil-helix domain containing 2 | -6.48 | 0.029 |
| 1428209_at | brain expressed, X-linked 4 | -3.88 | 0.022 |
| 1428212_x_at | ribosomal protein L31 | -124.94 | 0.031 |
| 1428360_x_at | NADH dehydrogenase (ubiquinone) 1 alpha subcomplex, 7 (B14.5a) | -10.87 | 0.046 |
| 1429265_a_at | ring finger protein 130 | -50.33 | 0.02 |
| 1429554_at | serologically defined colon cancer antigen 3 | -15.43 | 0.015 |
| 1430713_s_at | NADH dehydrogenase (ubiquinone) 1 alpha subcomplex, 13 | -12.24 | 0.041 |
| 1431182_at | heat shock protein 8 | -6.44 | 1E-04 |
| 1431765_a_at | ribosomal protein S2 | -30.06 | 0.048 |
| 1431766_x_at | ribosomal protein S2 | -4.62 | 0.05 |
| 1432263_a_at | cytochrome c oxidase subunit VIIa polypeptide 2-like | -187.87 | 0.022 |
| 1432827_x_at | ubiquitin C | -78.85 | 0.048 |
| 1433428_x_at | transglutaminase 2, C polypeptide | -4.54 | 0.034 |
| 1433513_x_at | NADH dehydrogenase (ubiquinone) 1 alpha subcomplex, 12 | -44.43 | 0.033 |
| 1433550_at | checkpoint with forkhead and ring finger domains | -19.34 | 0.004 |
| 1433569_x_at | RAN, member RAS oncogene family | -95.82 | 0.028 |
| 1433688_x_at | ribosomal protein L14 | -17.27 | 0.038 |
| 1433720_s_at | Nur77 downstream gene 2 | -59.16 | 0.029 |
| 1433908_a_at | cortactin | -539.49 | 0.02 |
| 1433966_x_at | asparagine synthetase | -138.25 | 0.049 |
| 1434078_at | DNA segment, Chr 7, Wayne State University 128, expressed | -4.72 | 0.048 |
| 1434127_a_at | H3 histone, family 3A | -22.71 | 0.024 |
| 1434145_s_at | serine hydrolase-like | -110.32 | 0.005 |
| 1434198_at | ATPase inhibitory factor 1 | -11.89 | 0.034 |
| 1434231_x_at | ribosomal protein L35 | -7 | 0.035 |
| 1434334_at | protein kinase D2 | -4.29 | 0.011 |
| 1434378_a_at | Max dimerization protein 4 | -32.84 | 0.016 |
| 1434396_a_at | myosin, light polypeptide 6, alkali, smooth muscle and non-muscle | -55.78 | 0.017 |
| 1434420_x_at | translocase of outer mitochondrial membrane 22 homolog (yeast) | -67.79 | 0.049 |
| 1434637_x_at | transcriptional regulator, SIN3B (yeast) | -7.7 | 0.014 |
| 1434731_x_at | peroxiredoxin 1 | -38.66 | 0.017 |
| 1434971_x_at | Mm.29904 | -7.85 | 0.039 |
| 1434976_x_at | eukaryotic translation initiation factor 4E binding protein 1 | -28.27 | 0.038 |
| 1435151_a_at | ribosomal protein S3 | -62.97 | 0.033 |
| 1435277_x_at | expressed in non-metastatic cells 1, protein | -39.45 | 0.036 |
| 1435413_x_at | RIKEN cDNA 2700060E02 gene | -193.79 | 0.041 |
| 1435429_x_at | ribosomal protein S27-like | -34.47 | 0.024 |
| 1435458_at | Mm.2322 | -6.34 | 0.013 |
| 1435712_a_at | ribosomal protein S18 | -173.87 | 0.013 |
| 1435732_x_at | ATPase, H+ transporting, V0 subunit C | -50.63 | 0.033 |
| 1435738_x_at | small EDRK-rich factor 2 | -13.83 | 0.031 |
| 1435757_a_at | ubiquinol cytochrome c reductase core protein 2 | -10.36 | 0.04 |
| 1435817_x_at | similar to 40S ribosomal protein S6 | -25.56 | 0.008 |
| 1436292_a_at | ornithine decarboxylase antizyme | -4.08 | 0.045 |
| 1436688_x_at | ribosomal protein L14 | -15.64 | 0.049 |
| 1436746_at | protein kinase, lysine deficient 1 | -17.91 | 0.046 |
| 1436783_x_at | tyrosine 3-monooxygenase activation protein, beta polypeptide | -151.95 | 0.017 |
| 1436803_a_at | NADH dehydrogenase (ubiquinone) 1 beta subcomplex, 9 | -52.6 | 0.047 |
| 1436840_x_at | ribosomal protein L35 | -7 | 0.045 |
| 1436923_at | RAB2B, member RAS oncogene family | -3.21 | 0.041 |
| 1436924_x_at | ribosomal protein L31 | -248.03 | 0.034 |
| 1436934_s_at | aconitase 2, mitochondrial | -98.16 | 0.021 |
| 1436946_s_at | guanine nucleotide binding protein (G protein), gamma 5 subunit | -3.35 | 0.049 |
| 1436949_a_at | transcription elongation factor B (SIII), polypeptide 2 | -94.79 | 0.043 |
| 1436991_x_at | gelsolin | -69.47 | 0.04 |
| 1436992_x_at | Mm.3555 | -3.79 | 0.015 |
| 1436994_a_at | histone 1, H1c | -59.34 | 0.03 |
| 1436995_a_at | ribosomal protein L26 | -119.18 | 0.037 |
| 1437005_a_at | ribosomal protein L18 | -57.84 | 0.046 |
| 1437131_x_at | mitochondrial ribosomal protein L11 | -80.95 | 0.024 |
| 1437133_x_at | aldo-keto reductase family 1, member B3 (aldose reductase) | -74.01 | 0.021 |
| 1437185_s_at | thymosin, beta 10 | -66.73 | 0.036 |
| 1437192_x_at | voltage-dependent anion channel 1 | -388.97 | 0.018 |
| 1437246_x_at | ribosomal protein S6 | -34.5 | 0.021 |
| 1437277_x_at | transglutaminase 2, C polypeptide | -10.14 | 0.04 |
| 1437280_s_at | RIKEN cDNA 1200009K13 gene | -6.17 | 0.014 |
| 1437455_a_at | B-cell translocation gene 1, anti-proliferative | -38.73 | 0.026 |
| 1437610_x_at | ribosomal protein S8 | -82.07 | 0.035 |
| 1437621_x_at | 3-phosphoglycerate dehydrogenase | -7.73 | 0.038 |
| 1437708_x_at | vesicle-associated membrane protein 3 | -4.42 | 0.014 |
| 1437839_x_at | mitochondrial ribosomal protein L11 | -40.43 | 0.03 |
| 1437947_x_at | Mm.3555 | -1301.94 | 0.027 |
| 1437993_x_at | quininoid dihydropteridine reductase | -3.54 | 0.033 |
| 1438115_a_at | solute carrier family 9 (sodium/hydrogen exchanger), isoform 3 regulator 1 | -26.19 | 0.029 |
| 1438154_x_at | RIKEN cDNA 2610002J02 gene | -101.76 | 0.011 |
| 1438155_x_at | phosphatidylinositol glycan, class O | -35.05 | 0.036 |
| 1438174_x_at | protein phosphatase 2 (formerly 2A), regulatory subunit A (PR 65), alpha isoform | -27.13 | 0.03 |
| 1438211_s_at | D site albumin promoter binding protein | -180.92 | 0.046 |
| 1438315_x_at | aldo-keto reductase family 7, member A5 (aflatoxin aldehyde reductase) | -33.43 | 0.048 |
| 1438360_x_at | solute carrier family 25, member 5 | -231.52 | 0.018 |
| 1438383_x_at | protein phosphatase 2 (formerly 2A), regulatory subunit A (PR 65), alpha isoform | -51.53 | 0.031 |
| 1438390_s_at | pituitary tumor-transforming 1 | -22.83 | 0.028 |
| 1438634_x_at | LIM and SH3 protein 1 | -22.24 | 0.011 |
| 1438646_x_at | RIKEN cDNA 2510039O18 gene | -102.86 | 0.02 |
| 1438653_x_at | spinocerebellar ataxia 10 homolog (human) | -15.44 | 0.007 |
| 1438708_x_at | tryptophan 5-monooxygenase activation protein, beta polypeptide | -204.48 | 0.016 |
| 1438794_x_at | Ribosomal protein S13 | -5.87 | 0.037 |
| 1438847_at | Max dimerization protein 3 | -30.12 | 0.001 |
| 1438853_x_at | DEAD (Asp-Glu-Ala-Asp) box polypeptide 54 | -46.04 | 0.029 |
| 1438922_x_at | solute carrier family 25, member 5 | -546.01 | 0.02 |
| 1438925_x_at | ATPase, H+ transporting, V0 subunit C | -12.21 | 0.015 |
| 1438940_x_at | high mobility group nucleosomal binding domain 1 | -338.82 | 0.038 |
| 1438984_x_at | proteasome (prosome, macropain) subunit, beta type 4 | -83.17 | 0.018 |
| 1438991_x_at | protein phosphatase 2 (formerly 2A), regulatory subunit A (PR 65), alpha isoform | -33.78 | 0.035 |
| 1438992_x_at | activating transcription factor 4 | -26.1 | 0.017 |
| 1439184_s_at | thioredoxin-like 5 | -119.55 | 0.02 |
| 1439253_x_at | RIKEN cDNA 2610524G07 gene | -54.99 | 0.031 |
| 1439270_x_at | RAN, member RAS oncogene family | -93 | 0.038 |
| 1439375_x_at | Aldolase 1, A isoform | -12.28 | 0.038 |
| 1439410_x_at | DNA segment, Chr 11, ERATO Doi 333, expressed | -7.36 | 0.043 |
| 1439435_x_at | phosphoglycerate kinase 1 | -12.44 | 0.043 |
| 1439466_s_at | expressed sequence C77604 | -68.53 | 0.036 |
| 1440323_at | synaptotagmin II | -5.56 | 0.012 |
| 1444952_a_at | nuclear casein kinase and cyclin-dependent kinase substrate 1 | -123.15 | 0.024 |
| 1447956_at | expressed sequence C76614 | -42.77 | 0.012 |
| 1448100_at | RIKEN cDNA 4833439L19 gene | -25.09 | 0.039 |
| 1448109_a_at | ribosomal protein L26 | -269.42 | 0.038 |
| 1448153_at | cytochrome c oxidase, subunit Va | -76.83 | 0.018 |
| 1448179_at | upregulated during skeletal muscle growth 5 | -118.77 | 0.016 |
| 1448202_x_at | RIKEN cDNA 2610524G07 gene | -39.36 | 0.032 |
| 1448217_a_at | ribosomal protein L27 | -49.9 | 0.036 |
| 1448232_x_at | tubulin, alpha 6 | -17.35 | 0.024 |
| 1448252_a_at | eukaryotic translation elongation factor 1 beta 2 | -26.92 | 0.011 |
| 1448264_a_at | eukaryotic translation initiation factor 3, subunit 2 (beta) | -169.15 | 0.015 |
| 1448284_a_at | NADH dehydrogenase (ubiquinone) 1, subcomplex unknown, 1 | -131.44 | 0.013 |
| 1448297_a_at | tyrosine kinase, non-receptor, 2 | -4.56 | 0.005 |
| 1448334_a_at | cyclin I | -24.52 | 0.041 |
| 1448418_s_at | WD repeat domain 23 | -80.76 | 0.015 |
| 1448503_at | myeloid cell leukemia sequence 1 | -9.51 | 0.011 |
| 1448533_at | cytoskeleton-associated protein 1 | -33.8 | 0.014 |
| 1448540_a_at | RIKEN cDNA 0610012G03 gene | -10.94 | 0.03 |
| 1448568_a_at | solute carrier family 20, member 1 | -4.48 | 0.018 |
| 1448697_s_at | ribosomal protein L36a-like | -77.55 | 0.023 |
| 1448739_x_at | ribosomal protein S18 | -127.16 | 0.046 |
| 1448770_a_at | ATPase inhibitory factor 1 | -15.64 | 0.025 |
| 1448771_a_at | ferritin heavy chain 1 | -5.28 | 0.034 |
| 1448812_at | hippocalcin-like 1 | -15.25 | 0.003 |
| 1448830_at | dual specificity phosphatase 1 | -45.44 | 0.014 |
| 1448866_at | SUMO/sentrin specific protease 3 | -6.42 | 0.037 |
| 1448881_at | haptoglobin | -51.18 | 0.04 |
| 1448887_x_at | fractured callus expressed transcript 1 | -178.53 | 0.044 |
| 1448934_at | NADH dehydrogenase (ubiquinone) 1 alpha subcomplex 10 | -5.95 | 0.038 |
| 1449040_a_at | selenophosphate synthetase 2 | -256.74 | 0.013 |
| 1449108_at | ferredoxin 1 | -93.24 | 0.039 |
| 1449127_at | selectin, platelet (p-selectin) ligand | -13.47 | 0.048 |
| 1449243_a_at | ribosomal protein S19 | -665.64 | 0.031 |
| 1449255_a_at | ribosomal protein L15 | -5.91 | 0.043 |
| 1449399_a_at | interleukin 1 beta | -50.03 | 0.026 |
| 1449412_at | RIKEN cDNA 1810046J19 gene | -330.25 | 0.025 |
| 1449447_at | cystatin 10 (chondrocytes) | -48.31 | 0.033 |
| 1449552_at | zinc finger RNA binding protein | -5.43 | 0.037 |
| 1449628_s_at | START domain containing 7 | -18.4 | 0.048 |
| 1449677_s_at | transmembrane protein 38B | -17.96 | 0.019 |
| 1449838_at | cysteine-rich secretory protein 3 | -32.79 | 0.032 |
| 1449875_s_at | histocompatibility 2, T region locus 10 | -60.72 | 0.037 |
| 1449984_at | chemokine (C-X-C motif) ligand 2 | -54.28 | 0.019 |
| 1450009_at | lactotransferrin | -67.98 | 0.039 |
| 1450054_at | adducin 1 (alpha) | -114.67 | 0.046 |
| 1450103_a_at | pleckstrin homology, Sec7 and coiled-coil domains 2 | -2.95 | 0.042 |
| 1450138_a_at | serine (or cysteine) proteinase inhibitor, clade B, member 6a | -66.39 | 0.039 |
| 1450668_s_at | heat shock protein 1 (chaperonin 10) | -17.35 | 0.036 |
| 1450711_at | bromodomain containing 4 | -126.38 | 0.036 |
| 1450746_at | kelch-like ECH-associated protein 1 | -294.09 | 0.043 |
| 1450840_a_at | ribosomal protein L39 | -187.07 | 0.023 |
| 1450925_a_at | ribosomal protein S27-like | -24.49 | 0.022 |
| 1451068_s_at | ribosomal protein S25 | -67.9 | 0.023 |
| 1451172_at | RIKEN cDNA 1200015A19 gene | -170.01 | 0.039 |
| 1451205_at | proteasome (prosome, macropain) subunit, beta type 4 | -42.31 | 0.043 |
| 1451294_s_at | small nuclear ribonucleoprotein E | -249.88 | 0.017 |
| 1451335_at | placenta-specific 8 | -7.9 | 0.021 |
| 1451343_at | RIKEN cDNA 2210415M20 gene | -110.7 | 0.039 |
| 1451399_at | brain protein 17 | -26.12 | 0.046 |
| 1451820_at | DIRAS family, GTP-binding RAS-like 1 | -4.18 | 0.042 |
| 1451988_s_at | chromatin modifying protein 4B | -133.6 | 0.036 |
| 1452016_at | arachidonate 5-lipoxygenase activating protein | -4.76 | 0.018 |
| 1452680_at | small nuclear ribonucleoprotein D2 | -12.22 | 0.013 |
| 1452758_s_at | eukaryotic translation initiation factor 4, gamma 2 | -151.95 | 0.006 |
| 1452790_x_at | NADH dehydrogenase (ubiquinone) 1 alpha subcomplex, 3 | -12.64 | 0.03 |
| 1452927_x_at | triosephosphate isomerase 1 | -66.96 | 0.033 |
| 1453096_x_at | ribosomal protein L27 | -45.33 | 0.041 |
| 1453848_s_at | zinc finger, BED domain containing 3 | -45.47 | 0.047 |
| 1454615_x_at | signal recognition particle 14 | -76.35 | 0.033 |
| 1454627_a_at | ribosomal protein L29 | -20.45 | 0.024 |
| 1454713_s_at | histidine decarboxylase | -50.93 | 0.048 |
| 1454979_at | diaphanous homolog 1 (Drosophila) | -35.19 | 0.042 |
| 1455039_a_at | transcriptional regulator, SIN3B (yeast) | -8.42 | 0.021 |
| 1455138_x_at | cofilin 1, non-muscle | -4.81 | 0.037 |
| 1455269_a_at | coronin, actin binding protein 1A | -163.05 | 0.038 |
| 1455348_x_at | ribosomal protein L29 | -39.74 | 0.038 |
| 1455662_x_at | ribosomal protein S17 | -64.69 | 0.013 |
| 1455806_x_at | NADH dehydrogenase (ubiquinone) 1 alpha subcomplex, 12 | -58.31 | 0.04 |
| 1455815_a_at | tryptophan 5-monooxygenase activation protein, beta polypeptide | -204.31 | 0.02 |
| 1455821_x_at | complement component 1, q subcomponent binding protein | -134.71 | 0.022 |
| 1455897_x_at | high mobility group nucleosomal binding domain 1 | -386.76 | 0.035 |
| 1455899_x_at | suppressor of cytokine signaling 3 | -163.99 | 0.035 |
| 1455900_x_at | transglutaminase 2, C polypeptide | -7.2 | 0.036 |
| 1455929_x_at | protein phosphatase 2 (formerly 2A), regulatory subunit A (PR 65), alpha isoform | -28.65 | 0.041 |
| 1455939_x_at | signal recognition particle 14 | -97.39 | 0.003 |
| 1455981_at | similar to 40S ribosomal protein S6 | -64.35 | 0.001 |
| 1455997_a_at | ubiquinol-cytochrome c reductase binding protein | -20.92 | 0.044 |
| 1456012_x_at | ribonuclease T2 | -193.45 | 0.041 |
| 1456015_x_at | NADH dehydrogenase (ubiquinone) flavoprotein 1 | -62.68 | 0.04 |
| 1456037_x_at | prolactin regulatory element binding | -113.33 | 0.026 |
| 1456174_x_at | N-myc downstream regulated gene 1 | -4.99 | 0.005 |
| 1456194_a_at | Parkinson disease (autosomal recessive, early onset) 7 | -18.28 | 0.007 |
| 1456205_x_at | tubulin cofactor a | -99.38 | 0.024 |
| 1456213_x_at | glutaminyl-tRNA synthetase | -133.68 | 0.032 |
| 1456227_x_at | retinoblastoma binding protein 7 | -78.43 | 0.024 |
| 1456245_x_at | vesicle-associated membrane protein 3 | -10.73 | 0.007 |
| 1456247_x_at | proteolipid protein 2 | -344.61 | 0.022 |
| 1456251_x_at | benzodiazepine receptor, peripheral | -220.66 | 0.04 |
| 1456310_a_at | RIKEN cDNA 2610002J02 gene | -55.6 | 0.016 |
| 1456312_x_at | gelsolin | -62.33 | 0.034 |
| 1456313_x_at | mitochondrial ribosomal protein L28 | -16.21 | 0.033 |
| 1456373_x_at | ribosomal protein S20 | -15.7 | 0.046 |
| 1456377_x_at | RIKEN cDNA 0610025L06 gene | -90.29 | 0.045 |
| 1456381_x_at | myeloid cell leukemia sequence 1 | -10.95 | 0.048 |
| 1456439_x_at | microtubule associated monoxygenase, calponin and LIM domain containing 1 | -192.69 | 0.014 |
| 1456726_x_at | glutaminyl-tRNA synthetase | -372.58 | 0.041 |
| 1456737_x_at | acetyl-Coenzyme A acyltransferase 1 | -195.47 | 0.029 |
| 1459986_a_at | ribosomal protein S17 | -12.09 | 0.001 |
| 1460008_x_at | ribosomal protein L31 | -130.56 | 0.037 |
| 1460198_a_at | proteasome (prosome, macropain) subunit, beta type 3 | -7.61 | 0.027 |
| 1460357_at | YTH domain family 2 | -134.84 | 0.025 |
| 1460362_at | RIKEN cDNA 2410001C21 gene | -5.02 | 0.009 |
| 1460424_at | RIKEN cDNA 1810008O21 gene | -8.72 | 0.049 |
| 1460561_x_at | selenoprotein W, muscle 1 | -7.8 | 0.048 |
| 1460680_a_at | ribosomal protein L23 | -38.26 | 0.041 |
| 1460701_a_at | mitochondrial ribosomal protein L52 | -323.24 | 0.025 |
| 1460707_at | protein tyrosine phosphatase 4a2 | -32.77 | 0.014 |
| 1420144_x_at | membrane associated DNA binding protein | -10.32 | 0.012 |
| 1420266_at | Mm.200210 | -20.39 | 0.031 |
| 1428152_a_at | Ribosomal protein L18A | -45.31 | 0.034 |
| 1430518_at | RIKEN cDNA 5430402E10 gene | -17.13 | 0.011 |
| 1430612_at | RIKEN cDNA 1810033B17 gene | -6.31 | 0.025 |
| 1431015_at | Mm.173165 | -5.46 | 0.015 |
| 1431151_at | similar to BC055815 protein | -4.65 | 0.021 |
| 1432023_a_at | RIKEN cDNA 1700019H03 gene | -12.32 | 0.017 |
| 1432412_at | RIKEN cDNA 9530004P13 gene | -3.19 | 0.03 |
| 1432731_at | RIKEN cDNA 5830437K03 gene | -5.31 | 0.018 |
| 1432770_at | RIKEN cDNA 0610040A22 gene | -3.68 | 0.046 |
| 1432945_at | RIKEN cDNA 5230400M06 gene | -13.33 | 0.035 |
| 1434917_at | cordon-bleu | -177.86 | 0.017 |
| 1435334_at | tetratricopeptide repeat domain 7 | -11.72 | 0.008 |
| 1435436_at | Transcribed locus | -184.3 | 0.032 |
| 1435925_at | G protein-coupled receptor kinase-interactor 2 | -4.94 | 0.05 |
| 1436151_x_at | cDNA sequence BC031781 | -15.81 | 0.036 |
| 1436514_at | glypican 4 | -5.98 | 0.015 |
| 1436530_at | CDNA clone MGC:107680 IMAGE:6766535, complete cds | -780.08 | 0.025 |
| 1437019_at | RIKEN cDNA 2200001I15 gene | -13.06 | 0.03 |
| 1437247_at | fos-like antigen 2 | -59.35 | 0.03 |
| 1437828_s_at | WD repeat domain 46 | -8.88 | 0.025 |
| 1437875_at | Mm.28879 | -25.89 | 0.017 |
| 1439292_at | Transcribed locus | -5.27 | 0.049 |
| 1439560_x_at | hypothetical gene supported by BC047216 | -163.6 | 0.044 |
| 1440375_at | Transcribed locus, moderately similar to XP_576460.1 | -8.27 | 0.039 |
| 1440924_at | M-phase phosphoprotein 1 | -3.4 | 0.031 |
| 1442880_at | 13 days embryo male testis cDNA, RIKEN full-length enriched library | -5.53 | 0.047 |
| 1443557_at | Mm.17602 | -14.95 | 0.001 |
| 1445175_at | Adult male aorta and vein cDNA, RIKEN full-length enriched library | -4.86 | 0.014 |
| 1445245_at | RIKEN cDNA B130065D12 gene | -6.37 | 0.041 |
| 1445269_at | Mitochondrial ribosomal protein S18C | -3.42 | 0.036 |
| 1445532_at | RIKEN cDNA C230040D10 gene | -6.2 | 0.028 |
| 1445558_at | Mm.211355 | -4.9 | 0.036 |
| 1446251_at | hypothetical protein 4930565A17 | -5.44 | 0.037 |
| 1446896_at | expressed sequence AU022186 | -3.47 | 0.028 |
| 1447124_at | Transcribed locus | -2.59 | 0.049 |
| 1447291_at | nephrosis 1 homolog, nephrin antisense | -5.76 | 0.031 |
| 1447320_x_at | RNA polymerase 1-3 | -103.04 | 0.014 |
| 1447612_x_at | Hypothetical LOC74273 | -34.65 | 0.01 |
| 1447715_x_at | peroxiredoxin 6, related sequence 1 | -5.48 | 0.026 |
| 1447734_x_at | aldolase 1, A isoform | -25.82 | 0.027 |
| 1447750_x_at | RIKEN cDNA 1110061L23 gene | -10.6 | 0.038 |
| 1447783_x_at | DNA segment, Chr 11, ERATO Doi 333, expressed | -11.38 | 0.034 |
| 1447882_x_at | DEAD (Asp-Glu-Ala-Asp) box polypeptide 54 | -21.88 | 0.018 |
| 1453201_at | RIKEN cDNA 4632411J06 gene | -4.9 | 0.03 |
| 1453478_at | POU domain, class 3, transcription factor 2 | -9.47 | 0.017 |
| 1454248_at | RIKEN cDNA 1700041E20 gene | -5.12 | 0.047 |
| 1454342_at | RIKEN cDNA C030007D22 gene | -4.89 | 0.017 |
| 1455345_at | PHD finger protein 15 | -21.62 | 0.049 |
| 1455660_at | Colony stimulating factor 2 receptor, beta 1 | -230.06 | 0.005 |
| 1455852_at | RIKEN cDNA 4833432M17 gene | -6.42 | 0.017 |
| 1456308_x_at | tripartite motif protein 28 | -12.17 | 0.04 |
| 1456531_x_at | PRP19/PSO4 homolog (S. cerevisiae) | -209.26 | 0.025 |
| 1456949_at | RIKEN cDNA 6430502G17 gene | -9.18 | 0.03 |
| 1457643_x_at | RIKEN cDNA 2610316D01 gene | -20.82 | 0.005 |
| 1457693_a_at | RIKEN cDNA 6430537H07 gene | -6.28 | 0.025 |
| 1457824_at | Transcribed locus | -21.22 | 0.014 |
| 1458197_x_at | a disintegrin and metalloproteinase domain 1a | -3.49 | 0.002 |
