# Supplementary material for: Systemic Disease-Induced Salivary Biomarker Profiles in Mouse Models of Melanoma and Non-Small Cell Lung Cancer
Source: PLoS One. 2009 Jun 11;4(6):e5875. doi: 10.1371/journal.pone.0005875 (PMC2691577; doi:10.1371/journal.pone.0005875)
Supplement: Table S5 — (0.38 MB DOC) [file pone.0005875.s005.doc]

**Supplementary Table 5**. The list of 290 up-regulated salivary transcripts in lung cancer mouse model.

| Probe set | Gene | Fold change | P value |
| --- | --- | --- | --- |
| 1416400_at | pyrroline-5-carboxylate reductase-like | 3.22 | 0.012 |
| 1416434_at | Bcl2-like 10 | 9.3 | 0.02 |
| 1416600_a_at | Down syndrome critical region homolog 1 (human) | 10.85 | 0.002 |
| 1416616_s_at | caseinolytic protease, ATP-dependent, proteolytic subunit homolog | 2.45 | 0.025 |
| 1416755_at | DnaJ (Hsp40) homolog, subfamily B, member 1 | 3.89 | 0.041 |
| 1416821_at | expressed sequence 2 embryonic lethal | 2.4 | 0.033 |
| 1417092_at | parathyroid hormone receptor 1 | 6.47 | 0.019 |
| 1417116_at | solute carrier family 6, member 8 | 3.95 | 0.006 |
| 1417120_at | DNA segment, Chr 4, Wayne State University 114, expressed | 6.38 | 0.032 |
| 1417152_at | BTB (POZ) domain containing 14A | 2.28 | 0.033 |
| 1417286_at | NADH dehydrogenase (ubiquinone) 1 alpha subcomplex, 5 | 2.11 | 0.05 |
| 1418080_at | UDP-Gal:betaGlcNAc beta 1,4- galactosyltransferase, polypeptide 2 | 2.67 | 0.007 |
| 1418411_at | F-box and leucine-rich repeat protein 8 | 9.34 | 0.036 |
| 1418476_at | cytokine receptor-like factor 1 | 2.93 | 0.021 |
| 1418702_a_at | RIKEN cDNA 2810428I15 gene | 3.7 | 0.004 |
| 1418951_at | RIKEN cDNA 2310001N14 gene | 7.32 | 0.02 |
| 1419127_at | neuropeptide Y | 2.11 | 0.031 |
| 1419150_at | myogenic factor 6 | 3.43 | 0.019 |
| 1419283_s_at | tensin 1 | 3.06 | 0.024 |
| 1419489_at | expressed sequence AW049604 | 6.44 | 0.024 |
| 1419746_at | Rho GTPase activating protein 23 | 22.82 | 0.006 |
| 1419970_at | solute carrier family 35, member A5 | 3.74 | 0.024 |
| 1420085_at | fibroblast growth factor 4 | 4.81 | 0.005 |
| 1420086_x_at | fibroblast growth factor 4 | 4.6 | 0.037 |
| 1420336_at | thrombospondin, type I domain containing 6 | 2.68 | 0.047 |
| 1420352_at | protease, serine, 22 | 8.23 | 0.018 |
| 1420466_at | salivary protein 2 | 3.47 | 0.037 |
| 1420488_at | mitochondrial ribosomal protein S14 | 3.94 | 0.024 |
| 1420694_a_at | dachshund 1 (Drosophila) | 2.63 | 0.049 |
| 1420812_at | histone deacetylase 7A | 3.53 | 0.011 |
| 1421279_at | laminin, gamma 2 | 2.58 | 0.043 |
| 1421829_at | adenylate kinase 3 alpha-like 1 | 2.01 | 0.031 |
| 1421842_a_at | secretory carrier membrane protein 4 | 3.85 | 0.026 |
| 1422071_at | lectin, galactose binding, soluble 6 | 3.42 | 0.023 |
| 1422157_a_at | integrin beta 1 binding protein 1 | 4.68 | 0.032 |
| 1422314_at | chloride channel 6 | 2.28 | 0.049 |
| 1422395_at | vomeronasal 1 receptor, C22 | 2.59 | 0.023 |
| 1422402_a_at | ankyrin repeat, SAM and basic leucine zipper domain containing 1 | 3.8 | 0.045 |
| 1422971_at | GCN5 general control of amino acid synthesis-like 2 (yeast) | 4.51 | 0.024 |
| 1423175_s_at | par-6 (partitioning defective 6) homolog beta (C. elegans) | 4.83 | 0.023 |
| 1423326_at | ectonucleoside triphosphate diphosphohydrolase 1 | 2.02 | 0.038 |
| 1423348_at | frizzled homolog 8 (Drosophila) | 4.66 | 0.035 |
| 1423358_at | RIKEN cDNA 1810009K13 gene | 2.35 | 0.027 |
| 1424075_at | RIKEN cDNA 9430016H08 gene | 9.49 | 0.034 |
| 1424102_at | autophagy-related 4B-like (yeast) | 4.99 | 0.032 |
| 1424225_at | ankyrin repeat and SOCS box-containing protein 8 | 3.58 | 0.02 |
| 1424445_at | transmembrane 4 superfamily member 5 | 8.88 | 0.014 |
| 1424751_at | activator of basal transcription | 2.87 | 0.036 |
| 1424878_at | leucine-rich repeats and calponin homology (CH) domain containing 4 | 3.46 | 0.026 |
| 1425118_at | spire homolog 2 (Drosophila) | 5.46 | 0.041 |
| 1425456_a_at | mitogen activated protein kinase kinase 3 | 2.4 | 0.005 |
| 1425604_at | v-crk sarcoma virus CT10 oncogene homolog (avian)-like | 2.41 | 0.007 |
| 1425648_at | tripartite motif-containing 60 | 3.88 | 0.02 |
| 1425866_a_at | pleckstrin homology domain containing, family A | 6.82 | 0.013 |
| 1426177_a_at | NK1 transcription factor related, locus 2 (Drosophila) | 2.2 | 0.026 |
| 1426270_at | SMC5 structural maintenance of chromosomes 5-like 1 (yeast) | 2.94 | 0.047 |
| 1426352_s_at | Tial1 cytotoxic granule-associated RNA binding protein-like 1 | 2.11 | 0.037 |
| 1426440_at | dehydrogenase/reductase (SDR family) member 7 | 4.79 | 0.015 |
| 1426561_a_at | nephronectin | 3.3 | 0.046 |
| 1426567_a_at | PQ loop repeat containing 1 | 4.02 | 0.026 |
| 1426665_at | katanin p80 (WD40-containing) subunit B 1 | 6.31 | 0.005 |
| 1426725_s_at | E26 avian leukemia oncogene 1, 5' domain | 5.81 | 0.004 |
| 1426753_at | PHD finger protein 17 | 4.99 | 0.011 |
| 1426975_at | RIKEN cDNA 4632413K17 gene | 90.43 | 0.002 |
| 1427285_s_at | metastasis associated lung adenocarcinoma transcript 1 | 3.67 | 0.028 |
| 1427454_at | homeo box C6 | 6.17 | 0.04 |
| 1427570_at | signal recognition particle receptor ('docking protein') | 2.72 | 0.021 |
| 1428047_s_at | zinc finger protein, autosomal | 2.65 | 0.041 |
| 1428277_at | RIKEN cDNA 2600013N14 gene | 3.46 | 0.042 |
| 1428328_at | nucleoporin 50 | 2.84 | 0.019 |
| 1428353_at | forkhead box K2 | 4.64 | 0.042 |
| 1428966_at | RIKEN cDNA 2610204K14 gene | 13.6 | 0.021 |
| 1429128_x_at | nuclear factor of kappa light polypeptide gene enhancer in B-cells 2 | 2.56 | 0.01 |
| 1429568_x_at | RIKEN cDNA 2510010F15 gene | 2.84 | 0.012 |
| 1429715_at | protein phosphatase 2 (formerly 2A), regulatory subunit B (PR 52) | 13.54 | 0.03 |
| 1431753_x_at | RIKEN cDNA 2900073H19 gene | 7.12 | 0.011 |
| 1431791_a_at | protein tyrosine phosphatase, non-receptor type 13 | 2.68 | 0.048 |
| 1434003_a_at | deoxyhypusine synthase | 3.56 | 0.019 |
| 1434319_at | malate dehydrogenase 1, NAD (soluble) | 4.29 | 0.024 |
| 1434484_at | RIKEN cDNA 1100001G20 gene | 6.68 | 0.006 |
| 1434628_a_at | rhophilin, Rho GTPase binding protein 2 | 3.21 | 0.025 |
| 1434844_at | similar to hypothetical protein FLJ23825 | 2.93 | 0.023 |
| 1434964_at | expressed sequence AA408420 | 5.04 | 0.021 |
| 1435151_a_at | ribosomal protein S3 | 2.23 | 0.036 |
| 1435651_a_at | matrix-associated actin-dependent regulator of chromatin, subfamily a | 6.61 | 0.005 |
| 1437279_x_at | syndecan 1 | 3.35 | 0.017 |
| 1437618_x_at | G protein-coupled receptor 85 | 4.01 | 0.012 |
| 1438602_s_at | mannan-binding lectin serine protease 1 | 2 | 0.047 |
| 1438950_x_at | expressed sequence AI114950 | 3.31 | 0.05 |
| 1439350_s_at | CDC91 cell division cycle 91-like 1 (S. cerevisiae) | 7.34 | 0.014 |
| 1439415_x_at | ribosomal protein S21 | 2.81 | 0.039 |
| 1443416_at | expressed sequence C79741 | 5.46 | 0.034 |
| 1448308_at | adaptor-related protein complex 3, mu 1 subunit | 2.77 | 0.047 |
| 1448545_at | syndecan 2 | 3.18 | 0.01 |
| 1448562_at | uridine phosphorylase 1 | 7.43 | 0.047 |
| 1448600_s_at | vav 3 oncogene | 2.55 | 0.021 |
| 1448616_at | dishevelled 2, dsh homolog (Drosophila) | 3.01 | 0.005 |
| 1448675_at | DNA segment, Chr 1, ERATO Doi 161, expressed | 2.33 | 0.047 |
| 1448695_at | protein kinase C, iota | 8.18 | 0.015 |
| 1448930_at | RIKEN cDNA 3010026O09 gene | 6.05 | 0.007 |
| 1449028_at | ras homolog gene family, member U | 5.54 | 0.02 |
| 1449311_at | BTB and CNC homology 1 | 4.61 | 0.021 |
| 1449359_at | paired box gene 1 | 2.38 | 0.047 |
| 1449444_a_at | microfibrillar-associated protein 1 | 3.5 | 0.024 |
| 1449714_at | RIKEN cDNA 5730472N09 gene | 7.26 | 0.013 |
| 1449831_at | RIKEN cDNA 1700055O19 gene | 3.62 | 0.028 |
| 1449854_at | nuclear receptor subfamily 0, group B, member 2 | 2.85 | 0.025 |
| 1450000_at | VNACHT, leucine rich repeat and PYD containing 4C | 4.69 | 0.037 |
| 1450103_a_at | pleckstrin homology, Sec7 and coiled-coil domains 2 | 4.59 | 0.025 |
| 1450154_at | folate hydrolase | 2.58 | 0.044 |
| 1450281_a_at | RIKEN cDNA 1700021K02 gene | 2.63 | 0.007 |
| 1450331_s_at | vomeronasal 2, receptor, 4 /// vomeronasal 2, receptor, 5 | 2.18 | 0.012 |
| 1450335_at | Mus musculus pore forming protein (Pfp) | 3.84 | 0.035 |
| 1450403_at | signal transducer and activator of transcription 2 | 3.14 | 0.038 |
| 1450425_a_at | RIKEN cDNA 2700062C07 gene | 4.35 | 0.013 |
| 1450541_at | plasmacytoma variant translocation 1 | 2.16 | 0.048 |
| 1450546_at | Alport syndrome, mental retardation | 3.11 | 0.039 |
| 1450602_at | vomeronasal 1 receptor, C7 | 4.51 | 0.024 |
| 1450748_at | sphingomyelin phosphodiesterase 3, neutral | 11.18 | 0.005 |
| 1451051_a_at | SCY1-like 1 (S. cerevisiae) | 4.37 | 0.027 |
| 1451307_at | mitochondrial ribosomal protein L14 | 4.16 | 0.033 |
| 1451423_at | RIKEN cDNA 2810055C19 gene | 3.36 | 0.004 |
| 1451513_x_at | serine (or cysteine) proteinase inhibitor, clade A, member 1b | 2.53 | 0.022 |
| 1451668_at | RIKEN cDNA C530043G21 gene | 5.12 | 0.042 |
| 1451687_a_at | transcription factor 2 | 4.42 | 0.046 |
| 1451772_at | two pore channel 1 | 2.58 | 0.05 |
| 1451801_at | triadin | 4.38 | 0.042 |
| 1451957_at | interleukin 1 family, member 7 | 2 | 0.028 |
| 1452071_at | Solute carrier family 4 (anion exchanger), member 4 | 2.11 | 0.041 |
| 1452324_at | plasmacytoma variant translocation 1 | 5.34 | 0.027 |
| 1452344_at | synaptojanin 2 | 2.44 | 0.046 |
| 1453278_a_at | restin-like 2 | 4.21 | 0.026 |
| 1453920_a_at | motile sperm domain containing 2 | 2 | 0.044 |
| 1454021_a_at | exosome component 10 | 2.94 | 0.04 |
| 1454887_at | p21 (CDKN1A)-activated kinase 2 | 4 | 0.008 |
| 1456193_x_at | glutathione peroxidase 4 | 2.06 | 0.045 |
| 1456237_x_at | Similar to hypothetical protein FLJ20397 | 2.8 | 0.031 |
| 1456456_x_at | Melanoma antigen | 13.41 | 0.046 |
| 1456699_s_at | RIKEN cDNA A730098D12 gene | 2.61 | 0.038 |
| 1460349_at | cDNA sequence BC006909 | 3.31 | 0.029 |
| 1460375_at | RIKEN cDNA 0610038D11 gene | 2.6 | 0.03 |
| 1460519_a_at | methyltransferase like 5 | 2.43 | 0.04 |
| 1460670_at | RIO kinase 3 (yeast) | 4.2 | 0.021 |
| 1420006_at | Bone morphogenetic protein 15 | 4.45 | 0.028 |
| 1420208_at | DB_XREF=AV075907 | 3.5 | 0.029 |
| 1428284_at | RIKEN cDNA 8430427H17 gene | 3.77 | 0.016 |
| 1428581_at | RIKEN cDNA 1700024G10 gene | 2.39 | 0.029 |
| 1428901_at | DTW domain containing 2 | 3.18 | 0.033 |
| 1429204_at | RIKEN cDNA 2900075A18 gene | 2.04 | 0.05 |
| 1429320_at | RIKEN cDNA 4921511I16 gene | 4.03 | 0.023 |
| 1429377_at | RIKEN cDNA 2410004A20 gene | 2.61 | 0.031 |
| 1429509_at | RIKEN cDNA 1110032E16 gene | 2.26 | 0.05 |
| 1429772_at | plexin A2 | 2.77 | 0.031 |
| 1429971_at | thioredoxin reductase 2 | 6.17 | 0.006 |
| 1430224_at | WAP four-disulfide core domain 3 | 3.87 | 0.009 |
| 1430228_at | denticleless homolog (Drosophila) | 3.88 | 0.027 |
| 1430411_at | RIKEN cDNA 6330525I24 gene | 5.74 | 0.009 |
| 1430964_at | RIKEN cDNA 2310034O05 gene | 3.15 | 0.025 |
| 1431101_a_at | steroid 5 alpha-reductase 1 | 5.14 | 0.03 |
| 1431305_at | RIKEN cDNA 2210015I05 gene | 3.04 | 0.01 |
| 1431499_at | RIKEN cDNA 4933436F18 gene | 2.21 | 0.049 |
| 1431546_at | RIKEN cDNA 4930509K18 gene | 2.84 | 0.044 |
| 1431622_at | RIKEN cDNA 4933406K04 gene | 11.98 | 0.029 |
| 1431717_at | RIKEN cDNA 3526401B18 gene | 4.75 | 0.047 |
| 1431902_at | RIKEN cDNA 4930401A09 gene | 2.5 | 0.047 |
| 1432070_at | RIKEN cDNA 4921511C10 gene | 3.29 | 0.025 |
| 1432076_at | RIKEN cDNA 4933430H16 gene | 12.69 | 0.007 |
| 1432098_a_at | olfactory receptor 701 | 4.09 | 0.023 |
| 1432247_at | RIKEN cDNA 4633402D09 gene | 3.44 | 0.03 |
| 1432314_at | RIKEN cDNA 4930554C24 gene | 7.35 | 0.035 |
| 1432484_at | RIKEN cDNA 2310061C15 gene | 5.81 | 0.014 |
| 1432510_at | RIKEN cDNA 4930512M02 gene | 3.19 | 0.046 |
| 1432698_at | RIKEN cDNA 2900024I21 gene | 4.04 | 0.043 |
| 1432795_at | RIKEN cDNA 2310058F05 gene | 2.2 | 0.022 |
| 1432935_at | RIKEN cDNA 5330433J24 gene | 4.74 | 0.029 |
| 1433089_at | RIKEN cDNA 4930404F17 gene | 2.59 | 0.033 |
| 1433297_at | RIKEN cDNA 1700039M15 gene | 5.87 | 0.007 |
| 1433426_at | RIKEN cDNA 1700111A04 gene | 3.23 | 0.033 |
| 1433544_at | amyotrophic lateral sclerosis 2 chromosome region | 3.15 | 0.035 |
| 1433673_at | similar to limkain beta 2 | 2.06 | 0.041 |
| 1433818_at | 1-acylglycerol-3-phosphate O-acyltransferase 3 | 2.17 | 0.048 |
| 1434771_at | RIKEN cDNA 0610011F06 gene | 15.47 | 0.029 |
| 1435060_at | tropomodulin 2 | 3.69 | 0.029 |
| 1435334_at | tetratricopeptide repeat domain 7 | 12.65 | 0.013 |
| 1435392_at | WD repeat domain 17 | 2.79 | 0.028 |
| 1435482_at | fibrinogen C domain containing 1 | 2.24 | 0.044 |
| 1435805_at | lin 7 homolog a (C. elegans) | 5.22 | 0.027 |
| 1435902_at | nudix (nucleoside diphosphate linked moiety X)-type motif 18 | 8.19 | 0.037 |
| 1435987_x_at | RIKEN cDNA 1110059G02 gene | 4.44 | 0.047 |
| 1436069_at | UG_TITLE=expressed sequence AI225768 | 2.89 | 0.021 |
| 1436397_at | cDNA sequence BC027057 | 17.44 | 9E-04 |
| 1436897_at | malignant fibrous histiocytoma amplified sequence 1 | 2.84 | 0.025 |
| 1437073_x_at | expressed sequence AV025504 | 3.98 | 0.043 |
| 1437283_at | transportin 2 (importin 3, karyopherin beta 2b) | 3.7 | 0.019 |
| 1437972_s_at | splicing factor 3b, subunit 5 | 2.36 | 0.039 |
| 1438112_at | RIKEN cDNA 9430021M05 gene | 5.27 | 0.008 |
| 1438519_at | RIKEN cDNA 4930429H24 gene | 2.96 | 0.025 |
| 1439117_at | calmin | 2.34 | 0.042 |
| 1439127_at | expressed sequence AI314180 | 3.05 | 0.042 |
| 1439632_at | Guanine nucleotide binding protein, beta 4 | 2.61 | 0.035 |
| 1439745_at | calcium channel, voltage-dependent, gamma subunit 7 | 2.59 | 0.033 |
| 1440105_at | hypothetical protein 4932416A15 | 5.72 | 0.048 |
| 1440186_s_at | Transcribed locus | 2.91 | 0.025 |
| 1440393_at | Odd Oz/ten-m homolog 2 (Drosophila) | 15.2 | 0.002 |
| 1440407_at | Mm.139841,UG_TITLE | 2.39 | 0.048 |
| 1440476_at | DNA segment, Chr 6, ERATO Doi 474, expressed | 3.29 | 0.048 |
| 1440663_at | Exportin 4 | 3.33 | 0.024 |
| 1440719_at | GLE1 RNA export mediator-like (yeast | 3.28 | 0.037 |
| 1440909_at | interferon inducible GTPase family member 5 | 8.58 | 0.048 |
| 1441095_at | RIKEN cDNA A430041B07 gene | 3.19 | 0.027 |
| 1441240_at | Replication protein A1 | 3.75 | 0.015 |
| 1441246_s_at | dihydropyrimidinase-like 5 | 2.12 | 0.043 |
| 1441274_at | RIKEN cDNA 4121402D02 gene | 2.37 | 0.017 |
| 1441740_at | Braf transforming gene | 5.79 | 0.017 |
| 1441792_at | RIKEN cDNA A630033E08 gene | 2.35 | 0.028 |
| 1441891_x_at | ELOVL family member 7, elongation of long chain fatty acids | 4.73 | 0.04 |
| 1442010_at | castor homolog 1, zinc finger (Drosophila) | 2.33 | 0.03 |
| 1442022_at | RIKEN cDNA E430004N04 gene | 5.66 | 0.017 |
| 1442128_at | Deleted in polyposis 1 | 5.22 | 0.011 |
| 1442286_at | RIKEN cDNA D330013L20 gene | 2.39 | 0.05 |
| 1442328_at | Transcribed locus | 5.13 | 0.033 |
| 1442455_at | expressed sequence C79845 | 3.89 | 0.016 |
| 1442859_at | Sprouty protein with EVH-1 domain 1, related sequence | 2.33 | 0.044 |
| 1442950_at | RIKEN cDNA A830055N07 gene | 2.36 | 0.022 |
| 1443120_at | PDZ domain containing 4 | 5.31 | 0.008 |
| 1443261_at | RIKEN cDNA E130307A14 gene | 2.09 | 0.045 |
| 1443307_at | Proteasome (prosome, macropain) 26S subunit, ATPase 2 | 2.22 | 0.046 |
| 1443395_at | DNA segment, Chr 6, ERATO Doi 490, expressed | 3.56 | 0.044 |
| 1443600_at | expressed sequence AA414992 | 2.46 | 0.026 |
| 1443765_at | RIKEN cDNA 1810058N05 gene | 3.03 | 0.048 |
| 1443808_at | DNA segment, Chr 2, Brigham & Women's Genetics 1423 expressed | 7.36 | 0.008 |
| 1444380_at | expressed sequence AI844869 | 3.07 | 0.045 |
| 1444431_at | RCSD domain containing 1 | 2.04 | 0.024 |
| 1444671_at | RIKEN cDNA 2810025M15 gene | 4.58 | 0.036 |
| 1444723_at | RIKEN cDNA 6530418L21 gene | 2.67 | 0.049 |
| 1444782_at | RIKEN cDNA 4930417H01 gene | 2.26 | 0.032 |
| 1444891_at | expressed sequence AU015621 | 2.64 | 0.045 |
| 1445281_a_at | RIKEN cDNA B230311B06 gene | 4.71 | 0.045 |
| 1445289_at | H3098C05-3 | 2.58 | 0.038 |
| 1445323_at | gene regulated by estrogen in breast cancer protein | 3.63 | 0.03 |
| 1445550_at | Transcribed locus | 2.44 | 0.02 |
| 1445871_at | expressed sequence AI315376 | 4.97 | 0.044 |
| 1446009_at | Mus musculus similar to Potassium voltage-gated channel subfamily G member 2 ( | 7.1 | 0.041 |
| 1446283_at | Dedicator of cytokinesis 4 | 2.49 | 0.011 |
| 1446787_at | COMM domain containing 1 | 2.68 | 0.031 |
| 1447535_at | DB_XREF=UI-M-BH2.3-anx-b-01-0-UI.s2 | 3.67 | 0.035 |
| 1447621_s_at | RIKEN cDNA 2610307O08 gene | 2.26 | 0.026 |
| 1447642_x_at | dystrophia myotonica-containing WD repeat motif | 2.41 | 0.035 |
| 1447662_x_at | DNA segment, Chr 18, ERATO Doi 653, expressed | 3.23 | 0.027 |
| 1447747_x_at | D330039O10 | 3.42 | 0.029 |
| 1447751_x_at | dihydrouridine synthase 2-like (SMM1, S. cerevisiae) | 8.97 | 0.016 |
| 1447873_x_at | BH3 interacting domain death agonist | 2.35 | 0.036 |
| 1447949_at | RIKEN cDNA 2210018M03 gene | 5.11 | 0.031 |
| 1447974_s_at | aquaporin 6 | 3.33 | 0.007 |
| 1452883_a_at | RIKEN cDNA 2310002J21 gene | 5.73 | 0.045 |
| 1452948_at | tumor necrosis factor, alpha-induced protein 8-like 2 | 5.16 | 0.016 |
| 1452988_at | AP2 associated kinase 1 | 10.82 | 0.026 |
| 1453245_at | RIKEN cDNA 9130024F11 gene | 2.93 | 0.043 |
| 1453390_at | RIKEN cDNA 4930428F12 gene | 3.72 | 0.005 |
| 1454216_at | RIKEN cDNA 4930408F14 gene | 4.03 | 0.023 |
| 1454242_at | RIKEN cDNA 2310079G19 gene | 4.53 | 0.04 |
| 1454376_at | RIKEN cDNA 4833410I11 gene | 3.04 | 0.03 |
| 1454394_at | RIKEN cDNA 4932432N04 gene | 3.37 | 0.021 |
| 1455952_at | ADP-ribosylhydrolase like 1 | 2.04 | 0.033 |
| 1456230_at | similar to Tripartite motif protein 47 | 3.53 | 0.039 |
| 1456318_at | C-type lectin domain family 1, member a | 3.13 | 0.034 |
| 1456363_at | Suppression of tumorigenicity 7-like | 14.59 | 0.031 |
| 1456631_at | AV272901 | 2.5 | 0.033 |
| 1456895_at | CD209b antigen | 4.4 | 0.011 |
| 1456953_at | 12 days embryo spinal ganglion cDNA, RIKEN full-length enriched library | 2.5 | 0.023 |
| 1457028_at | hypothetical protein A430033K04 | 8.11 | 0.03 |
| 1457413_at | Receptor tyrosine kinase-like orphan receptor 1 | 7.14 | 0.009 |
| 1457474_at | Transcribed locus | 7.25 | 0.026 |
| 1457643_x_at | RIKEN cDNA 2610316D01 gene | 2.28 | 0.035 |
| 1457818_at | Mm.152086.1 | 2.44 | 0.049 |
| 1458071_at | Mm.185319 | 5.05 | 0.029 |
| 1458087_at | hypothetical protein 9830125E18 | 6.89 | 0.048 |
| 1458102_at | Plexin A2 | 3.37 | 0.032 |
| 1458384_at | RIKEN cDNA 1810056O20 gene | 2.31 | 0.049 |
| 1458401_at | RIKEN cDNA 4932438A13 gene | 2.94 | 0.017 |
| 1458764_at | Mm.218055 | 6.65 | 0.036 |
| 1458770_at | DNA segment, Chr 11, ERATO Doi 4, expressed | 5.83 | 0.006 |
| 1458962_at | transmembrane emp24 protein transport domain containing 5 | 4.65 | 0.021 |
| 1459102_at | Myosin ID | 3.3 | 0.023 |
| 1459457_at | calcium/calmodulin-dependent protein kinase II, delta | 3.87 | 0.015 |
| 1459653_at | PREDICTED: Mus musculus LOC434194 (LOC434194), mRNA | 4.46 | 0.023 |
| 1459679_s_at | myosin IB | 4.55 | 0.039 |
| 1459836_x_at | splicing factor 3b, subunit 2 | 2.5 | 0.008 |
| 1460119_at | BB245904 | 3.78 | 0.022 |
| 1460632_at | retinol dehydrogenase 10 (all-trans) | 21.41 | 0.02 |
|  |  |  |  |
