# Supplementary material for: Systemic Disease-Induced Salivary Biomarker Profiles in Mouse Models of Melanoma and Non-Small Cell Lung Cancer
Source: PLoS One. 2009 Jun 11;4(6):e5875. doi: 10.1371/journal.pone.0005875 (PMC2691577; doi:10.1371/journal.pone.0005875)
Supplement: Table S6 — (0.98 MB DOC) [file pone.0005875.s006.doc]

**Supplementary Table 6**. The list of 784 down-regulated salivary transcripts in lung cancer mouse model.

| Probe set | Gene | Fold change | P value |
| --- | --- | --- | --- |
| 1415691_at | discs, large homolog 1 (Drosophila) | -5.63 | 0.013 |
| 1415717_at | RIKEN cDNA 4931406I20 gene | -2.94 | 0.03 |
| 1415794_a_at | spindlin | -2.56 | 0.049 |
| 1415877_at | dihydropyrimidinase-like 3 | -3.25 | 0.012 |
| 1415914_at | heterogeneous nuclear ribonucleoprotein A/B | -4.37 | 0.016 |
| 1416021_a_at | fatty acid binding protein 5, epidermal member 4 | -4.24 | 0.014 |
| 1416022_at | fatty acid binding protein 5, epidermal,member 5 | -7.62 | 0.03 |
| 1416046_a_at | fucosidase, alpha-L- 2, plasma | -6.63 | 0.022 |
| 1416059_at | SEC23B (S. cerevisiae) | -6.14 | 0.038 |
| 1416087_at | adaptor protein complex AP-1, sigma 1 | -19.44 | 0.019 |
| 1416153_at | signal recognition particle 54 | -5.31 | 0.008 |
| 1416260_a_at | sorting nexin 1 | -24.3 | 0.021 |
| 1416544_at | enhancer of zeste homolog 2 (Drosophila) | -5.67 | 0.012 |
| 1416548_at | solute carrier family 35, member B4 | -3.85 | 0.032 |
| 1416555_at | etoposide induced 2.4 mRNA | -8.24 | 0.033 |
| 1416633_a_at | RIKEN cDNA 5730536A07 gene | -3.58 | 0.023 |
| 1416668_at | RIKEN cDNA 4921531G14 gene | -64.27 | 0.028 |
| 1416791_a_at | nuclear RNA export factor 1 homolog | -9.67 | 0.038 |
| 1416792_at | protein phosphatase 1G (formerly 2C), magnesium-dependent, gamma isoform | -17.72 | 0.012 |
| 1416796_at | non-catalytic region of tyrosine kinase adaptor protein 2 | -3.63 | 0.03 |
| 1416860_s_at | inhibitor of growth family, member 1 | -5.3 | 0.024 |
| 1416897_at | poly (ADP-ribose) polymerase family, member 9 | -2.84 | 0.048 |
| 1416909_at | RIKEN cDNA 1810008A14 gene | -4.63 | 0.037 |
| 1416922_a_at | BCL2/adenovirus E1B 19kDa-interacting protein 3-like | -4.37 | 0.015 |
| 1417050_at | C1q and tumor necrosis factor related protein 4 | -4.65 | 0.021 |
| 1417141_at | interferon gamma induced GTPase | -4.93 | 0.012 |
| 1417181_a_at | kinesin-associated protein 3 | -5.72 | 0.031 |
| 1417351_a_at | small nuclear ribonucleoprotein polypeptide A' | -29.15 | 0.02 |
| 1417353_x_at | small nuclear ribonucleoprotein polypeptide A',ab | -79.85 | 0.017 |
| 1417470_at | apolipoprotein B editing complex 3 | -10.26 | 0.012 |
| 1417472_at | myosin, heavy polypeptide 9, non-muscle | -2.29 | 0.021 |
| 1417524_at | cornichon homolog 2 (Drosophila) | -2.4 | 0.034 |
| 1417572_at | N-methylpurine-DNA glycosylase | -4.06 | 0.04 |
| 1417703_at | poliovirus receptor-related 2 | -4.55 | 0.046 |
| 1417733_at | ring finger protein 146 | -10.59 | 0.035 |
| 1417740_at | cell division cycle 37 homolog (S. cerevisiae)-like 1 | -14.03 | 0.01 |
| 1417770_s_at | Mus musculus RIKEN cDNA 2300001E01 gene | -35.22 | 0.028 |
| 1417799_at | ATPase, H+ transporting, V1 subunit G isoform 2 | -2.9 | 0.031 |
| 1417849_at | glucocorticoid induced gene 1 | -4.82 | 0.028 |
| 1417851_at | chemokine (C-X-C motif) ligand 13 | -22.22 | 0.026 |
| 1417953_at | DNA segment, Chr 6, Wayne State University 176, expressed | -30.44 | 0.047 |
| 1418007_at | RIKEN cDNA 1810007M14 gene | -23.48 | 0.049 |
| 1418011_a_at | SH3-domain GRB2-like B1 (endophilin) | -9.9 | 0.038 |
| 1418014_a_at | betaGlcNAc beta 1,4- galactosyltransferase, polypeptide 1 | -6.24 | 0.013 |
| 1418087_at | ubiquitin fusion degradation 1 like | -10.73 | 0.035 |
| 1418091_at | transcription factor CP2-like 1 | -2.44 | 0.049 |
| 1418131_at | SAM domain and HD domain, 1 | -4.17 | 0.009 |
| 1418156_at | potassium voltage-gated channel, Isk-related subfamily, gene 4 | -2.87 | 0.047 |
| 1418189_s_at | receptor (calcitonin) activity modifying protein 2 | -3.53 | 0.018 |
| 1418201_at | pleckstrin homology domain containing, family G member 2 | -2.36 | 0.044 |
| 1418229_s_at | histone cell cycle regulation defective interacting protein 5 | -11.33 | 0.013 |
| 1418242_at | Fas-associated factor 1 | -9.94 | 9E-04 |
| 1418249_at | calcitonin gene-related peptide-receptor component protein | -5.46 | 0.018 |
| 1418259_a_at | ectonucleoside triphosphate diphosphohydrolase 2 | -6.08 | 0.013 |
| 1418333_at | metal response element binding transcription factor 1 | -50.4 | 0.018 |
| 1418338_at | WD repeat domain 33 | -6.6 | 0.037 |
| 1418451_at | guanine nucleotide binding protein (G protein), gamma 2 subunit | -3.39 | 0.039 |
| 1418483_a_at | glycoprotein galactosyltransferase alpha 1, 3 | -3.33 | 0.046 |
| 1418540_a_at | protein tyrosine phosphatase, receptor type, E | -50.64 | 0.006 |
| 1418553_at | rho/rac guanine nucleotide exchange factor (GEF) 18 | -15.63 | 0.038 |
| 1418669_at | Mus musculus perlecan (heparan sulfate proteoglycan 2) (Hspg2) | -2.05 | 0.006 |
| 1418697_at | indolethylamine N-methyltransferase | -3.1 | 0.043 |
| 1418761_at | insulin-like growth factor 2, binding protein 1 | -6.26 | 0.031 |
| 1418790_at | zinc finger protein 312 | -9.44 | 0.033 |
| 1418806_at | colony stimulating factor 3 receptor (granulocyte) | -2.92 | 0.038 |
| 1418924_at | RIKEN cDNA 2400009B11 gene | -3.97 | 0.016 |
| 1418928_a_at | RIKEN cDNA 2310038H17 gene | -2.85 | 0.024 |
| 1419072_at | glutathione S-transferase, mu 7 | -2.26 | 0.05 |
| 1419079_at | sodium channel, nonvoltage-gated 1 gamma | -5.48 | 0.028 |
| 1419089_at | tissue inhibitor of metalloproteinase 3 | -10.02 | 0.029 |
| 1419124_at | RIKEN cDNA 2210010L05 gene | -8.26 | 0.013 |
| 1419256_at | spectrin beta 2 | -14.28 | 0.015 |
| 1419273_at | expressed sequence C80913 | -12.59 | 0.013 |
| 1419275_at | DAZ associated protein 1 | -5.13 | 0.01 |
| 1419363_a_at | mitochondrial ribosomal protein L35 | -12.59 | 0.027 |
| 1419367_at | 2,4-dienoyl CoA reductase 1, mitochondrial | -4.06 | 0.012 |
| 1419381_at | telomeric repeat binding factor 2, interacting protein | -3.64 | 0.036 |
| 1419439_at | serine/threonine kinase 22 substrate 1 | -6.34 | 0.035 |
| 1419452_at | ubiquitin carboxyl-terminal esterase L5 | -9.67 | 0.044 |
| 1419459_a_at | RIKEN cDNA 2610529C04 gene | -3.02 | 0.015 |
| 1419462_s_at | gene trap locus 3 | -6.77 | 0.021 |
| 1419516_at | DNA segment, human DXS9928E | -26.07 | 0.023 |
| 1419599_s_at | membrane-spanning 4-domains, subfamily A, member 11 | -5.4 | 0.022 |
| 1419634_a_at | growth hormone releasing hormone | -2.2 | 0.032 |
| 1419643_s_at | RIKEN cDNA 2310057J18 gene | -7.92 | 0.048 |
| 1419698_at | chemokine (C-X-C motif) ligand 11 | -4.99 | 0.047 |
| 1419736_a_at | eukaryotic translation initiation factor 1A, Y-linked | -7.69 | 0.007 |
| 1419835_s_at | plectin 1 | -7.94 | 0.038 |
| 1419869_s_at | high density lipoprotein (HDL) binding protein | -2.2 | 0.037 |
| 1419872_at | colony stimulating factor 1 receptor | -3.28 | 0.044 |
| 1420000_s_at | immunoglobulin (CD79A) binding protein 1 | -5.5 | 0.029 |
| 1420001_at | DNA segment, Chr 7, ERATO Doi 1, expressed | -7.48 | 0.021 |
| 1420057_at | Phosphatidylserine receptor | -2.92 | 0.021 |
| 1420123_at | T-cell leukemia translocation altered gene | -2.86 | 0.038 |
| 1420171_s_at | myosin, heavy polypeptide 9, non-muscle | -15.2 | 0.045 |
| 1420225_at | AV090440 | -3.36 | 0.043 |
| 1420569_at | chondroadherin | -3.87 | 0.043 |
| 1420621_a_at | amyloid beta (A4) precursor protein | -40.16 | 0.026 |
| 1420669_at | aryl hydrocarbon receptor nuclear translocator 2 | -3.72 | 0.036 |
| 1420741_x_at | RIKEN cDNA 2310069N01 gene | -2.74 | 0.028 |
| 1420780_at | achaete-scute complex homolog-like 3 (Drosophila) | -4.85 | 0.02 |
| 1420841_at | protein tyrosine phosphatase, receptor type, F | -3.41 | 0.034 |
| 1420882_a_at | adrenocortical dysplasia | -2.13 | 0.02 |
| 1420900_a_at | RAB18, member RAS oncogene family | -23.01 | 0.02 |
| 1420996_at | plexin A3 | -4.12 | 0.027 |
| 1421026_at | guanine nucleotide binding protein, alpha 12 | -2.48 | 0.047 |
| 1421049_at | RIKEN cDNA 0610009O20 gene | -3.86 | 0.015 |
| 1421077_at | SERTA domain containing 3 | -2.61 | 0.031 |
| 1421088_at | glypican 4 | -11.77 | 0.005 |
| 1421387_at | kringle containing transmembrane protein 1 | -2.54 | 0.046 |
| 1421410_a_at | proline-serine-threonine phosphatase-interacting protein 2 | -10.58 | 0.006 |
| 1421597_a_at | homeo box, msh-like 3 | -3.94 | 0.044 |
| 1421818_at | B-cell leukemia/lymphoma 6 | -14.98 | 0.032 |
| 1421904_at | nuclear receptor coactivator 6 interacting protein | -6.24 | 0.049 |
| 1421923_at | SH3-domain binding protein 5 (BTK-associated) | -5.74 | 0.009 |
| 1421944_a_at | asialoglycoprotein receptor 1 | -4.64 | 0.027 |
| 1422303_a_at | tumor necrosis factor receptor superfamily, member 18 | -85.51 | 0.044 |
| 1422461_at | ATPase family, AAA domain containing 3A | -5.5 | 0.034 |
| 1422478_a_at | acetyl-Coenzyme A synthetase 2 (ADP forming) | -35 | 8E-04 |
| 1422479_at | acetyl-Coenzyme A synthetase 2 (ADP forming) | -2.68 | 0.037 |
| 1422492_at | coproporphyrinogen oxidase | -42.76 | 0.045 |
| 1422518_at | calcium/calmodulin-dependent serine protein kinase (MAGUK family) | -24.57 | 0.022 |
| 1422533_at | cytochrome P450, family 51 | -2.54 | 0.031 |
| 1422742_at | human immunodeficiency virus type I enhancer binding protein 1 | -43.56 | 0.028 |
| 1422772_at | UDP-galactose:N-acetylgalactosamine-alpha-R beta 1,3-galactosyltransferase | -3.43 | 0.028 |
| 1422880_at | synaptophysin-like protein | -5.8 | 0.016 |
| 1422893_at | Scm-like with four mbt domains 1 | -2.6 | 0.035 |
| 1423108_at | solute carrier family 25 member 20 | -2.2 | 0.041 |
| 1423154_at | cDNA sequence BC005537 | -6.04 | 0.012 |
| 1423290_at | hypoxia up-regulated 1 | -3.54 | 0.033 |
| 1423332_at | syndecan binding protein | -2.69 | 0.047 |
| 1423349_at | suppressor of cytokine signaling 5 | -9.43 | 0.032 |
| 1423386_at | proteasome (prosome, macropain) 26S subunit, non-ATPase, 9 | -3.18 | 0.035 |
| 1423411_at | cDNA sequence BC013481 | -43.38 | 0.049 |
| 1423412_at | cDNA sequence BC013481 | -2.94 | 0.036 |
| 1423416_at | matrix associated, actin dependent regulator of chromatin, subfamily c, member 1 | -6.31 | 0.018 |
| 1423453_at | expressed sequence C78541 | -5.89 | 0.007 |
| 1423608_at | integral membrane protein 2A | -2.08 | 0.035 |
| 1423610_at | methionine aminopeptidase 2 | -9.38 | 0.021 |
| 1423632_at | G protein-coupled receptor 146 | -3.63 | 0.041 |
| 1423646_at | zinc finger, DHHC domain containing 3 | -3.75 | 0.014 |
| 1423703_at | peter pan homolog (Drosophila) | -32.52 | 0.034 |
| 1423785_at | EGL nine homolog 1 (C. elegans) | -9.54 | 0.008 |
| 1423795_at | splicing factor proline/glutamine rich | -19.58 | 0.033 |
| 1423862_at | pleckstrin homology domain containing, family F member 2 | -11.7 | 0.046 |
| 1423955_a_at | longevity assurance homolog 2 (S. cerevisiae) | -2.63 | 0.044 |
| 1424067_at | intercellular adhesion molecule | -3.84 | 0.038 |
| 1424068_at | T-cell leukemia translocation altered gene | -2.95 | 0.024 |
| 1424135_at | RIKEN cDNA 4930470D19 gene | -6.29 | 0.047 |
| 1424150_at | glycerophosphodiester phosphodiesterase domain containing 5 | -11.31 | 0.046 |
| 1424227_at | polymerase (RNA) III (DNA directed) polypeptide H | -2.65 | 0.043 |
| 1424276_at | sorting nexin 16 | -9.69 | 0.017 |
| 1424285_s_at | ADP-ribosylation factor-like 6 interacting protein 4 | -4.92 | 0.006 |
| 1424302_at | paired-Ig-like receptor B | -3.6 | 0.049 |
| 1424645_at | trinucleotide repeat containing 6C | -15.81 | 0.007 |
| 1424709_at | sterol-C5-desaturase (fungal ERG3, delta-5-desaturase) homolog | -4.05 | 0.012 |
| 1424712_at | AT hook containing transciption factor 1 | -9.11 | 0.04 |
| 1425004_s_at | molybdenum cofactor synthesis 1 | -3.08 | 0.045 |
| 1425011_x_at | syntaxin 18 | -21.36 | 0.011 |
| 1425088_at | sodium channel, nonvoltage-gated, type I, alpha | -3.95 | 0.047 |
| 1425187_at | Sel1 (suppressor of lin-12) 1 homolog (C. elegans) | -4.41 | 0.004 |
| 1425196_a_at | histidine triad nucleotide binding protein 2 | -12.98 | 0.031 |
| 1425314_at | monogenic, audiogenic seizure susceptibility 1 | -2.68 | 0.03 |
| 1425480_at | CCR4-NOT transcription complex, subunit 6-like | -42.52 | 0.005 |
| 1425567_a_at | annexin A5 | -3.66 | 0.028 |
| 1425786_a_at | heat shock transcription factor 4 | -2.18 | 0.046 |
| 1425926_a_at | orthodenticle homolog 2 (Drosophila) | -4.34 | 0.049 |
| 1426109_a_at | solute carrier family 14 (urea transporter), member 2 | -2.03 | 0.043 |
| 1426111_x_at | interferon regulatory factor 3 | -2.17 | 0.044 |
| 1426136_x_at | killer cell lectin-like receptor subfamily A, member 21 | -3.85 | 0.042 |
| 1426351_at | heat shock protein 1 (chaperonin) | -6.5 | 0.02 |
| 1426387_x_at | RIKEN cDNA 4933439C20 gene | -3.21 | 0.02 |
| 1426459_s_at | expressed sequence AW549877 | -2.15 | 0.013 |
| 1426525_at | AT rich interactive domain 2 (Arid-rfx like) | -7.74 | 0.021 |
| 1426557_at | mesoderm posterior 1 | -7.86 | 0.02 |
| 1426607_at | MRNA similar to hypothetical protein FLJ21657 | -3.28 | 0.021 |
| 1426633_s_at | potassium channel tetramerisation domain containing 14 | -28.33 | 0.049 |
| 1426722_at | solute carrier family 38, member 2 | -3.67 | 0.046 |
| 1426752_at | PHD finger protein 17 | -9.17 | 0.042 |
| 1426794_at | protein tyrosine phosphatase, receptor type, S | -5.13 | 0.047 |
| 1426797_at | RIKEN cDNA 2700094F01 gene | -3.3 | 0.041 |
| 1426815_s_at | expressed sequence AU024582 | -3.23 | 0.042 |
| 1426856_at | hydroxysteroid dehydrogenase like 2 | -3.21 | 0.006 |
| 1426886_at | ceroid-lipofuscinosis, neuronal 5 | -6.33 | 0.031 |
| 1426916_at | secernin 3 | -4.64 | 0.026 |
| 1427036_a_at | eukaryotic translation initiation factor 4, gamma 1 | -6.99 | 0.021 |
| 1427080_at | RIKEN cDNA 2610036D13 gene | -5.82 | 0.008 |
| 1427090_at | zinc finger, BED domain containing 4 | -2.53 | 0.038 |
| 1427096_s_at | signal sequence receptor, delta | -2.94 | 0.009 |
| 1427162_a_at | ELK4, member of ETS oncogene family | -9.28 | 0.024 |
| 1427177_at | FYVE and coiled-coil domain containing 1 | -40.93 | 0.037 |
| 1427347_s_at | RIKEN cDNA 2410129E14 gene | -51.63 | 0.009 |
| 1427351_s_at | immunoglobulin heavy chain 6 (heavy chain of IgM) | -14.92 | 0.038 |
| 1427356_at | RIKEN cDNA 2310031A18 gene | -2.76 | 0.04 |
| 1427539_a_at | ZW10 interactor | -19.45 | 0.023 |
| 1427817_at | Mouse endogenous mammary tumor virus (MMTV) RNA | -4.73 | 0.023 |
| 1427820_at | Mus musculus, clone IMAGE:3983821, mRNA, partial cds | -12.6 | 0.009 |
| 1427897_s_at | RIKEN cDNA 2400003N08 gene | -2.35 | 0.024 |
| 1427912_at | carbonyl reductase 3 | -10.49 | 0.023 |
| 1428015_at | DNA segment, Chr 12, Wayne State University 118, expressed | -3.22 | 0.041 |
| 1428191_s_at | DNA segment, Chr 14, ERATO Doi 209, expressed | -3.2 | 0.035 |
| 1428217_at | RIKEN cDNA 1600012H06 gene | -32.68 | 0.049 |
| 1428314_at | PEST-containing nuclear protein | -22.56 | 0.004 |
| 1428333_at | RIKEN cDNA 6530401D17 gene | -9.69 | 0.036 |
| 1428580_at | biliverdin reductase A | -7.33 | 0.031 |
| 1428737_s_at | RIKEN cDNA 9130427A09 gene | -2.62 | 0.035 |
| 1428780_at | threonine aldolase 1 | -5.77 | 0.043 |
| 1429186_a_at | cytidine and dCMP deaminase domain containing 1 | -4.31 | 0.004 |
| 1429247_at | annexin A6 | -6.9 | 0.029 |
| 1429253_at | zinc finger protein 262 | -6.91 | 0.026 |
| 1429265_a_at | ring finger protein 130 | -22.42 | 0.026 |
| 1429783_at | PDZ and LIM domain 5 | -22.18 | 0.034 |
| 1429848_at | poliovirus receptor | -2.91 | 0.043 |
| 1430021_a_at | ubiquitin-like 1 (sentrin) activating enzyme E1A | -3.88 | 0.034 |
| 1430385_a_at | galactosidase, beta 1-like | -5.67 | 0.005 |
| 1430610_at | mitochondrial ribosomal protein L38 | -2.57 | 0.005 |
| 1431105_a_at | transmembrane protein 33 | -3.39 | 0.017 |
| 1431960_at | WW domain-containing oxidoreductase | -5.72 | 0.016 |
| 1432164_a_at | glycine cleavage system protein H (aminomethyl carrier) | -6.58 | 0.034 |
| 1432255_at | microtubule associated serine/threonine kinase 1 | -8.22 | 0.05 |
| 1432281_a_at | integrin beta 6 | -2.58 | 0.042 |
| 1433029_at | potassium inwardly-rectifying channel, subfamily J, member 9 | -3.75 | 0.004 |
| 1433482_a_at | far upstream element (FUSE) binding protein 1 | -18.18 | 0.05 |
| 1433488_x_at | glucosamine (N-acetyl)-6-sulfatase | -8.33 | 0.035 |
| 1433489_s_at | fibroblast growth factor receptor 2 | -2.47 | 0.017 |
| 1433514_at | ethanolamine kinase 1 | -4.56 | 0.049 |
| 1433546_at | glucosamine (N-acetyl)-6-sulfatase | -12.94 | 0.043 |
| 1433718_a_at | chromobox homolog 1 (Drosophila HP1 beta) | -7.23 | 0.048 |
| 1433757_a_at | nischarin | -2.6 | 0.012 |
| 1433784_at | expressed sequence AI265322 | -44.2 | 0.043 |
| 1433904_at | gametogenetin binding protein 1 | -5.22 | 0.027 |
| 1433913_at | expressed sequence C80913 | -9.8 | 0.038 |
| 1433984_a_at | malate dehydrogenase 2, NAD (mitochondrial) | -2.35 | 0.049 |
| 1434005_at | RIKEN cDNA 6030432P03 gene | -32.1 | 0.037 |
| 1434017_at | zinc and ring finger 2 | -2.47 | 0.038 |
| 1434019_at | PDGFA associated protein 1 | -3.24 | 0.032 |
| 1434043_a_at | AV286809 | -3.86 | 0.024 |
| 1434392_at | ubiquitin specific protease 34 | -7.46 | 0.013 |
| 1434472_at | dual specificity phosphatase 3 (vaccinia virus phosphatase VH1-related) | -3.42 | 0.036 |
| 1434511_at | phosphorylase kinase beta | -19.6 | 0.007 |
| 1434659_at | RIKEN cDNA 5830411G16 gene | -20.36 | 0.043 |
| 1434705_at | C-terminal binding protein 2 | -28.28 | 0.037 |
| 1434914_at | RAB6B, member RAS oncogene family | -12.95 | 0.042 |
| 1435011_x_at | aldo-keto reductase family 1, member A4 (aldehyde reductase) | -2.59 | 0.013 |
| 1435191_at | corneodesmosin | -3.12 | 0.035 |
| 1435326_at | expressed sequence AW112037 | -29.41 | 0.001 |
| 1435360_at | zinc finger protein 651 | -2.97 | 0.027 |
| 1435683_a_at | ATP-binding cassette, sub-family C (CFTR/MRP), member 5 | -2.7 | 0.015 |
| 1435734_x_at | dihydrouridine synthase 1-like (S. cerevisiae) | -8.26 | 0.011 |
| 1436188_a_at | N-myc downstream regulated gene 4 | -9.91 | 0.043 |
| 1436277_at | 603356092F1 | -4.52 | 0.022 |
| 1436487_x_at | weakly similar to XP_231162.2 PREDICTED | -61.45 | 0.006 |
| 1436549_a_at | Heterogeneous nuclear ribonucleoprotein A1 | -7.53 | 0.016 |
| 1436570_at | Transcribed locus | -10.22 | 0.036 |
| 1436900_x_at | leptin receptor overlapping transcript | -47.36 | 0.033 |
| 1436917_s_at | G-protein signalling modulator 1 (AGS3-like, C. elegans) | -14.39 | 0.029 |
| 1436947_a_at | thioredoxin-like 1 | -2.98 | 0.019 |
| 1436956_at | Beta-site APP cleaving enzyme 1 | -3.61 | 0.028 |
| 1436965_at | elastin microfibril interfacer 3 | -4.25 | 0.01 |
| 1437026_at | cDNA sequence BC057893 | -11.75 | 0.028 |
| 1437132_x_at | neural precursor cell expressed, developmentally down-regulated gene 9 | -7.18 | 0.034 |
| 1437192_x_at | voltage-dependent anion channel 1 | -3.38 | 0.003 |
| 1437194_x_at | RIKEN cDNA 1200011O22 gene | -7.32 | 0.048 |
| 1437308_s_at | coagulation factor II (thrombin) receptor | -80.65 | 0.048 |
| 1437379_x_at | TNF receptor-associated protein 1 | -7.33 | 0.004 |
| 1437456_x_at | YTH domain family 1 | -6.68 | 0.038 |
| 1437526_x_at | heterogeneous nuclear ribonucleoprotein R | -3.02 | 0.033 |
| 1437585_x_at | zinc finger protein 161 | -6.22 | 0.001 |
| 1437621_x_at | 3-phosphoglycerate dehydrogenase | -2.72 | 0.041 |
| 1437716_x_at | kinesin family member 22 | -23.82 | 0.02 |
| 1437722_x_at | Poly(rC) binding protein 3 | -2.79 | 0.03 |
| 1437723_s_at | Der1-like domain family, member 1 | -7.69 | 0.025 |
| 1437804_at | Insulin-like growth factor binding protein 7 | -2.05 | 0.004 |
| 1437807_x_at | catenin (cadherin associated protein), alpha 1 | -12.37 | 0.04 |
| 1437845_x_at | protein O-fucosyltransferase 2 | -3.87 | 0.018 |
| 1437882_s_at | RIKEN cDNA 3110048L19 gene | -2.64 | 0.048 |
| 1437924_at | Son cell proliferation protein | -109.72 | 0.036 |
| 1437947_x_at | voltage-dependent anion channel 1 | -3.84 | 0.025 |
| 1438318_x_at | RIKEN cDNA 1500001L15 gene | -14.52 | 0.033 |
| 1438376_s_at | tripartite motif protein 27 | -9.05 | 0.015 |
| 1438379_x_at | RIKEN cDNA 2310007F21 gene | -26.03 | 0.012 |
| 1438391_x_at | hydroxyacyl-Coenzyme A dehydrogenase type II | -5.09 | 0.039 |
| 1438463_x_at | zinc finger, DHHC domain containing 6 | -5.15 | 0.027 |
| 1438562_a_at | protein tyrosine phosphatase, non-receptor type 2 | -48.16 | 0.01 |
| 1438645_x_at | beta-site APP-cleaving enzyme 2 | -5.9 | 0.025 |
| 1438761_a_at | ornithine decarboxylase, structural 1 | -3.8 | 0.042 |
| 1438832_x_at | DEAH (Asp-Glu-Ala-His) box polypeptide 30 | -8.87 | 0.021 |
| 1438941_x_at | adenosine monophosphate deaminase 2 (isoform L) | -29.17 | 0.024 |
| 1438957_x_at | CDP-diacylglycerol synthase (phosphatidate cytidylyltransferase) 2 | -12.32 | 0.03 |
| 1438969_x_at | DEAH (Asp-Glu-Ala-His) box polypeptide 30 | -16.78 | 0.004 |
| 1439103_at | hyperparathyroidism 2 homolog (human) | -49.78 | 0.031 |
| 1439191_at | Follistatin-like 1 | -13.26 | 0.014 |
| 1439389_s_at | myeloid-associated differentiation marker | -4.2 | 0.019 |
| 1439405_x_at | RIKEN cDNA 1700051C09 gene | -3.38 | 0.022 |
| 1439413_x_at | mortality factor 4 like 2 | -3.77 | 0.012 |
| 1439426_x_at | P lysozyme structural | -11.4 | 0.026 |
| 1439432_x_at | mortality factor 4 like 2 | -3.47 | 0.018 |
| 1439466_s_at | expressed sequence C77604 | -2.08 | 0.018 |
| 1443762_s_at | myotubularin related protein 13 | -7.79 | 0.041 |
| 1447494_at | DNA segment, Chr 7, Brigham & Women's Genetics 0826 expressed | -2.25 | 0.045 |
| 1447858_x_at | interleukin 4 receptor, alpha | -4.01 | 0.033 |
| 1448182_a_at | CD24a antigen | -4.08 | 0.016 |
| 1448249_at | glycerol-3-phosphate dehydrogenase 1 (soluble) | -4.52 | 0.033 |
| 1448263_a_at | CNDP dipeptidase 2 (metallopeptidase M20 family) | -25.59 | 0.033 |
| 1448334_a_at | cyclin I | -5.22 | 0.02 |
| 1448347_a_at | GPI-anchored membrane protein 1 | -14.14 | 0.003 |
| 1448383_at | matrix metalloproteinase 14 (membrane-inserted) | -10.05 | 0.036 |
| 1448405_a_at | CREBBP/EP300 inhibitory protein 1 | -2.65 | 0.04 |
| 1448410_at | ubiquitination factor E4B, UFD2 homolog (S. cerevisiae) | -59.43 | 0.035 |
| 1448475_at | olfactomedin-like 3 | -7.04 | 0.041 |
| 1448499_a_at | epoxide hydrolase 2, cytoplasmic | -2.8 | 0.011 |
| 1448508_at | Traf3 interacting protein 2 | -5.23 | 0.033 |
| 1448551_a_at | tripartite motif protein 2 | -10.86 | 0.005 |
| 1448568_a_at | solute carrier family 20, member 1 | -4.12 | 0.018 |
| 1448598_at | matrix metalloproteinase 17 | -2.09 | 0.022 |
| 1448642_at | poly(rC) binding protein 1 | -11.34 | 0.033 |
| 1448689_at | related RAS viral (r-ras) oncogene homolog 2 | -4.69 | 0.037 |
| 1448698_at | cyclin D1 | -5.63 | 0.038 |
| 1448705_at | zinc finger protein 297 | -3.11 | 0.035 |
| 1448778_at | splicing factor, arginine/serine-rich 4 (SRp75) | -2.07 | 0.029 |
| 1448873_at | occludin | -5.6 | 0.014 |
| 1448979_at | muted | -26.13 | 0.016 |
| 1449040_a_at | selenophosphate synthetase 2 | -2.43 | 0.026 |
| 1449049_at | toll-like receptor 1 | -21.97 | 0.036 |
| 1449200_at | H3127F09-3 | -45.3 | 0.044 |
| 1449233_at | basic helix-loop-helix domain containing, class B, 8 | -6.15 | 0.043 |
| 1449239_at | RIKEN cDNA 1700045I19 gene | -4.63 | 0.032 |
| 1449263_at | ubiquitin-fold modifier 1 | -3.59 | 0.046 |
| 1449295_at | transcriptional regulator protein | -4.85 | 0.019 |
| 1449346_s_at | RIO kinase 1 (yeast) | -9.89 | 0.039 |
| 1449360_at | colony stimulating factor 2 receptor, beta 2 | -11.72 | 0.016 |
| 1449491_at | caspase recruitment domain family, member 10 | -7.63 | 0.043 |
| 1449587_a_at | mucin 10, submandibular gland salivary mucin | -3.86 | 0.038 |
| 1449597_at | similar to odorant binding protein Ib | -17.2 | 0.049 |
| 1449640_at | toll-like receptor 7 | -2.3 | 0.016 |
| 1449700_at | Immunoglobulin (CD79A) binding protein 1 | -6.23 | 0.041 |
| 1449733_s_at | seven in absentia 1A | -3.37 | 0.017 |
| 1449739_at | Phosphatidylserine synthase 1 | -10.85 | 0.035 |
| 1450074_at | kinesin family member 3B | -3.69 | 0.046 |
| 1450117_at | transcription factor 3 | -3.19 | 0.022 |
| 1450187_a_at | galactose-1-phosphate uridyl transferase | -3.66 | 0.022 |
| 1450423_s_at | brix domain containing 1 | -21.25 | 0.03 |
| 1450443_at | polypyrimidine tract binding protein 1 | -2.52 | 0.036 |
| 1450618_a_at | small proline-rich protein 2A | -2.41 | 0.031 |
| 1450711_at | bromodomain containing 4 | -4.54 | 0.047 |
| 1450799_at | adenylate cyclase activating polypeptide 1 receptor 1 | -7.72 | 0.023 |
| 1450808_at | formyl peptide receptor 1 | -3.81 | 0.041 |
| 1450941_at | Mm.14744 | -2.99 | 0.047 |
| 1450980_at | GTP binding protein 3 | -21.64 | 0.046 |
| 1451010_at | RIKEN cDNA 1500002M01 gene | -2.78 | 0.04 |
| 1451025_at | ADP-ribosylation factor-like 1 | -3.84 | 0.047 |
| 1451132_at | pre-B-cell leukemia transcription factor interacting protein 1 | -2.18 | 0.047 |
| 1451161_a_at | EGF-like module containing, mucin-like, hormone receptor-like sequence 1 | -16.83 | 0.016 |
| 1451304_at | RIKEN cDNA 2310076O21 gene | -3.5 | 0.042 |
| 1451448_a_at | RIKEN cDNA 1110005A03 gene | -5.53 | 0.026 |
| 1451493_at | Nedd4 family interacting protein 1 | -8.74 | 0.005 |
| 1451495_at | WW domain containing adaptor with coiled-coil | -5.95 | 0.046 |
| 1451508_at | RIKEN cDNA 1700108L22 gene | -21.31 | 0.015 |
| 1451678_at | RIKEN cDNA 4430402O11 gene | -2.53 | 0.037 |
| 1451938_a_at | syntrophin, basic 1 | -3.28 | 0.035 |
| 1451971_at | cullin 4A | -5.61 | 0.032 |
| 1452039_a_at | Brca1 associated protein 1 | -3.62 | 0.041 |
| 1452116_s_at | activating transcription factor 2 | -6.17 | 0.031 |
| 1452202_at | phosphodiesterase 2A, cGMP-stimulated | -5.67 | 0.039 |
| 1452215_at | RIKEN cDNA 9130401M01 gene | -3.03 | 0.038 |
| 1452286_at | RIKEN cDNA 5033405K12 gene | -24.47 | 0.05 |
| 1452288_at | expressed sequence BB128963 | -8.4 | 0.016 |
| 1452329_at | pleckstrin homology domain containing, family N member 1 | -2.18 | 0.041 |
| 1452334_at | centromere autoantigen F | -3.79 | 0.016 |
| 1452341_at | enoyl Coenzyme A hydratase, short chain, 1, mitochondrial | -3.56 | 0.047 |
| 1452349_x_at | interferon activated gene 205 | -10.16 | 0.022 |
| 1452415_at | actinin, alpha 1 | -3.66 | 0.014 |
| 1452547_s_at | histocompatibility 2, D region locus 1 | -2.81 | 0.046 |
| 1452679_at | RIKEN cDNA 2410129E14 gene | -12.38 | 0.011 |
| 1452709_at | polymerase (DNA-directed), delta interacting protein 3 | -7.68 | 0.005 |
| 1452713_a_at | WD repeat domain 57 (U5 snRNP specific) | -4.41 | 0.009 |
| 1452754_at | RIKEN cDNA 5730592L21 gene | -21.03 | 0.031 |
| 1452770_at | vitamin K epoxide reductase complex, subunit 1 | -15.02 | 0.03 |
| 1452839_at | RIKEN cDNA 2410012M04 gene | -6.3 | 0.033 |
| 1452900_at | DiGeorge syndrome critical region gene 6 | -5.25 | 0.037 |
| 1453089_at | RIKEN cDNA 3110079O15 gene | -3.06 | 0.032 |
| 1453198_at | zinc finger protein 422, related sequence 1 | -34.64 | 0.035 |
| 1453206_at | acyl-Coenzyme A dehydrogenase family, member 9 | -4.18 | 0.047 |
| 1453281_at | Phosphatidylinositol 3-kinase catalytic delta polypeptide | -2.08 | 0.043 |
| 1453851_a_at | growth arrest and DNA-damage-inducible 45 gamma | -7.84 | 0.04 |
| 1454145_at | phospholipase A2, group IB, pancreas, receptor | -3.4 | 0.044 |
| 1454221_a_at | kinesin family member 2C | -2.23 | 0.035 |
| 1454639_x_at | ribosomal protein L41 | -25.82 | 0.042 |
| 1454669_at | RIKEN cDNA 5730466P16 gene | -4.27 | 0.01 |
| 1454708_at | actin-binding LIM protein 1 | -2.66 | 0.049 |
| 1454785_at | dual specificity phosphatase 11 | -4.33 | 0.024 |
| 1454789_x_at | RIKEN cDNA 2610031L17 gene | -2.96 | 0.045 |
| 1454893_at | RIKEN cDNA 1110013L07 gene | -4.51 | 0.035 |
| 1455002_at | protein tyrosine phosphatase 4a1 | -7.13 | 0.041 |
| 1455012_s_at | tripartite motif protein 37 | -14.44 | 0.026 |
| 1455479_a_at | ubiquitin-conjugating enzyme E2D 3 (UBC4/5 homolog, yeast) | -8.5 | 0.044 |
| 1455534_s_at | oxysterol binding protein-like 11 | -7.23 | 0.011 |
| 1455905_at | RIKEN cDNA 2610507B11 gene | -8.17 | 0.019 |
| 1456011_x_at | acetyl-Coenzyme A acyltransferase 1 | -5.11 | 0.043 |
| 1456014_s_at | cDNA sequence BC032204 | -7.47 | 0.032 |
| 1456028_x_at | Myristoylated alanine rich protein kinase C substrate | -2.75 | 0.03 |
| 1456037_x_at | prolactin regulatory element binding | -2.88 | 0.028 |
| 1456055_x_at | polymerase (DNA directed), delta 1, catalytic subunit | -6.45 | 0.05 |
| 1456081_a_at | acetoacetyl-CoA synthetase | -13.79 | 0.01 |
| 1456086_x_at | polyglutamine binding protein 1 | -25.45 | 0.024 |
| 1456190_a_at | cDNA sequence BC031140 | -3.28 | 0.024 |
| 1456213_x_at | glutaminyl-tRNA synthetase | -3.41 | 0.029 |
| 1456228_x_at | myelin basic protein | -3.66 | 0.021 |
| 1456240_x_at | cell division cycle associated 4 | -2.6 | 0.016 |
| 1456279_a_at | B-cell receptor-associated protein 31 | -12.05 | 0.013 |
| 1456360_at | RIKEN cDNA 1700022C02 gene | -6.96 | 0.016 |
| 1456375_x_at | tripartite motif protein 27 | -6.79 | 0.026 |
| 1456388_at | ATPase, class VI, type 11A | -10.2 | 0.004 |
| 1456434_x_at | heat shock 27kDa protein 8 | -4.55 | 0.029 |
| 1456470_x_at | RIKEN cDNA 4930542G03 gene | -2.91 | 0.016 |
| 1456511_x_at | ES cell-expressed Ras | -6.39 | 0.025 |
| 1456530_x_at | elongation of very long chain fatty acids | -10.11 | 0.042 |
| 1456726_x_at | glutaminyl-tRNA synthetase | -3.31 | 0.037 |
| 1456737_x_at | acetyl-Coenzyme A acyltransferase 1 | -5.16 | 0.007 |
| 1456743_x_at | mortality factor 4 like 2 | -2.96 | 0.039 |
| 1458414_at | DNA segment, Chr 2, ERATO Doi 93, expressed | -3.78 | 0.046 |
| 1459092_at | solute carrier organic anion transporter family, member 6c1 | -6.28 | 0.043 |
| 1460194_at | phytanoyl-CoA hydroxylase | -6.17 | 0.047 |
| 1460221_at | prostaglandin E synthase 3 (cytosolic) | -3.16 | 0.019 |
| 1460382_at | cDNA sequence BC020535 | -5.12 | 0.03 |
| 1460444_at | arrestin, beta 1 | -4.02 | 0.02 |
| 1460648_at | nuclear receptor subfamily 2, group F, member 6 | -4.16 | 0.03 |
| 1460704_at | radical fringe gene homolog (Drosophila) | -4.38 | 0.033 |
| 1460709_a_at | HLA-B associated transcript 5 | -2.6 | 0.021 |
| 1420313_x_at | Mm.170660.2 | -5.12 | 0.045 |
| 1428136_at | secreted frizzled-related sequence protein 1 | -29.63 | 0.048 |
| 1428185_at | hypothetical protein 6530404F10Rik | -6.35 | 0.01 |
| 1428228_at | phosphoglucomutase 3 | -5.2 | 0.033 |
| 1428564_at | zinc finger protein 579 | -36.96 | 0.023 |
| 1428795_at | RIKEN cDNA 1110021L09 gene | -22.31 | 0.015 |
| 1428892_at | peptidylprolyl isomerase (cyclophilin)-like 1 | -5.57 | 0.019 |
| 1428914_at | RIKEN cDNA 2310014D11 gene | -3.1 | 0.038 |
| 1428947_at | RIKEN cDNA 2010001M09 gene | -4.36 | 0.014 |
| 1428998_at | PHD finger protein 3 | -22.16 | 0.006 |
| 1429055_at | RIKEN cDNA 4930506M07 gene | -3.2 | 0.034 |
| 1429151_at | WD repeat domain 68 | -12.46 | 0.049 |
| 1429256_at | GTL2, imprinted maternally expressed untranslated mRNA | -3.29 | 0.048 |
| 1429611_at | RIKEN cDNA 1700034E13 gene | -4.26 | 0.039 |
| 1429742_at | chromosome condensation 1-like | -46.75 | 0.031 |
| 1429747_at | RIKEN cDNA 1700009N14 gene | -2.29 | 0.023 |
| 1429812_at | RIKEN cDNA 2610002D18 gene | -3.5 | 0.008 |
| 1429860_at | RIKEN cDNA 5730590G19 gene | -2.57 | 0.016 |
| 1429885_at | RIKEN cDNA 4930431P19 gene | -3.15 | 0.036 |
| 1429942_at | glucocorticoid modulatory element binding protein 1 | -5.63 | 0.022 |
| 1429983_at | RIKEN cDNA 2010002M09 gene | -2.2 | 0.045 |
| 1430039_at | CDK5 regulatory subunit associated protein 1-like 1 | -19.18 | 0.031 |
| 1430071_at | brain protein 44 | -14.95 | 0.048 |
| 1430253_at | RIKEN cDNA 2900006B11 gene | -3.83 | 0.021 |
| 1430442_at | nitric oxide synthase 1 (neuronal) adaptor protein | -3.01 | 0.039 |
| 1430472_at | armadillo repeat containing 1 | -8.45 | 0.042 |
| 1430561_at | DnaJ (Hsp40) homolog, subfamily B, member 14 | -8.08 | 0.013 |
| 1430691_at | RIKEN cDNA 4632411P08 gene | -5.36 | 0.019 |
| 1430991_at | RIKEN cDNA 1810014B01 gene | -4.9 | 0.01 |
| 1431173_at | RIKEN cDNA A930008G19 gene | -11.6 | 0.023 |
| 1431376_at | WD repeat domain 62 | -2.58 | 0.031 |
| 1431424_at | RIKEN cDNA 2810055G20 gene | -8.59 | 0.009 |
| 1431628_at | RIKEN cDNA 4930435H24 gene | -8.8 | 0.009 |
| 1432232_at | REST corepressor 3 | -4.38 | 0.039 |
| 1432365_a_at | RIKEN cDNA 4930556L07 gene | -2.99 | 0.025 |
| 1432448_at | cerebral endothelial cell adhesion molecule 1 | -7.24 | 0.036 |
| 1432548_at | poly (ADP-ribose) polymerase family, member 14 | -6.84 | 0.038 |
| 1432650_at | RIKEN cDNA 2510019K15 gene | -5.35 | 0.015 |
| 1433023_at | RIKEN cDNA 2310068G24 gene | -8.1 | 0.001 |
| 1433061_at | RIKEN cDNA 4933423L19 gene | -3.97 | 0.023 |
| 1433101_at | RIKEN cDNA 9030419F21 gene | -39.45 | 0.029 |
| 1433189_at | RIKEN cDNA 4933433N18 gene | -2.9 | 0.006 |
| 1433551_at | expressed sequence AI427515 | -6.36 | 0.002 |
| 1433571_at | RIKEN cDNA A130038L21 gene | -29.77 | 0.022 |
| 1433601_at | Mm.25215 | -2.7 | 0.031 |
| 1433682_at | Rho guanine nucleotide exchange factor (GEF) 17 | -2.45 | 0.034 |
| 1433722_at | A kinase (PRKA) anchor protein 13 | -15.58 | 0.039 |
| 1433889_at | SRY-box containing gene 9 | -20.51 | 0.026 |
| 1433979_at | RNA binding motif, single stranded interacting protein 2 | -3.1 | 0.043 |
| 1434013_at | actin binding LIM protein family, member 3 | -6.45 | 0.001 |
| 1434132_at | RIKEN cDNA E430025E21 gene | -6.02 | 0.014 |
| 1434178_at | myeloid/lymphoid or mixed-lineage leukemia 3 | -68.58 | 0.043 |
| 1434232_a_at | C630029D24 | -21.31 | 0.032 |
| 1434349_at | valyl-tRNA synthetase 2-like | -2.58 | 0.015 |
| 1434351_at | nucleoporin 214 | -3.74 | 0.028 |
| 1434385_at | target of myb1-like 2 (chicken) | -3.28 | 0.036 |
| 1434577_at | cDNA sequence BC052040 | -90.03 | 0.015 |
| 1434656_at | RIKEN cDNA B230339M05 gene | -4 | 0.04 |
| 1434860_at | NMDA receptor-regulated gene 3 | -28.64 | 0.012 |
| 1434895_s_at | protein phosphatase 1, regulatory (inhibitor) subunit 13B | -7.22 | 0.036 |
| 1434928_at | growth arrest-specific 2 like 1 | -5.48 | 0.013 |
| 1434961_at | ankyrin repeat and SOCS box-containing protein 1 | -18.24 | 0.016 |
| 1434973_at | carbonic anhydrase 7 | -4.22 | 0.043 |
| 1435020_at | Serologically defined colon cancer antigen 1 | -13.98 | 0.02 |
| 1435084_at | RIKEN cDNA C730049O14 gene | -3.55 | 0.022 |
| 1435135_at | arylacetamide deacetylase-like 1 | -3.94 | 0.033 |
| 1435197_at | POU domain, class 3, transcription factor 3 | -3.97 | 0.005 |
| 1435233_at | nuclear receptor coactivator 2 | -72.6 | 0.026 |
| 1435293_at | RIKEN cDNA 2900022I03 gene | -2.3 | 0.043 |
| 1435295_at | DNA segment, Chr 9, ERATO Doi 809, expressed | -32.58 | 0.034 |
| 1435319_at | RIKEN cDNA 1500005N04 gene | -3.78 | 0.033 |
| 1435406_at | coiled-coil alpha-helical rod protein 1 | -2.46 | 0.043 |
| 1435422_at | RIKEN cDNA 4933433P14 gene | -10.22 | 0.042 |
| 1435423_x_at | RIKEN cDNA 4933433P14 gene | -3.64 | 0.011 |
| 1435436_at | Transcribed locus | -4.12 | 0.039 |
| 1435445_at | cyclin T2 | -33.71 | 0.015 |
| 1435595_at | RIKEN cDNA 1810011O10 gene | -43.27 | 0.039 |
| 1435679_at | optineurin | -4.31 | 0.017 |
| 1435688_at | Adult male testis cDNA | -3.55 | 0.032 |
| 1435703_at | RIKEN cDNA 2810457I06 gene | -3.02 | 0.039 |
| 1435749_at | guanine deaminase | -4.6 | 0.027 |
| 1435825_at | Activin A receptor, type II-like 1 | -2.68 | 0.023 |
| 1435836_at | Pyruvate dehydrogenase kinase, isoenzyme 1 | -39.91 | 0.026 |
| 1435842_at | RIKEN cDNA 1110038O08 gene | -4.21 | 0.011 |
| 1436033_at | cDNA sequence BC031353 | -8.72 | 0.016 |
| 1436141_at | RIKEN cDNA 2610510L01 gene | -5.02 | 0.019 |
| 1436238_at | leucine-rich repeat LGI family, member 3 | -4 | 0.027 |
| 1436294_at | ankyrin repeat domain 29 | -8.7 | 0.005 |
| 1436324_at | START domain containing 9 | -22.24 | 0.017 |
| 1436328_at | RNA (guanine-7-) methyltransferase | -20.88 | 0.014 |
| 1436345_at | RIKEN cDNA 5730559C18 gene | -2.73 | 0.017 |
| 1436424_at | RIKEN cDNA 1600020E01 gene | -4.98 | 0.033 |
| 1436436_at | cornichon homolog 4 (Drosophila) | -4.59 | 0.043 |
| 1436469_at | Bromodomain containing 7 | -7.09 | 0.048 |
| 1436523_s_at | RIKEN cDNA 1810022K09 gene | -5.4 | 0.035 |
| 1436606_at | Transcribed locus | -10.11 | 0.01 |
| 1436618_at | sideroflexin 5 | -8.55 | 0.034 |
| 1436813_x_at | KH-type splicing regulatory protein | -6.82 | 0.008 |
| 1436850_at | cellular repressor of E1A-stimulated genes 2 | -4.11 | 0.036 |
| 1436977_at | Transcribed locus | -5.92 | 0.029 |
| 1437056_x_at | cysteine-rich secretory protein LCCL domain containing 2 | -7.05 | 0.018 |
| 1437070_at | CDC14 cell division cycle 14 homolog B (S. cerevisiae) | -25.35 | 0.006 |
| 1437087_at | MKIAA0231 protein | -6.27 | 0.037 |
| 1437224_at | reticulon 4 | -3.57 | 0.043 |
| 1437353_at | cDNA sequence BC035291 | -7.3 | 0.001 |
| 1437361_at | Similar to hypothetical protein A430060F13 | -5.73 | 0.02 |
| 1437366_at | expressed sequence AI608492 | -7.57 | 8E-04 |
| 1437385_at | Transcribed locus | -17.8 | 0.042 |
| 1437435_at | RIKEN cDNA 1700061G19 gene | -4.55 | 0.046 |
| 1437436_s_at | G protein-coupled receptor kinase 6 | -8.56 | 7E-04 |
| 1437524_x_at | coronin 7 | -4.52 | 0.038 |
| 1437542_at | RIKEN cDNA A730095J18 gene | -22.03 | 0.013 |
| 1437749_s_at | mitochondrial ribosomal protein L9 | -2.6 | 0.045 |
| 1437774_at | RIKEN cDNA 1700020I14 gene | -5.47 | 0.035 |
| 1438363_at | similar to KIAA1183 protein | -7.55 | 0.007 |
| 1438420_at | RNA-binding region (RNP1, RRM) containing 2 | -6.12 | 0.025 |
| 1438505_s_at | ribonuclease III, nuclear | -9.3 | 0.048 |
| 1438576_x_at | RIKEN cDNA 2900056M20 gene | -2.29 | 0.049 |
| 1438724_at | oxysterol binding protein-like 3 | -6.2 | 0.039 |
| 1438727_at | WD repeat domain 32 | -3.37 | 0.014 |
| 1438783_at | Transmembrane, prostate androgen induced RNA | -8.35 | 0.048 |
| 1438967_x_at | anti-Mullerian hormone type 2 receptor | -7.45 | 0.025 |
| 1439043_at | RIKEN cDNA 1500010G04 gene | -3.07 | 0.035 |
| 1439082_at | DEAD (Asp-Glu-Ala-Asp) box polypeptide 50 | -13.77 | 0.027 |
| 1439143_at | hypothetical protein 9330167E06 | -7.28 | 0.003 |
| 1439189_at | RIKEN cDNA D630023B12 gene | -27.79 | 0.009 |
| 1439220_at | Ankyrin 3, epithelial | -3.85 | 0.016 |
| 1439276_at | Adenosine deaminase, RNA-specific | -2.65 | 0.048 |
| 1439293_at | RIKEN cDNA C130047D21 gene | -26.91 | 2E-04 |
| 1439565_at | Expressed sequence AW492303 | -17.74 | 0.013 |
| 1439690_at | RIKEN cDNA 5133401N09 gene | -5.77 | 0.035 |
| 1439698_at | Zinc finger protein (C2H2 type) 276 | -3.89 | 0.044 |
| 1439939_at | RIKEN cDNA E030042N06 gene | -3.77 | 0.009 |
| 1440124_at | RIKEN cDNA B230334C09 gene | -5.72 | 0.027 |
| 1440144_x_at | hypothetical protein C330046E03 | -5.7 | 0.022 |
| 1440147_at | leucine-rich repeat LGI family, member 2 | -5.51 | 0.005 |
| 1440284_at | Transcribed locus | -4.44 | 0.014 |
| 1440337_at | Mm.30827.1 | -2.32 | 0.04 |
| 1440563_at | Fanconi anemia, complementation group C | -3.87 | 0.019 |
| 1440594_at | Protein phosphatase 1, catalytic subunit, gamma isoform | -3.04 | 0.015 |
| 1440675_at | Transcribed locus | -3.54 | 0.04 |
| 1440690_at | Mm.208145 | -44.05 | 0.019 |
| 1440816_x_at | DEAD (Asp-Glu-Ala-Asp) box polypeptide 1 | -10.8 | 0.007 |
| 1440940_at | Calcium channel, voltage-dependent, beta 1 subunit | -4.31 | 0.038 |
| 1440952_at | MAD homolog 7 (Drosophila) | -2.49 | 0.049 |
| 1440992_at | RIKEN cDNA 3110052M02 gene | -3.39 | 0.041 |
| 1441012_at | Huntingtin interacting protein 2 | -3.41 | 0.043 |
| 1441259_s_at | WD repeat domain 10 | -4.68 | 0.005 |
| 1441273_at | Mm.32983 | -2.69 | 0.039 |
| 1441445_at | period homolog 3 (Drosophila) | -13.09 | 0.028 |
| 1441493_at | Rab6 interacting protein 2 | -8.93 | 0.02 |
| 1441558_at | RIKEN cDNA D230044B12 gene | -14.39 | 0.027 |
| 1441639_at | zinc finger, CCHC domain containing 8 | -12.98 | 0.044 |
| 1441687_at | Wingless-related MMTV integration site 4 | -3.76 | 0.014 |
| 1441794_at | RIKEN cDNA 4933437K13 gene | -2.24 | 0.04 |
| 1441880_x_at | hypothetical protein MGC30332 | -9.72 | 0.015 |
| 1441898_at | Transcribed locus | -4.56 | 0.019 |
| 1441906_x_at | Synapse associated protein 1 | -7.5 | 6E-04 |
| 1441926_x_at | transmembrane inner ear | -20.3 | 0.041 |
| 1441927_at | Synaptotagmin 7 | -10.58 | 3E-04 |
| 1441962_at | arachidonate 5-lipoxygenase | -3.65 | 0.026 |
| 1442334_at | similar to Hypothetical protein DJ1198H6.2 | -2.33 | 0.041 |
| 1442336_at | Mm.218032 | -14.1 | 0.032 |
| 1442365_at | Reticulon 3 | -15.8 | 0.048 |
| 1442427_at | RIKEN cDNA 9630026M06 gene | -11.96 | 0.04 |
| 1442434_at | DNA segment, Chr 8, ERATO Doi 82, expressed | -4.6 | 0.005 |
| 1442557_at | Synaptotagmin 1 | -3.21 | 0.045 |
| 1442605_at | RIKEN cDNA E030004N02 gene | -22.91 | 0.044 |
| 1442674_at | RIKEN cDNA 5330431N19 gene | -4.04 | 0.008 |
| 1442774_x_at | Transcribed locus | -5.62 | 0.019 |
| 1442792_x_at | RIKEN cDNA 4930471M23 gene | -6.47 | 0.008 |
| 1443066_at | Mm.134239.1 | -3.68 | 0.034 |
| 1443109_at | RIKEN cDNA 5830417C01 gene | -16.56 | 0.042 |
| 1443110_at | general transcription factor II E, polypeptide 1 | -4.15 | 0.015 |
| 1443158_at | Sex comb on midleg homolog 1 | -3.89 | 0.016 |
| 1443209_at | Histone 1, H2be | -17.03 | 0.039 |
| 1443343_at | Mm.212070 | -3.25 | 0.01 |
| 1443488_at | RIKEN cDNA 6430701C03 gene | -4.35 | 0.014 |
| 1443586_at | FIP1 like 1 (S. cerevisiae) | -8.59 | 0.026 |
| 1443748_x_at | RIKEN cDNA E130014H08 gene | -38.11 | 0.025 |
| 1443848_at | RIKEN cDNA C530047H08 gene | -19.24 | 0.049 |
| 1443870_at | ATP-binding cassette, sub-family C (CFTR/MRP), member 4 | -99.2 | 0.018 |
| 1443905_at | Transcribed locus | -5.08 | 0.027 |
| 1443917_at | RIKEN cDNA 0610007H07 gene | -7.26 | 0.02 |
| 1443947_at | LIM and senescent cell antigen-like domains 1 | -3.94 | 0.046 |
| 1443953_at | testis expressed gene 2 | -2.86 | 0.007 |
| 1443965_at | Transcribed locus | -3.93 | 0.036 |
| 1444013_at | RIKEN cDNA 5930412G12 gene | -3.4 | 0.015 |
| 1444045_at | RIKEN cDNA A430107O13 gene | -47.81 | 0.016 |
| 1444075_at | filamin A interacting protein 1 | -3.25 | 0.035 |
| 1444090_at | PML-RAR alpha-regulated adaptor molecule 1 | -5.2 | 0.016 |
| 1444100_at | RIKEN cDNA 1110003E01 gene | -9.22 | 0.034 |
| 1444152_at | RIKEN cDNA D230046B21 gene | -3.03 | 0.044 |
| 1444250_at | 16 days neonate heart cDNA, RIKEN full-length enriched library | -5.35 | 0.028 |
| 1444339_at | 0 day neonate kidney cDNA, RIKEN full-length enriched library | -9.91 | 0.02 |
| 1444429_at | RIKEN cDNA A930016D02 gene | -2.48 | 0.018 |
| 1444575_at | RIKEN cDNA 1500002O20 gene | -2.47 | 0.026 |
| 1444632_at | Transcribed locus | -9.56 | 0.044 |
| 1444657_at | Bcl3 binding protein | -5.52 | 0.02 |
| 1444777_at | Retinoic acid induced 14 | -2.48 | 0.023 |
| 1444785_at | Sloan-Kettering viral oncogene homolog | -5.02 | 0.003 |
| 1444838_at | hypothetical protein C630010N09 | -3.21 | 0.026 |
| 1444937_at | Mm.170660 | -2.81 | 0.03 |
| 1444955_at | DNA segment, Chr 6, ERATO Doi 469, expressed | -3.26 | 0.047 |
| 1445033_at | ATP-binding cassette, sub-family A (ABC1), member 16 | -5.08 | 0.033 |
| 1445211_at | Sex comb on midleg homolog 1 | -2.4 | 0.028 |
| 1445488_at | RAN binding protein 17 | -2.16 | 0.023 |
| 1445492_at | Microtubule-associated protein, RP/EB family, member 2 | -2.87 | 0.009 |
| 1445589_at | Solute carrier family 23 (nucleobase transporters), member 2 | -2.28 | 0.031 |
| 1445604_at | Transcribed locus | -63.88 | 0.032 |
| 1445626_at | Lectin, galactose binding, soluble 3 | -4.08 | 0.039 |
| 1445755_at | Mm.173427 | -7.03 | 0.047 |
| 1445761_at | DNA segment, Chr 4, ERATO Doi 628, expressed | -2.99 | 0.037 |
| 1445774_at | Potassium large conductance calcium-activated channel, subfamily M, alpha member 1 | -2.95 | 0.028 |
| 1445822_at | gene model 1285, (NCBI) | -3.51 | 0.015 |
| 1445875_at | RIKEN cDNA A230057D06 gene | -3.94 | 0.039 |
| 1445928_at | membrane-associated ring finger (C3HC4) 6 | -2.49 | 0.017 |
| 1446036_at | expressed sequence AU018728 | -3.72 | 0.042 |
| 1446125_at | Mm.173077 | -3.25 | 0.008 |
| 1446341_at | RIKEN cDNA 6030403N03 gene | -4.92 | 0.034 |
| 1446395_at | Roundabout homolog 2 (Drosophila) | -3.4 | 0.04 |
| 1446404_at | Mm.182758 | -4.85 | 0.04 |
| 1446412_at | WW domain-containing oxidoreductase | -4.02 | 0.012 |
| 1446452_at | Transcription factor 7-like 2, T-cell specific, HMG-box | -5.74 | 0.014 |
| 1446500_at | RIKEN cDNA A430081F14 gene | -4.4 | 0.046 |
| 1446688_at | Phosphatidylethanolamine binding protein | -4.65 | 0.045 |
| 1446691_at | Platelet-derived growth factor, D polypeptide | -5.03 | 0.03 |
| 1446712_at | neurotrophic tyrosine kinase, receptor, type 2 | -5.81 | 0.042 |
| 1446998_at | G protein-coupled receptor kinase 5 | -3.12 | 0.006 |
| 1447036_at | cell adhesion molecule with homology to L1CAM | -4.23 | 0.013 |
| 1447081_at | Mm.172797 | -50.69 | 0.045 |
| 1447087_at | Transcribed locus | -5.33 | 0.004 |
| 1447110_at | CDNA sequence BC031575 | -4.11 | 0.044 |
| 1447116_at | RIKEN cDNA 3110001A13 gene | -6.22 | 0.02 |
| 1447122_at | Proteasome (prosome, macropain) 26S subunit, non-ATPase, 1 | -17.33 | 0.033 |
| 1447246_at | 3 day neonate thymus cDNA, | -3.88 | 0.031 |
| 1447258_at | Mm.210555 | -5.31 | 0.028 |
| 1447410_at | Mm.210464 | -31.3 | 0.03 |
| 1447448_s_at | Mm.200959 | -5.03 | 0.007 |
| 1447610_at | transmembrane protein 34 | -3.37 | 0.037 |
| 1447691_x_at | Mm.157841 | -6.52 | 7E-04 |
| 1447693_s_at | neogenin | -30.98 | 0.019 |
| 1447757_x_at | inositol polyphosphate-5-phosphatase F | -33.62 | 0.004 |
| 1447776_x_at | RAB6, member RAS oncogene family | -7.89 | 0.04 |
| 1447804_x_at | CDNA sequence BC031781 | -15.64 | 0.001 |
| 1447842_x_at | Transcobalamin 2 | -2.59 | 0.047 |
| 1447874_x_at | sphingomyelin phosphodiesterase 1, acid lysosomal | -10.11 | 0.005 |
| 1447881_x_at | Mm.120368 | -17.54 | 0.007 |
| 1447917_x_at | N-terminal Asn amidase | -4 | 0.007 |
| 1447950_at | RIKEN cDNA A730011C13 gene | -2.83 | 0.023 |
| 1447979_at | RUN and TBC1 domain containing 2 | -18.68 | 0.047 |
| 1448049_at | Jumonji domain containing 1C | -4.67 | 0.044 |
| 1449791_x_at | RIKEN cDNA 9130023D20 gene | -6.19 | 0.021 |
| 1452915_at | protein kinase, cAMP dependent regulatory, type II alpha | -2.29 | 0.031 |
| 1453010_at | RIKEN cDNA 1700069O15 gene | -91.02 | 0.022 |
| 1453080_at | RIKEN cDNA 9130022K13 gene | -4.66 | 0.018 |
| 1453103_at | actin-binding LIM protein 1 | -24.38 | 0.042 |
| 1453179_at | phytoceramidase, alkaline | -2.25 | 0.031 |
| 1453272_at | RIKEN cDNA 4930579F01 gene | -5.13 | 0.004 |
| 1453452_at | RIKEN cDNA 4930517J16 gene | -3.74 | 0.025 |
| 1453565_at | NADH dehydrogenase (ubiquinone) 1, alpha/beta subcomplex, 1 | -3.58 | 0.02 |
| 1453664_at | RIKEN cDNA 9130017K11 gene | -4.05 | 0.015 |
| 1453665_at | RIKEN cDNA 4930529M08 gene | -3.35 | 0.042 |
| 1453667_at | RIKEN cDNA 1700065D16 gene | -7.43 | 0.011 |
| 1453745_at | RIKEN cDNA 2700038G22 gene | -3.65 | 0.038 |
| 1453933_at | RIKEN cDNA 4933435E02 gene | -4.51 | 0.048 |
| 1454071_at | lymphocyte antigen 6 complex, locus K | -3.79 | 0.038 |
| 1454102_at | RIKEN cDNA 5031425E22 gene | -2.11 | 0.015 |
| 1454238_a_at | RIKEN cDNA 1700010H22 gene | -4.24 | 0.045 |
| 1454243_at | RIKEN cDNA 2210420N10 gene | -62.32 | 0.048 |
| 1454324_at | RIKEN cDNA 4930487N04 gene | -11.11 | 0.013 |
| 1454393_at | RIKEN cDNA 2310047C04 gene | -12.19 | 0.029 |
| 1454570_at | RIKEN cDNA 5830432F11 gene | -4.9 | 0.014 |
| 1454745_at | Rho GTPase activating protein 29 | -3.83 | 0.037 |
| 1454748_at | RIKEN cDNA 9130210N20 gene | -5.18 | 0.01 |
| 1454797_at | cDNA sequence BC039161 | -2.26 | 0.046 |
| 1454919_at | expressed sequence AU044698 | -28.69 | 0.038 |
| 1454951_at | zinc finger protein 606 | -6.4 | 0.027 |
| 1455124_at | tripartite motif containing 68 | -9.23 | 0.046 |
| 1455159_at | RIKEN cDNA 2900057D21 gene | -40.86 | 0.025 |
| 1455210_at | zinc fingers and homeoboxes protein 2 | -6.49 | 0.013 |
| 1455324_at | hypothetical LOC433022 | -3.31 | 0.048 |
| 1455331_at | RIKEN cDNA A230102O09 gene | -2.94 | 0.025 |
| 1455406_at | 0 day neonate head cDNA, RIKEN full-length enriched library | -9.73 | 0.002 |
| 1455437_at | cDNA sequence BC033915 | -3.96 | 0.033 |
| 1455448_at | neural stem cell-derived dendrite regulator | -10.75 | 0.017 |
| 1455451_at | expressed sequence AI449310 | -25.8 | 0.041 |
| 1455481_at | iduronate 2-sulfatase | -3.58 | 0.039 |
| 1455536_at | RIKEN cDNA A630023A22 gene | -10.11 | 0.002 |
| 1455577_at | Chemokine (C-C motif) ligand 28 | -6.99 | 0.047 |
| 1455603_at | Transcribed locus | -2.62 | 0.007 |
| 1455878_at | RIKEN cDNA 2700023E23 gene | -13.98 | 0.003 |
| 1455995_at | DNA segment, Chr 10, Brigham & Women's Genetics 1379 expressed | -36.76 | 0.026 |
| 1456110_at | ankyrin repeat domain 11 | -4.06 | 0.028 |
| 1456216_at | Casein kinase 1, alpha 1 | -4.65 | 0.031 |
| 1456253_s_at | pleckstrin homology domain containing, family N member 1 | -6.68 | 0.043 |
| 1456300_at | IlvB (bacterial acetolactate synthase)-like | -4.39 | 0.036 |
| 1456413_at | RIKEN cDNA 9430063L05 gene | -12.18 | 0.049 |
| 1456640_at | SH3 domain containing ring finger 2 | -4.21 | 0.049 |
| 1456738_s_at | brain protein 16 | -6.02 | 0.006 |
| 1457004_at | DNA segment, Chr 15, Wayne State University 169, expressed | -4.39 | 0.025 |
| 1457021_x_at | anti-Mullerian hormone type 2 receptor | -13.75 | 0.039 |
| 1457162_at | low density lipoprotein receptor adaptor protein 1 | -6.55 | 0.043 |
| 1457213_a_at | diacylglycerol kinase, eta | -4.03 | 0.026 |
| 1457256_x_at | patched homolog 2 | -5.84 | 0.048 |
| 1457277_at | cDNA sequence BC038925 | -6.07 | 0.026 |
| 1457299_at | glutamate receptor, metabotropic 4 | -2.33 | 0.044 |
| 1457425_at | Transcribed locus | -2.79 | 0.046 |
| 1457552_at | zinc finger protein 295 | -3.09 | 0.025 |
| 1457593_at | RIKEN cDNA 2610202C22 gene | -7.99 | 0.016 |
| 1457621_at | RIKEN cDNA 2400006N03 gene | -3.07 | 0.021 |
| 1457636_x_at | RIKEN cDNA 2700094F01 gene | -2.15 | 0.025 |
| 1457656_s_at | RIKEN cDNA C230085N15 gene | -21.94 | 0.044 |
| 1457707_at | gene model 489, (NCBI) | -4.65 | 0.012 |
| 1457712_at | chromodomain helicase DNA binding protein 8 | -4.37 | 0.047 |
| 1457760_at | RIKEN cDNA A930004J17 gene | -7.49 | 0.029 |
| 1457943_at | RIKEN cDNA D130067C23 gene | -13.33 | 0.043 |
| 1458016_at | Mm.192356 | -3.19 | 0.013 |
| 1458046_at | 0 day neonate kidney cDNA, RIKEN full-length enriched library | -2.97 | 0.034 |
| 1458149_at | 11 days embryo gonad cDNA, RIKEN full-length enriched library | -3.08 | 0.046 |
| 1458167_at | Potassium voltage-gated channel, Shal-related family, member 2 | -9.86 | 0.011 |
| 1458187_at | Phospholipase C, delta 4 | -5.95 | 0.008 |
| 1458220_at | deleted in liver cancer 1 | -7.08 | 0.025 |
| 1458364_s_at | interferon alpha responsive gene | -16.12 | 0.018 |
| 1458395_at | hypothetical protein B930054O08 | -2.06 | 0.016 |
| 1458400_at | RIKEN cDNA 9630050P21 gene | -4.71 | 0.019 |
| 1458430_at | expressed sequence C87436 | -3.67 | 0.041 |
| 1458567_at | RIKEN cDNA D130017N08 gene | -3.08 | 0.025 |
| 1458652_at | RIKEN cDNA 2410014A08 gene | -3.88 | 0.018 |
| 1458722_at | RIKEN cDNA 1200015F23 gene | -2.46 | 0.041 |
| 1458842_at | Adult male olfactory brain cDNA, RIKEN full-length enriched library | -4.48 | 0.048 |
| 1458848_at | Mm.173487 | -5.52 | 0.018 |
| 1458970_at | RAD50 homolog (S. cerevisiae) | -2.52 | 0.033 |
| 1459257_at | RIKEN cDNA A230048G03 gene | -3.5 | 0.042 |
| 1459416_at | B-cell CLL/lymphoma 7A | -7.61 | 0.025 |
| 1459570_at | G protein-coupled receptor 143 | -6.83 | 0.022 |
| 1459574_at | expressed sequence C86865 | -7.09 | 0.011 |
| 1459600_at | Calcium/calmodulin-dependent protein kinase II, delta | -2.55 | 0.049 |
| 1459832_s_at | adaptor-related protein complex AP-1, mu subunit 1 | -4.75 | 0.011 |
| 1459849_x_at | valosin containing protein (p97)/p47 complex interacting protein 1 | -3.44 | 0.031 |
| 1459853_x_at | CDNA sequence BC031781 | -21.19 | 0.03 |
| 1459861_s_at | F-box and leucine-rich repeat protein 10 | -11.75 | 0.038 |
| 1459869_x_at | RIKEN cDNA 4930402E16 gene | -10.48 | 0.032 |
| 1459873_x_at | Mm.215523 | -36.21 | 0.021 |
| 1459876_at | Mm.152993 | -6.64 | 0.04 |
| 1459905_at | gene model 827, (NCBI) | -2.15 | 0.02 |
| 1459984_at | melanoma inhibitory activity 3 | -27.48 | 0.017 |
| 1460016_at | expressed sequence AW547186 | -25.72 | 0.035 |
| 1460084_at | Mm.214471 | -10.01 | 0.036 |
| 1460097_at | Glutamate receptor, ionotropic, AMPA4 (alpha 4) | -17.51 | 0.008 |
| 1460150_at | SH2 domain containing 3C | -2.81 | 0.026 |
| 1460151_at | Mm.173314 | -4.33 | 0.032 |
| 1460452_at | motile sperm domain containing 3 | -3.08 | 0.044 |
| 1460567_at | RIKEN cDNA 9930116O05 gene | -4.42 | 0.027 |
| 1460620_at | zinc finger protein 592 | -20.82 | 0.018 |
